# Core nucleosomes are refractory to lentiviral DNA integration

**DOI:** 10.1038/s41467-026-76698-8

**Published:** 2026-08-18

**Authors:** Joshua Hope, Emma Punch, Nicola J. Cook, Matthew R. Singer, Dhira Joshi, Parmit K. Singh, Andrea Nans, Nathan P. Sweeney, Willem Vanderlinden, Alan N. Engelman, Peter Cherepanov

**Affiliations:** 1 Chromatin Structure & Mobile DNA Laboratory, The Francis Crick Institute, London, UK; 2 Chemical Biology Science Technology Platform, The Francis Crick Institute, London, UK; 3 Department of Cancer Immunology and Virology, Dana-Farber Cancer Institute, Boston, MA USA; 4 Structural Biology Science Technology Platform, The Francis Crick Institute, London, UK; 5 Cell and Gene Therapy, Medicines Research Centre, GSK, Stevenage, UK; 6 School of Physics and Astronomy, University of Edinburgh, Edinburgh, UK; 7 Department of Infectious Disease, South Kensington Campus, Imperial College London, London, UK; 8 Present Address: Structural Biology of Human Pathogens, The University of Oxford, Oxford, UK; 9 Present Address: Oxford Biomedica (UK) Ltd., Oxford, UK

**Keywords:** Cryoelectron microscopy, Retrovirus, DNA

## Abstract

HIV-1 and other lentiviruses hijack the cellular chromatin-binding protein LEDGF/p75 to facilitate integration into active transcription units. However, the mechanism of chromatin engagement by lentiviral intasomes and the structural role of LEDGF/p75 in this process remain poorly understood. To address these gaps, we studied the activities of native HIV-1 preintegration complexes and in vitro-assembled lentiviral intasomes in the presence of chromatinized target DNA. While LEDGF/p75 was both essential and minimally sufficient to enhance lentiviral integration into chromatin containing histone H3 trimethylated on Lys36, it unexpectedly facilitated integration outside of the nucleosome core particles. LEDGF/p75 additionally inhibited integration into unmodified chromatin in a dose-dependent manner, promoting integration into naked DNA. To explore the structural foundation for these activities, we imaged maedi-visna virus intasomes saturated with LEDGF/p75 before and after strand transfer by cryogenic electron microscopy. The structures revealed that the host factor alters the target DNA binding platform of the lentiviral intasome, imposing significant constraints on the path and configuration of target DNA to impede nucleosome engagement. Our results establish the preference of lentiviral intasomes for linker DNA regions within H3K36Me3-enriched chromatin and show that LEDGF/p75 plays a specific structural role at the viral–host target DNA interface.

## Abstract (web summary)

Here, the authors demonstrate that LEDGF/p75 directs lentiviral integration into linker DNA within H3K36me3 chromatin while inhibiting integration into unmodified chromatin. Cryo-EM structures reveal that the host factor profoundly reshapes the intasome target DNA binding platform.

## Introduction

For retroviral infection to proceed, the reverse transcribed viral DNA (vDNA) must be inserted into the infected host cell’s genome. This process is catalyzed by a multimer of integrase (IN), a viral enzyme that assembles on synapsed vDNA ends to form the functional nucleoprotein complex termed the intasome. Following the initial processing of 3’ vDNA ends, the intasome captures host DNA (referred to as target DNA; tDNA) to carry out a pair of trans-esterification events, joining both 3’ vDNA ends to opposing strands of tDNA^1^. Notwithstanding the large body of biochemical and structural studies that elucidated the mechanism of integration, how the intasome gains access to tDNA in the context of host chromatin remains poorly understood.

Structural studies revealed that retroviral intasomes may contain from 4 to as many as 16 IN subunits, with the largest assemblies reported for lentiviruses^2–13^. Despite this variability in composition, only two IN subunits provide the active sites necessary for catalysis, which occurs without significant changes to the overall structure of the intasome^12,14^. For clarity, after 3’-processing but before tDNA engagement, the intasome is referred to as the cleaved synaptic complex (CSC), while the post-catalytic form of the intasome, with 3’ vDNAs covalently joined to tDNA, is called the strand transfer complex (STC). During viral infection, the intasome is part of a much larger nucleoprotein complex known as the preintegration complex (PIC), which contains ~9–10 kb vDNA, IN, and a variety of incompletely characterized host-derived components^15–18^. Integration of 3’ vDNA ends occurs in a staggered fashion across the major groove, which is expanded through sharp deformations of tDNA^2,19^. Following gap repair by host enzymes, the resulting provirus is flanked by short target site duplications derived from the cleaved tDNA sequence^20,21^. Of relevance to this study, the lentiviruses HIV-1 and maedi-visna virus (MVV) integrate with 5- and 6-bp staggers, respectively, while the prototype foamy virus (PFV), a well-characterized model from the Simiispumavirus genus, with a 4-bp stagger^2,8,19–21^.

Retroviral integration does not occur randomly and exhibits distinct genus- and species-specific patterns across multiple genomic scales, from local tDNA sequence to broader contexts such as genes and nuclear architecture^22,23^. While active transcription units are preferred targets for lentiviral integration, spumaviruses favor intergenic regions, including lamina-associated heterochromatin^24–28^. At even larger scales, integration distributions are influenced by factors including gene density and association with nuclear speckles^23,29^. While IN–tDNA interactions dictate local sequence preferences^2,19,30,31^, cellular proteins govern genome-level integration targeting (reviewed in refs. ^22,32^). Lentiviruses, in particular, are directed to integrate into the bodies of highly expressed genes through the interaction between IN and the transcriptional co-activator LEDGF/p75^33–38^. This host factor contains an N-terminal PWWP chromatin reader domain, with binding preference for nucleosomes containing histone H3 tri-methylated on Lys36 (H3K36Me3)^39–41^ and a C-terminal IN-binding domain (IBD), which interacts with HIV-1 and other lentiviral INs^42–44^. LEDGF/p75 strongly promotes lentiviral IN catalytic activity and facilitates lentiviral intasome assembly in vitro^8,9,44^. H3K36Me3 is enriched in active transcription units^45^, and according to the prevailing paradigm, LEDGF/p75 tethers IN to chromatin for integration. Concordantly, ablation of LEDGF/p75 in host cells leads to significant loss of lentiviral integration into the bodies of expressed genes with a pattern shift in remaining integrations towards upstream gene regions^34,36–38^. Ablation of SETD2, the methyltransferase responsible for trimethylation of histone H3 Lys36, similarly redistributes HIV-1 integration sites toward upstream gene regions^46^. Moreover, replacement of the LEDGF/p75 PWWP domain with alternative chromatin reader domains is sufficient to redirect HIV-1 integration to genomic regions enriched in the corresponding histone modifications^47^. The expanded architecture of lentiviral intasomes permits recruitment of up to 16 LEDGF/p75 molecules, a feature proposed to enhance recognition of H3K36Me3-containing chromatin regions^12^. Consistent with this idea, artificial multimerization of chromatin reader domains has been shown to increase their avidity for histone post-translational modifications^48–50^.

The basic unit of chromatin structure, the nucleosome, greatly restricts accessibility of the DNA major groove^51^. Conversely, widening of the major groove, where it is solvent-exposed on the nucleosome surface, could offer a preferred bent tDNA for integration^52–54^. Indeed, PFV intasomes display remarkable selectivity for nucleosomes, preferentially targeting superhelical locations (SHL) ± 3.5^27,55^. While these positions offer the widest available major groove on a nucleosome core particle, nucleosomal DNA undergoes further deformation for PFV integration. Thus, the highly bent tDNA configuration observed during PFV integration into naked DNA was recapitulated in the context of a nucleosome^27,55^. For other retroviruses, including HIV-1, the literature is largely conflicting, with reports of increased integration into chromatinized^52,53,56–59^ or naked DNA^56^. Notably, most of these early studies relied on poorly characterized IN-vDNA complexes. More recently, it was reported that HIV-1 PICs extracted from infected cells integrate preferentially into naked DNA substrates, rather than mononucleosomes^60^. While the C-terminal domain (CTD) of HIV-1 IN reportedly interacts with histone H4 to strongly stimulate integration catalysis in vitro, structural studies have yet to define the interaction of lentiviral intasomes with nucleosomes^61^. Despite overwhelming evidence for the role of LEDGF/p75 in directing lentiviral integration into the bodies of actively transcribed regions, the mechanism by which this occurs remains poorly understood.

Herein, we investigated the interactions of native HIV-1 PICs and in vitro-assembled lentiviral intasomes with well-defined and heterogenous chromatinized tDNA. Utilizing Oxford Nanopore Technologies sequencing, we enumerated integration sites along polynucleosomal arrays to define the role of LEDGF/p75 in targeting integration into H3K36Me3-modified chromatin. Using cryo-EM, we demonstrate that the host factor reshapes the tDNA-binding face of the lentiviral intasome, imposing specific constraints on its interactions with target chromatin. Our results explain how LEDGF/p75 guides lentiviral integration preferentially into linker DNA within H3K36Me3-enriched chromosomal domains.

## Results

### HIV-1 PICs preferentially target chromatin containing H3K36Me3

**Fig. 1:**
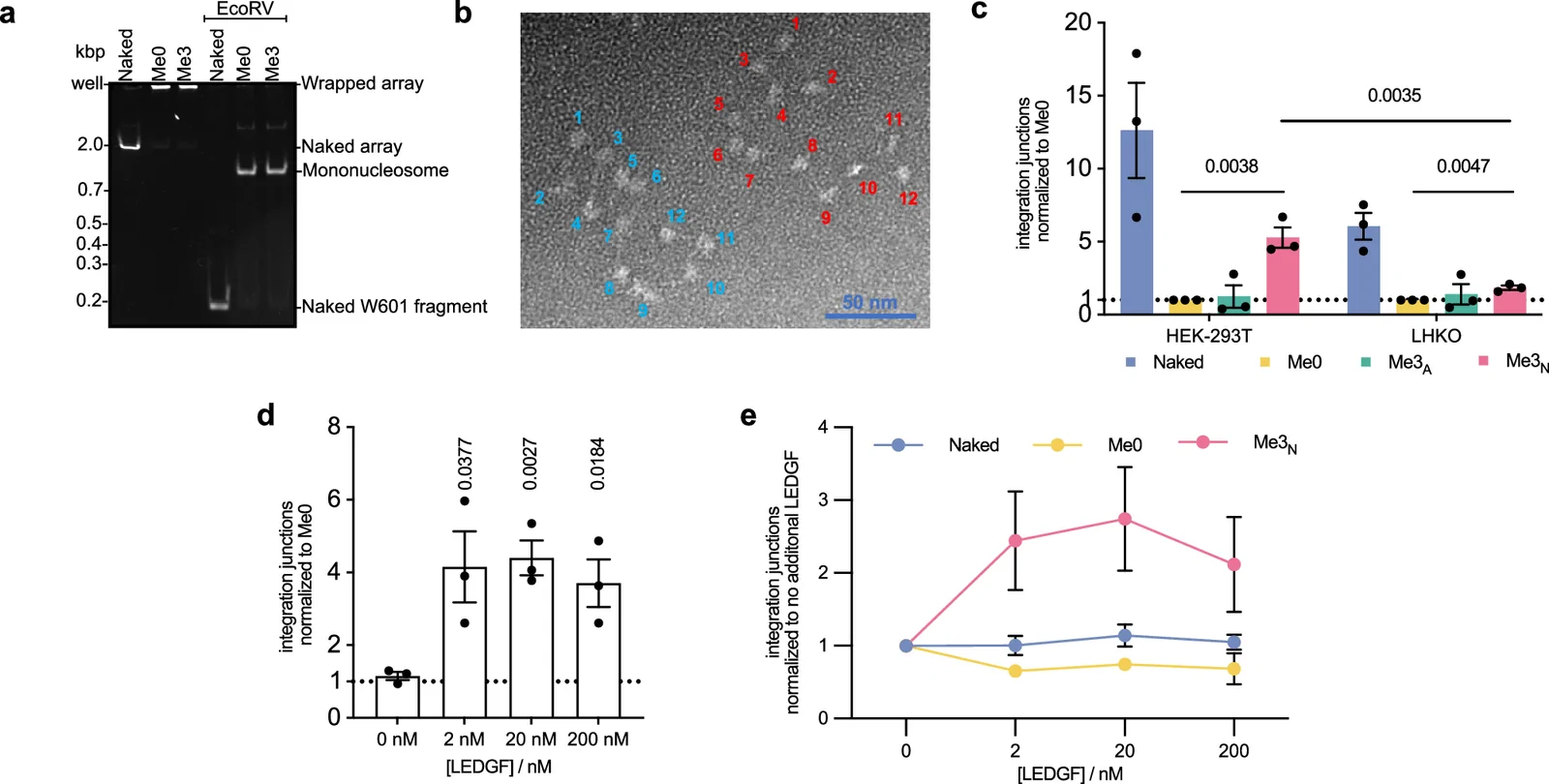
Strand transfer activity of HIV-1 PICs with polynucleosomal arrays. **a** Validation of H3K36Me0 and H3K36Me3 (indicated as Me0 and Me3, respectively) polynucleosomal arrays by native PAGE. Uncut naked DNA migrated at 2.3 kb, whereas the polynucleosomal array remained in the wells of the gel. To assess the efficiency of wrapping, the arrays were digested by EcoRV, which cuts the array in the middle of each linker segment, releasing 191-bp fragments (Fig. S1a). DNA was detected by ethidium bromide staining in the presence of 0.2% SDS. Migration positions of various molecular species and size markers (kbp) are shown to the right and left of the gel, respectively. Gel image is representative of a typical polynucleosomal array assembly, which was repeated at least 5 times in this study. **b** Polynucleosomal arrays visualized by negative stain EM; the representative micrograph captures two copies of the K36Me0 array. Individual nucleosomes are indicated with blue or red numbers (1–12); scale bar is 50 nm. Negative stain electron microscopy was done for at least three independent array assemblies. **c** Integration activity of HIV-1 PICs isolated from HEK293T or LEDGF/p75 and HRP2-null (LHKO) cells into naked or polynucleosomal arrays. The arrays were assembled with unmodified (Me0) or H3K36Me3 nucleosomes using the methyl-lysine analog (Me3_A_) or native (Me3_N_) modification. The bar plots display strand transfer activity quantified by real-time PCR, relative to the Me0 condition for each PIC isolate. Error bars represent mean ± SEM; *n* = 3 independent strand-transfer experiments. Individual measurements are shown as black circles. Pairwise comparisons are a two-sided Student’s *t*-test with relevant *p*-values indicated on the plot. **d**, **e** Integration activity of PICs derived from LHKO cells into Me3 polynucleosomal arrays, normalized to activity in Me0 arrays with given concentrations of recombinant LEDGF/p75. Error bars represent mean ± SEM; *n* = 3 independent strand-transfer experiments. Individual measurements in (**d**) are shown as black circles. Comparisons to 0 nM LEDGF/75 condition are two-sided Student’s *t*-test with *p*-values indicated above each bar. Source data are provided as a Source Data file.

To investigate the role of tDNA chromatinization on HIV-1 PIC activity, we assembled regular polynucleosomal arrays on a linear 2.3-kb DNA substrate comprising 12 repeats of the Widom 601 (W601) positioning sequence^62^ separated by 44 bp of linker DNA (Supplementary Fig. 1) to model the 43–48 bp spacing observed for active gene bodies in human T cells^63^. The assembled chromatin contained specific variations with respect to histone H3: (i) unmodified (Me0), (ii) a methyl-lysine analog mimic of H3 tri-methylated on Lys36 (H3K36Me3_A_, produced by alkylation of a Cys residue substituting for the natural Lys36), or (iii) native tri-methylated Lys36 (H3K36Me3_N_). The arrays were assessed for quality of nucleosome formation by native PAGE following linker digestion with a restriction enzyme (Fig. 1a) and by negative-stain electron microscopy (Fig. 1b).

We incubated the polynucleosomal and naked W601 tDNA arrays with PICs extracted from the nuclei of cells infected with an HIV-1-derived lentiviral vector and quantified the products of strand transfer by real-time quantitative PCR (qPCR). The results revealed that the PICs strongly preferred naked tDNA over the polynucleosomal array containing non-modified H3 (Fig. 1c). Strikingly, H3K36Me3_N_, but not the methyl-lysine analog H3K36Me3_A_, substantially stimulated integration into chromatinized DNA. Because lentiviral PICs incorporate cellular LEDGF/p75^64^, we reasoned that the host factor may be responsible for the enhanced integration into the H3K36Me3_N_-containing array. Concordantly, the methylation status of the chromatin did not appreciably improve the integration efficiency of PICs derived from LEDGF/HRP2-null HEK293T cells (LHKO; Fig. 1c), and the preference for H3K36Me_N_ chromatin was importantly rescued by the addition of recombinant LEDGF/p75 protein (Fig. 1d, e). Strikingly, the increase of PIC activity on H3K36Me3_N_ chromatin was concomitant with a reduction of integration activity into unmodified chromatin in the presence of the host factor at sub-stoichiometric (2 nM) and super-stoichiometric (20 and 200 nM) concentrations with respect to the nucleosome concentration of 6 nM (Fig. 1e). These results demonstrate that cellular LEDGF/p75, and possibly its close homolog HRP2^42,43,65^, facilitate integration of HIV-1 PICs into H3K36Me3_N_-containing chromatin^40,66,67^. By contrast, H3K36Me3_A_ failed to stimulate PIC activity in these assays, consistent with the comparatively poor binding of the methyl-lysine analog to LEDGF/p75^68^.

### LEDGF/p75 renders lentiviral intasomes specific for H3K36Me3 chromatin

**Fig. 2:**
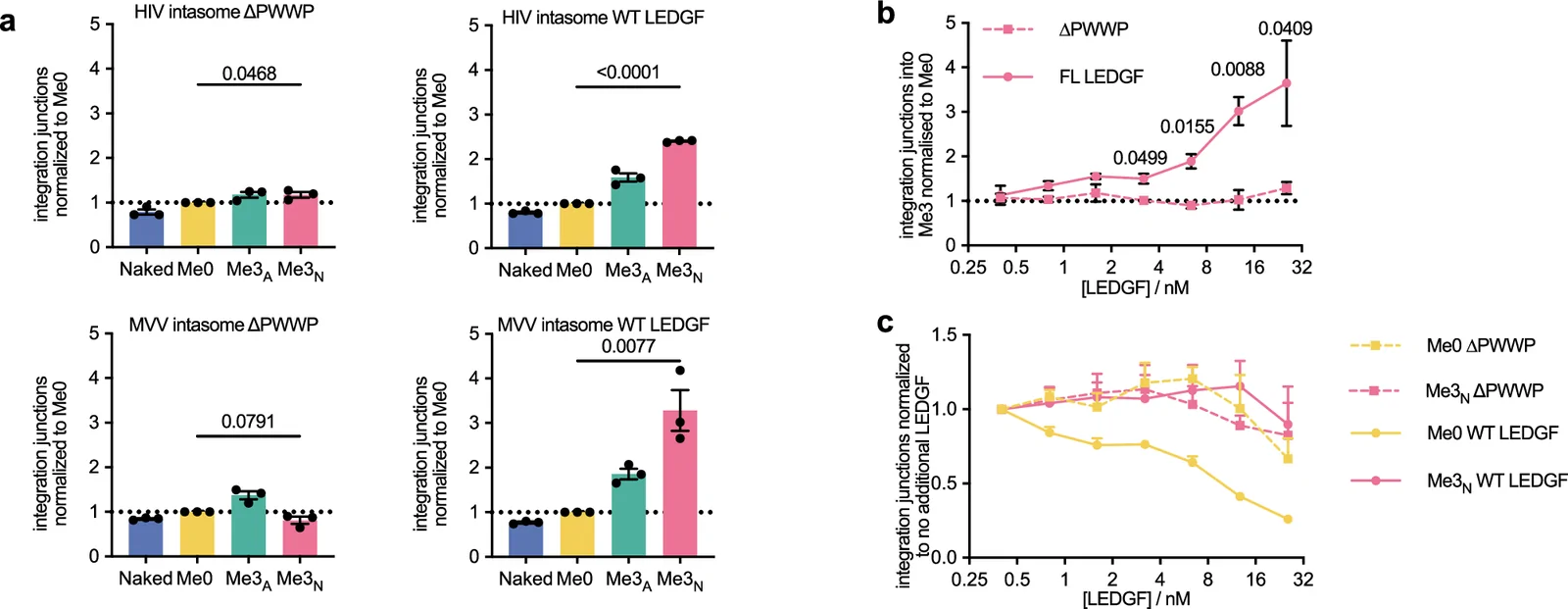
Strand transfer activity of lentiviral intasomes into polynucleosomal arrays. **a** Integration of lentiviral intasomes into naked or polynucleosomal arrays assembled with unmodified (Me0) or H3K36Me3 nucleosomes modified by the methyl-lysine analog (Me3_A_) or native chemical ligation (Me3_N_) approach. HIV-1 (top row) and MVV (bottom row) intasomes were assembled in the presence of full-length LEDGF/p75 (WT LEDGF, right) or LEDGF/p75 lacking the PWWP domain (ΔPWWP, left). Integration of HIV-1 intasomes into Me3_N_ polynucleosomal arrays. Data normalized either to activity in Me0 arrays at the indicated concentrations of recombinant LEDGF/p75 or ∆PWWP LEDGF (**b**), or to the baseline condition (0.4 nM LEDGF/p75) in the absence of additional supplementation by the host factor (**c**). Error bars represent mean ± SEM; *n* = 3 independent strand-transfer experiments. Pairwise comparisons are two-sided Student’s *t*-test with relevant *p*-values indicated above each data point. Source data are provided as a Source Data file.

The assembly of lentiviral intasomes in vitro is greatly promoted by LEDGF/p75^8–10^, although strand transfer competent nucleoprotein complexes can be formed in the absence of the host factor using a chimera of HIV-1 IN with the archaeal DNA binding protein Sso7d (Sso7d-IN)^10,69^. To establish minimal requirements for the selectivity of lentiviral integration into H3K36Me3 chromatin, we formed HIV-1 and MVV intasomes using wild-type (WT) recombinant INs and short oligonucleotide mimics of vDNA ends in the presence of LEDGF/p75 (Supplementary Fig. 2). We included the MVV intasome in key experiments alongside the HIV-1 intasome for comparison, as MVV readily forms discrete, homogeneous complexes in vitro, whereas HIV-1 intasomes are comparatively polydisperse^8,9,70^. To replicate conditions of our experiments with HIV-1 PICs, we incubated highly diluted intasome assemblies with excess naked or chromatinized tDNA and quantified strand-transfer products using qPCR. Although we could not directly quantify the efficiency of intasome assembly, the maximal intasome concentration was 0.025 nM, ensuring that tDNA was in large excess. Nonetheless, intasomes generated ~100–1000-fold more integration junctions than native HIV-1 PICs, reflecting the scarcity of the latter. Unlike HIV-1 PICs, the in vitro-assembled lentiviral intasomes did not discriminate against chromatinized DNA (Fig. 2a). However, the integration efficiencies were markedly higher in the presence of H3K36Me3_N_ compared to non-modified polynucleosomal arrays (Fig. 2a). Moreover, H3K36Me3_N_ significantly outperformed the methyl-lysine analog H3K36Me3_A_ at promoting strand transfer into chromatinized tDNA. By contrast, both lentiviral intasomes assembled with an LEDGF/p75 mutant lacking the PWWP domain (LEDGF-ΔPWWP) lost the preference for the modified chromatin, directly implicating the host factor in the recognition of the epigenetic mark (Fig. 2a). Supplementing HIV-1 intasomes with increasing concentration of LEDGF/p75 revealed stronger discrimination against non-modified chromatin in a dose-dependent manner, where the nucleosome concentration was 6.3 nM, and the LEDGF/p75 concentration ranged from 0.4 to 26 nM (Fig. 2b, c). As expected, HIV-1 intasomes formed using the Sso7d-IN fusion in the absence of LEDGF/p75 lacked the specificity for H3K36Me3 chromatin (Supplementary Fig. 3).

### HIV-1 intasomes predominantly integrate into linker DNA regions within a nucleosomal array

To accurately determine the integration sites of intasomes on polynucleosomal arrays, we optimized a PCR-free integration site mapping method using long-read Oxford Nanopore Technologies sequencing. We chose to avoid PCR amplification, which can introduce ambiguity in distinguishing between amplification duplicates and multiple integration events at any single position within the array. Additionally, we sought a method capable of assigning integration sites to individual W601 repeats, which required long-read sequencing. To achieve these goals, intasomes were formed with vDNA oligonucleotides carrying a dA overhang at the 3’ end of the unreactive strand and phosphorylation at the 5’ end of the reactive strand, making them compatible with Nanopore sequencing adapters. After purifying intasomes from excess vDNA, this design allowed selective sequencing of strand-transfer products while excluding unreacted tDNA.

**Fig. 3:**
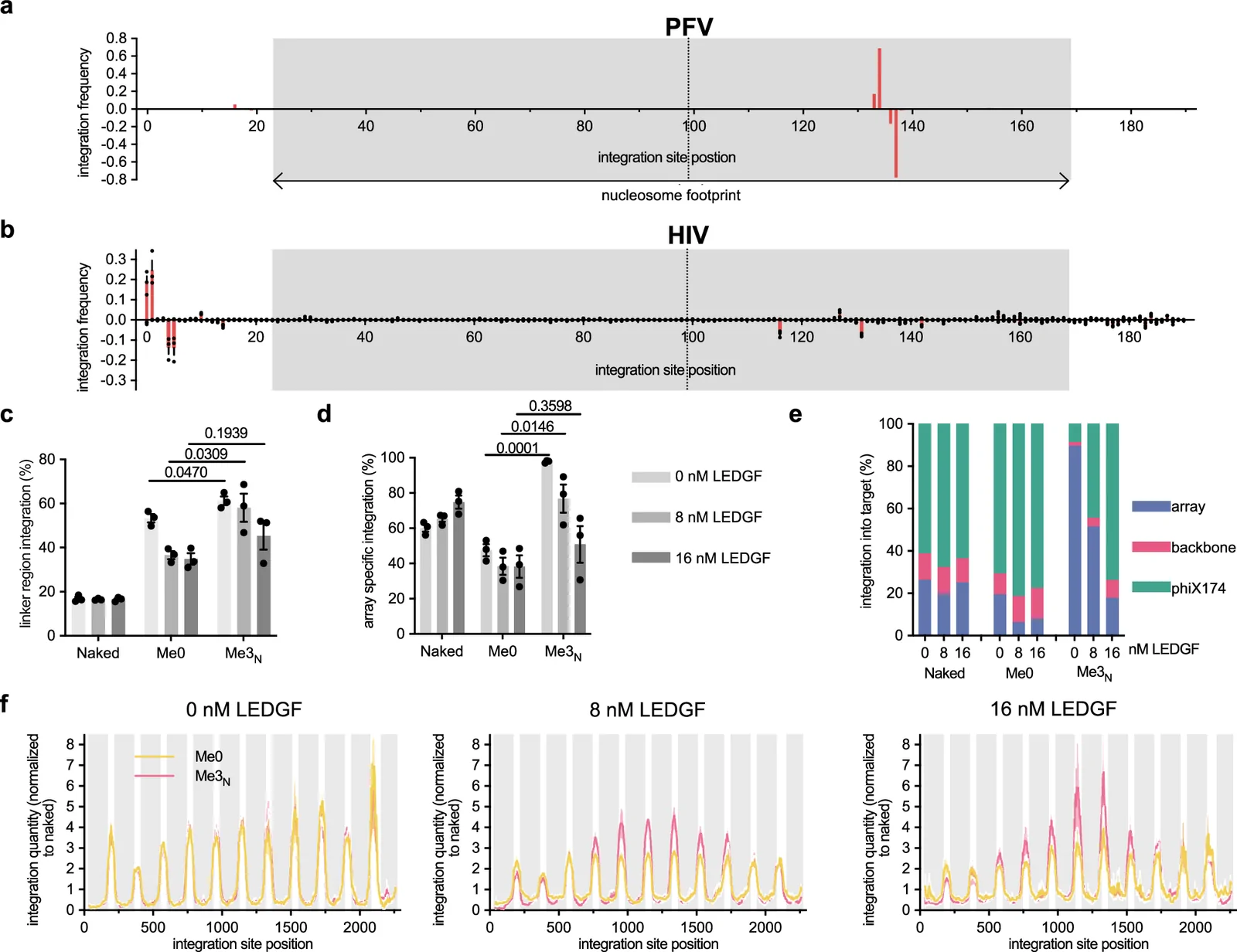
Mapping intasome integration sites into regular polynucleosomal arrays. Maps of PFV (**a**) and HIV-1 (**b**) intasomal integration sites, the latter in the presence of 8 nM LEDGF/p75, into H3K36Me3_N_-modified polynucleosomal arrays. Positive and negative values represent vDNA insertion frequencies at specific positions on the forward and reverse strands, respectively. The data were aggregated from the entire polynucleosomal array compressed onto a single repeating unit. Gray boxes indicate the footprint of the nucleosome core particle; the dotted line represents the symmetrical dyad position of the nucleosome. Error bars (gray lines) represent mean ± SEM; *n* = 3 independent strand-transfer experiments. Individual measurements in (**b**) are shown as black circles. **c** Percentage of total HIV-1 intasome integration sites that targeted the linker regions of naked, H3K36Me0, or H3K36Me3_N_ tDNA arrays in the presence of 0, 8, or 16 nM LEDGF/p75. **d** Percentage of HIV-1 integration sites aligning to polynucleosomal array over contaminating vector backbone in the presence of indicated LEDGF/p75 inputs for naked, H3K36Me0 (Me0), or H3K36Me3 (Me3_N_) chromatinized templates. **e** Identical experiment to panel d, except reactions were spiked with naked phage phiX174 DNA. Bars display the percentage distribution of integration into each target for naked or polynucleosomal arrays chromatinized with unmodified (Me0) or H3K36Me3 (Me3_N_) histones. **f** Integration site distribution for HIV-1 intasomes analyzed across the polynucleosomal array with 12 repeating nucleosome positioning units. Data are normalized to integration into the naked DNA substrate under the same given concentrations of LEDGF/p75. Rolling averages are plotted for unmodified (Me0, yellow) and H3K36Me3_N_-modified (Me3_N_, pink) chromatin. Gray shading indicates the nucleosomal footprints. Error bars (**c**– **f**) represent mean ± SEM; *n* = 3 independent strand-transfer experiments. Pairwise comparisons are two-sided Student’s *t*-test with relevant *p*-values indicated above each data point. Source data are provided as a Source Data file.

First, we validated this approach using the PFV intasome, which has been well-studied biochemically and structurally^2,3^. The spumaviral intasome has a pronounced preference for nucleosome core particles, integrating into SHL ±3.5 positions, with some dependence on the local tDNA sequence^27,55^. We incubated naked or chromatinized array tDNA with purified PFV intasomes, and the strand transfer reaction products were processed for barcoding, adapter ligation, and sequencing in a Nanopore flow cell, which resulted in the recovery of a total of 13–120 thousand integration junctions per sample. In agreement with previous reports, in the presence of chromatinized tDNA, integration overwhelmingly took place at a single position on the W601 nucleosome 35 bp from the nucleosome dyad position (Fig. 3a and Supplementary Fig. 4; the plots show integration sites aggregated into a single array repeat comprising the W601 and flanking linker DNA sequences).

Next, we assembled HIV-1 intasomes using WT IN in the presence of LEDGF/p75 and purified them by size exclusion chromatography to remove excess vDNA oligonucleotides. As expected, the intasomes integrated promiscuously across the naked tDNA, with some non-uniformity due to innate local target-sequence preferences (Supplementary Fig. 5)^71^. Remarkably, when chromatinized arrays were used, a considerably higher proportion of integration sites mapped to the linker DNA regions (Fig. 3b, c and Supplementary Fig. 5). Thus, the fraction of HIV-1 integration sites into linker DNA regions increased from 15 ± 1% in the naked to 53 ± 2% and 61 ± 2% within the non-modified and H3K36Me3_N_-containing polynucleosomal arrays, respectively (Fig. 3c). Akin to HIV-1 PICs, lentiviral intasomes retain LEDGF/p75 during purification (Supplementary Fig. 2b, d), although the occupancy of host factor binding sites on the intasome remains incompletely defined^8,12^. However, supplementation of purified HIV-1 intasomes with 8 or 16 nM LEDGF/p75 (in both cases representing a large excess to the 0.125 nM intasome input and a comparable 6 nM nucleosome input) did not dramatically alter the integration site maps (Supplementary Fig. 5). We note that while excess LEDGF/p75 allowed more integration into non-modified chromatinized tDNA, H3K36Me3_N_-containing chromatin comparatively resisted this redistribution (Fig. 3c).

### LEDGF/p75 massively channels HIV-1 integration into H3K36Me3 containing nucleosomal arrays

While the majority of HIV-1 intasome integration sites were found in the linker segments, we did observe some infiltration into the nucleosome core. The positions of these sites closely mirrored those found on the naked array (Supplementary Fig. 5). The structure of the nucleosome core particles greatly limits the accessibility of tDNA for integration. Indeed, some 80% of the major groove either fully or partially faces histones, or is obscured by the packing of DNA gyres^67^. Yet, we consistently observed integration events into positions that should be fully protected on chromatinized samples. For example, integration position 127 on the forward strand directly faces the histone octamer (Fig. 3b, Supplementary Fig. 5)^67^. Integration into these presumably illicit sites was more pronounced within the unmodified polynucleosomal array (Supplementary Fig. 5). We reasoned that because HIV-1 intasomes strongly disfavored unmodified chromatin, the bulk of integration observed within W601 regions could have occurred in the context of a small fraction of unwrapped regions within the chromatin populations. Concordantly, although the array fragment was gel-purified, we also observed integration events into the bacterial plasmid backbone in our samples. Because bacterial DNA is comparatively refractory to chromatinization^72^, the contaminating backbone DNA was likely poorly populated by nucleosomes. When HIV-1 intasomes were supplied with the non-modified polynucleosomal array, approximately half of the integration sites were found within the plasmid backbone (Fig. 3d). By contrast, in the presence of H3K36Me3_N_, nearly all integration occurred within the array. Based on these observations, we doped the polynucleosomal arrays with non-chromatinized phage phiX174 DNA. This experiment confirmed that the array was refractory to HIV-1 integration, especially when assembled with non-modified H3, and that H3K36Me3_N_ was a preferred target (Fig. 3e). This effect was dependent on the level of LEDGF/p75, the excess of which suppressed integration into chromatinized DNA, although in all conditions H3K36Me3_N_ outperformed non-modified chromatin (Fig. 3d, e).

### LEDGF/p75 directs HIV-1 integration towards the center of the H3K36Me3 polynucleosomal array

During infection, retroviral integration takes place in the context of continuous chromatin, rather than single isolated nucleosomes. Our polynucleosomal array was designed to maximally represent this condition, and the use of long-read Nanopore sequencing allowed us to visualize the global distributions of HIV-1 integration sites across the entire 2.3-kb tDNA. We counted HIV-1 intasome integration sites into the chromatinized arrays at each position and normalized the results by the integration frequency observed with the naked tDNA. As expected for both H3K36Me0 and H3K36Me3_N_-chromatinized templates, integration was overwhelmingly targeted towards the linkers between the nucleosomes across the arrays (Fig. 3f). Strikingly, the addition of exogenous LEDGF/p75 led to progressive accumulation of integration sites towards the center of the H3K36Me3_N_-containing array (though still in the linker DNA regions), while integration into unmodified chromatin remained uniform (Fig. 3f). These observations strongly indicate that the presence of multiple neighboring nucleosomes stimulates HIV-1 IN activity in LEDGF/p75- and H3K36Me3-dependent manners.

### Lentiviral intasomes avoid core nucleosomal regions within chromatin assembled on native eukaryotic DNA

**Fig. 4:**
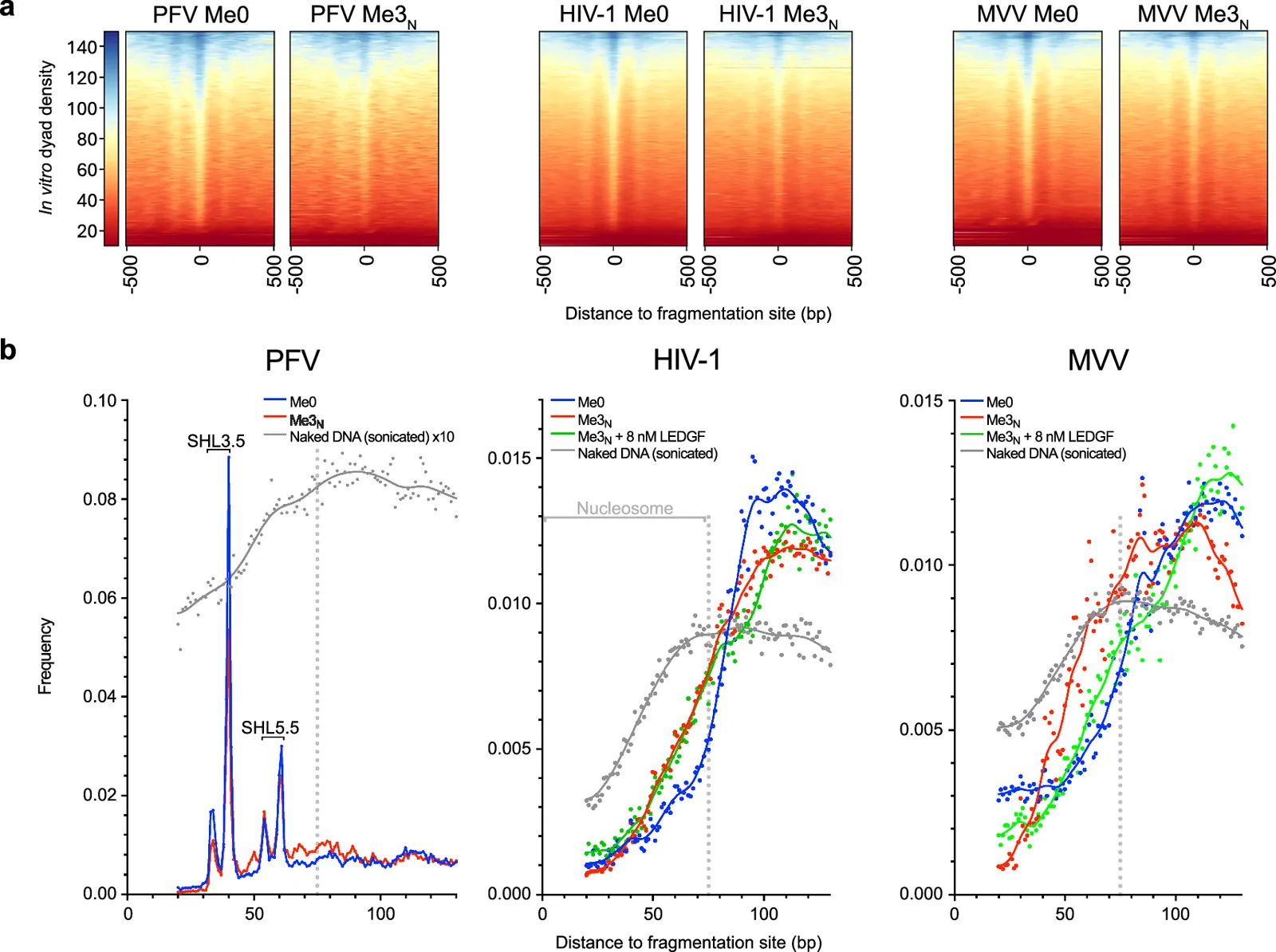
Intasomal integration sites into chromatinized natural DNA fragments. **a** Heatmaps of yeast nucleosome dyads reported by Oberbeckmann et al.^78,79^ with those observed in the present work through copper-induced cleavage and LM-PCR amplification of PFV, HIV-1, or MVV (left-to-right) strand transfer products. The nucleosomes were assembled on genomic yeast DNA fragments with unmodified (Me0, left panels in each pair) or H3K36Me3_N_ (Me3_N_, right panels) histones. **b** Histograms of distances between chemical fragmentation point and intasome integration sites on unmodified (blue), H3K36Me3_N_ (red), and H3K36Me3_N_ with additional LEDGF/p75 (green). Gray lines indicate control experiments in which integration occurred on naked DNA and was subsequently sonicated to create random shearing in place of dyad-specific chemical fragmentation. The vertical dotted gray line indicates the boundary of the nucleosome. Mapped PCR fragments for each are enumerated in Supplementary Table 1; distances under 150 bp were used to construct the histograms (see the “Methods” section for more details). Source data are provided as a Source Data file.

To further investigate how lentiviral intasomes are influenced by tDNA chromatinization, and to disentangle the effects of chromatin structure from local sequence biases, we optimized a method to concurrently map integration sites and nucleosome positions in the context of complex tDNA. To this end, we combined a chemical DNA fragmentation technique, previously employed for single base-pair resolution mapping of yeast nucleosomes^73,74^, with ligation-mediated PCR (LM-PCR), a standard tool for mapping retroviral integration sites^24,75^. We assembled nucleosomes on *S. cerevisiae* genomic DNA with human histones, including H4 carrying the S47C amino acid substitution, which was conjugated to N-(1,10 phenanthrolin-5-yl) iodoacetamide. Budding yeast DNA was chosen because of its comparatively high propensity to position nucleosomes^76^ and scarcity of repetitive regions. The chromatinized templates were validated for wrapping consistency by micrococcal nuclease digestion (Supplementary Fig. 6a) and by atomic force microscopy, which revealed the expected beads-on-a-string pattern (Supplementary Fig. 6b). We incubated the chromatinized DNA with PFV, HIV-1, or MVV intasomes and subsequently supplemented the reactions with Cu^2+^ ions and 3-mercaptopropionic acid. The resulting Fenton reaction triggers cleavage of nucleosomal DNA strands at positions −1 or +6 bp relative to the dyad base pair (position 0), representing what is referred to as the primary and the secondary fragmentation site, respectively^73,77^. Following end-repair, LM-PCR afforded the recovery of strand transfer products that occurred in relative proximity to nucleosomes. Sequencing of the amplified fragments revealed a clear correlation of our copper-phenanthroline yeast DNA fragmentation sites with nucleosome midpoints observed in an unrelated study that used micrococcal nuclease for nucleosome mapping in in vitro assembled yeast chromatin (Fig. 4a)^78,79^. Analysis of the lengths of yeast genomic segment captured between the PFV vDNA end and the linker showed a predominant peak at 40 bp, aligning with major PFV integration sites 36 bp from dyads, and primary fragmentation at −1 bp sites, while the satellite peak at 33–34 bp was consistent with Cu^2+^-phenanthroline assisted tDNA fragmentation at the secondary site (Fig. 4b, Supplementary Fig. 6c). This analysis also revealed a less pronounced doublet of peaks at 54 and 61 bp, suggesting that a subset of PFV integration events occur two DNA turns further away from the dyad (corresponding to SHL ± 5.5 positions). Largely similar patterns were observed when PFV intasomes facilitated integration into H3K36Me3 chromatin, consistent with the lack of H3K36Me3 recognition by PFV intasomes (Fig. 4b).

In stark contrast, strand transfer reactions with HIV-1 and MVV intasomes led to amplification of chromosomal DNA segments longer than half the nucleosome length (73 bp), without distinct peaks within the span of a nucleosome core particle (Fig. 4b). We considered that detection of integration sites closer to the site of fragmentation dyad may be reduced due to less efficient recovery on magnetic beads, Nanopore base calling, and/or mappability of shorter DNA fragments. To evaluate these possibilities, we incubated the intasomes with naked yeast DNA that was randomly sheared by sonication, and mapped integration sites in the vicinity of sheared sites using the same approach (but omitting DNA fragmentation by Fenton chemistry). The resulting distribution of yeast genomic DNA fragments confirmed progressive depletion of chromosomal segments under 50 bp (Fig. 4b). Nevertheless, the suppression was markedly more pronounced within chromatinized DNA (*χ*²(129) = 10,656, *p* < 0.0001, Cramér’s *V* = 0.261, 95% CI [0.256, 0.264] and *χ*²(129) = 6,216, *p* < 0.0001, Cramér’s *V* = 0.185, 95% CI [0.179, 0.188] for HIV-1 and MVV, respectively). Interestingly, compared to non-modified chromatin, H3K36Me3-containing nucleosomes were slightly more receptive for lentiviral integration, allowing more events within entry/exit regions (*χ*²(129) = 1898, *p* < 0.0001, Cramér’s *V* = 0.091, 95% CI [0.085, 0.092] and *χ*²(129) = 3213, *p* < 0.0001, Cramér’s *V* = 0.164, 95% CI [0.156, 0.167] for HIV-1 and MVV, respectively). This observation is consistent with recent findings that H3K36Me3 promotes nucleosome breathing, facilitating transient unwrapping of the outer DNA segments^80,81^. The effect was likely masked in our experiments using W601 arrays due to local tDNA sequence preferences of the intasomes. Supplementation of HIV-1 intasomes with 8 nM LEDGF/p75 did not result in appreciable shifts in HIV-1 integration site distributions on H3K36Me3 chromatin, whereas MVV integration sites shifted further away from the dyad (*χ*²(129) = 1521, *p* < 0.0001, Cramér’s *V* = 0.142, 95% CI [0.129, 0.143]). One possible explanation is that the greater heterogeneity of HIV-1 intasome preparations relaxed structural constraints, permitting increased integration into nucleosome core regions upon LEDGF/p75 saturation. Nonetheless, these results confirm and extend our observations from the W601 arrays: in contrast to PFV, lentiviral intasomes integrate predominantly outside nucleosome core particles.

### LEDGF/p75 reshapes the lentiviral intasome tDNA binding platform

**Fig. 5:**
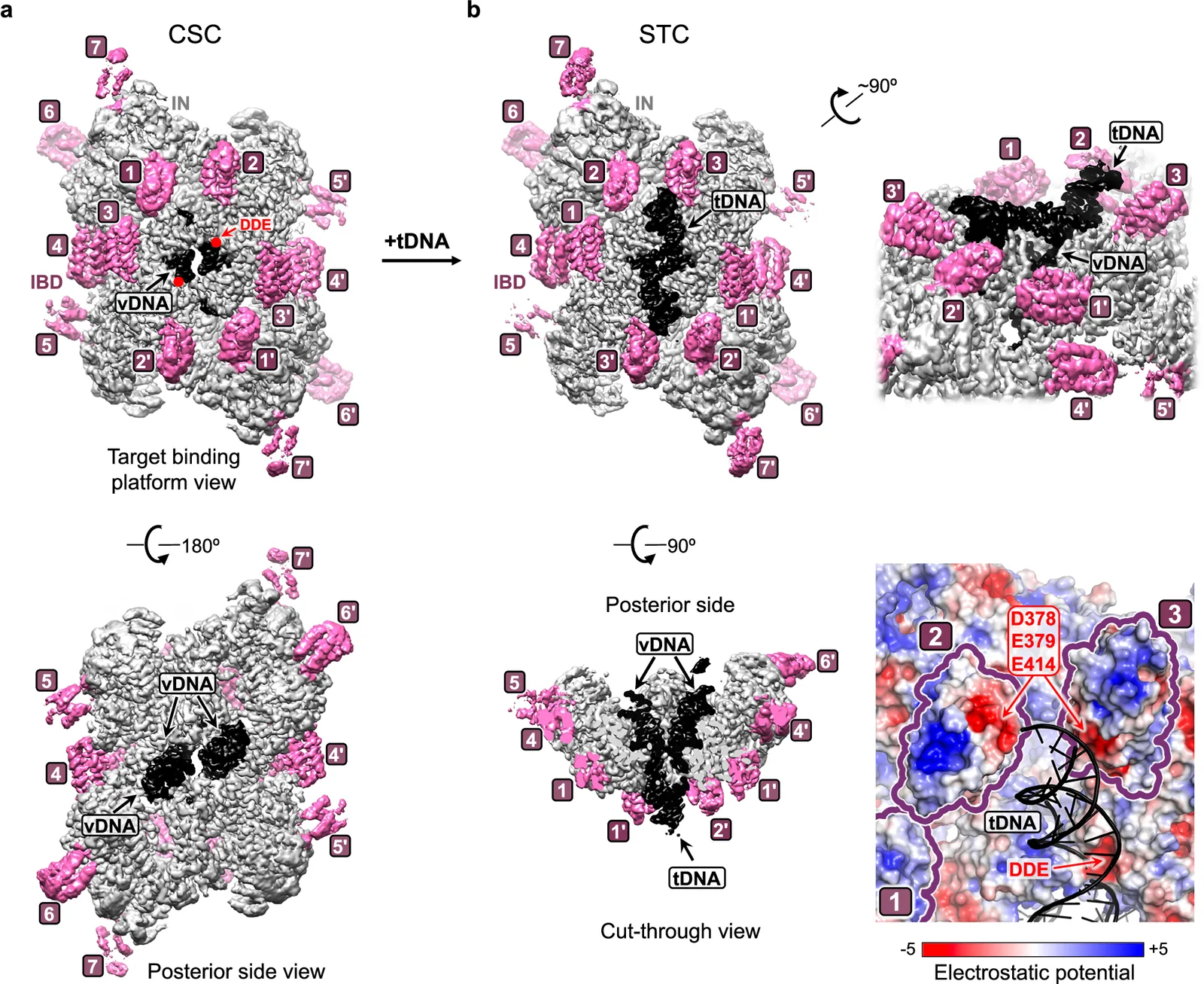
Cryo-EM reconstruction of MVV intasomes in complex with multiple copies of LEDGF/p75. Refined model of the MVV intasome cleaved synaptic complex (**a**, CSC) and strand transfer complex (**b**, STC) in complex with saturating quantities of LEDGF/p75. The CSC represents the intasome state after 3’-processing but prior to engaging target DNA (tDNA); the STC is the post-catalytic state of the intasome after covalent viral DNA (vDNA) joining to tDNA. The intasome consists of 16 IN molecules (white), of which 14 of the 16 potential LEDGF/p75 IN binding domains (IBDs, pink) are resolved. LEDGF/p75 molecules accrue on the tDNA side of the intasome, where subunits 1–3 and 1ʹ–3ʹ wall off the tDNA binding region to hinder local access to bulky chromatinized DNA. The map displays electrostatic potential (bottom right), where LEDGF/p75 IN-binding domains (IBDs, encircled in purple) present negatively charged patches in close proximity to tDNA.

We showed that LEDGF/p75 stimulates HIV-1 PICs and in vitro-assembled HIV-1 and MVV intasomes to integrate into H3K36Me3_N_-modified chromatin (Figs. 1d, e and 2a–c). The MVV intasome can be assembled as near monodispersed particles and, therefore, is particularly suitable for structural studies^8^. Although the intasome contains four IN tetramers and therefore is predicted to bind as many as 16 copies of LEDGF/p75, only two IBDs were captured in a recent cryo-EM structure^12^. LEDGF/p75 is a highly abundant protein, present in CD4^+^ T cells at an average intracellular concentration of 1–2 µM^82^. The bulk of this protein is concentrated in the nucleus, bound to chromatin^83^, where it potentially forms condensates^84,85^. Therefore, local concentration of LEDGF/p75 in the vicinity of chromatin, where HIV-1 PICs are released from their capsid cores^86^—is likely substantially higher. To mimic the nuclear environment during integration more closely, we imaged the MVV cleaved synaptic complex (CSC) and strand-transfer complex (STC) in the presence of 4 µM LEDGF/p75. To prevent aggregation, we immobilized the intasomes on functionalized graphene oxide surfaces and acquired cryo-EM images, which allowed us to refine structures of the CSC and the STC to overall resolutions of 2.8 Å (Fig. 5a, b, Supplementary Table 2). The local resolution reached 2.4 Å throughout the conserved intasomal core structure, including the functional IN active sites within the intasomes (Supplementary Figs. 7 and 8a, b). While the CSC and STC structures are highly similar, they represent separate functional states along the integration pathway, with the latter clarifying the tDNA-binding platform of the intasome.

As in earlier cryo-EM structures^8,12^, the IN component of the lentiviral intasome is comprised of 16 chains. The new reconstructions revealed that 14 of the 16 possible LEDGF/p75 IBD binding sites on the intasomes were at least partially occupied (Fig. 5a, b), and the final MVV CSC and STC intasome models were refined with 12 and 10 LEDGF/p75 IBD chains, respectively (Supplementary Fig. 8c). Although we used full-length LEDGF/p75 to assemble the complexes, only the IBDs were discernible in the cryo-EM maps, with the bulk of the host factor chains presumably disordered in the absence of chromatinized tDNA.

The lentiviral IN-LEDGF/p75 interaction was previously elucidated by partial crystal structures, which revealed that the tip of the IBD helical bundle latches onto the IN catalytic core domain (CCD) dimer interface and an electropositive face of IBD α4 interacts ionically with the IN N-terminal domain (NTD)^87–89^. The intasome exhibits 2-fold symmetry, allowing identification of eight distinct host factor binding locations (numbered 1–8, along with their symmetry equivalents 1’–8’). Despite uniform binding modes, substantial local disparity was noted among the IBD chains, evident from varying degrees of occupancy and local disorder (Fig. 5a, b). Indeed, while IBDs 1 and 1’ were previously observed^12^, IBDs 8 and 8’ are absent from both the current and past reconstructions. Herein, IBDs 1, 2, 3, 4, 6, along with their symmetry mates, were best defined (Fig. 5a, b), with the largest local root-mean-square deviation of 3.1 Å observed between Cα atoms of IBDs 4 and 6.

Strikingly, the IBDs exclusively decorate the tDNA-facing side of the intasome, collectively expanding its surface area by ~4000 Å² per IBD (Fig. 5a, b). The functional IN active sites within the intasome are turreted by six well-defined IBDs 1, 2, 3, 1’, 2’, and 3’. The STC structure revealed that IBDs—numbers 2, 3, 2’ and 3’—align along the tDNA binding platform of the intasome. However, tDNA deformation prevents clashes or encroachment into the space occupied by these IBDs. Such spatial arrangement prevents tDNA threading between the negatively charged patches presented by LEDGF/p75 IBD residues Asp-378, Glu-379, and Glu-414. The cryo-EM structures reveal that bound IBDs remodel the tDNA-binding platform of the intasome, acting as physical barriers to facilitate optimal linker DNA engagement for integration, while excluding the approach of more bulky core nucleosome particles.

## Discussion

Retroviral IN recognizes and acts upon vDNA ends, which are consumed following strand transfer and gap repair, making integration an irreversible process. The decision of where to offload its genome may be fateful for the virus: integration within inhospitable environments could lead to heterochromatinization and latency. Therefore, prior to this point of no return, the intasome must interpret local cues to assess the likelihood of proviral gene expression within a given genomic locus. Ostensibly, the mechanism would involve an active licensing function that could reject unsuitable locations while facilitating integration within appropriate domains. In the case of lentiviruses, the cue could be a local enrichment of nucleosomes containing H3K36Me3, recognized by multiple copies of LEDGF/p75 associated with the intasome.

How retroviral intasomes engage naked tDNA is well understood from structural studies, which revealed considerable local deformation in tDNA^2,12,19,90^, a property that is shared by prokaryotic transpososomes^91,92^. The deformation affords sufficient expansion of the major groove to allow widely spaced intasomal active sites to engage the scissile phosphodiester bonds in tDNA and, hypothetically, to store conformational strain to suppress the reversal of the transesterification reaction prior to gap repair^2^. In vivo, an estimated 80% of chromosomal DNA is present in the form of nucleosomes^63,93^, and the core nucleosome structure severely limits accessibility of the major groove due to wrapping around the histone octamer^51^. Although at select positions nucleosomal DNA exposes a widened major groove, the deformation is not sufficient for retroviral integration. Indeed, although the SHL ± 3.5 positions feature an outward-facing widened major groove (Supplementary Fig. 6c), the PFV intasome must lift the nucleosomal DNA from the histone octamer to impose a considerably sharper bend, assisted by sliding of the nucleosomal DNA arm proximal to the integration site^27,55^.

The specific parameters of tDNA deformation required for integration depend on the stagger used by retroviral intasomes to integrate both vDNA ends, which range from 4 bp for PFV to 5 or 6 bp for lentiviruses^56^. Whether the deformation sufficient for lentiviral integration can be tolerated by the nucleosomal core structure remains an open question. Although early studies observed that HIV-1 IN can target exposed major grooves on nucleosome core particles, these experiments relied on Mn^2+^ for catalysis and poorly defined IN-vDNA complexes solely capable of half-site strand transfer (i.e., single vDNA end integration, as opposed to concerted integration of synapsed vDNA ends promoted by intasomes)^52,53,94^. While the protagonist of Mn^2+^-dependent half-site strand-transfer activity has not been structurally characterized, it likely exhibits more relaxed constraints for tDNA engagement. Conversely, a more recent study reported that HIV-1 intasomes preferentially target chromatinized tDNA regions that are sparsely occupied by nucleosomes, indicating that stable nucleosome structures are disfavored targets for lentiviral integration^56^. We note that in vivo, most nucleosomes display transient positioning, especially within active transcription units^63^, and none of the prior studies directly mapped nucleosome positions along tDNA during or after integration.

The PFV and MVV intasomes used in this work have been structurally characterized at high resolution^3,12^ (Fig. 5). Although in vitro-assembled primate lentiviral intasomes suffer from considerable polydispersity, they are structurally analogous to the MVV intasome^9,10^. Using a strongly positioned chromatinized array, we showed that, in stark contrast to PFV, both lentiviral intasomes avoid integration into core nucleosomal regions (Fig. 3). However, representing a tandem repeat of a short DNA sequence, such arrays do not allow disentanglement of the effects of chromatin structure from those of nucleotide sequence on intasome activity. Indeed, the HIV-1 intasome displayed pronounced selectivity for a small subset of integration sites along our array (Fig. 3b, Supplementary Fig. 4). Conversely, although we reproduced the pronounced preference of the PFV intasome to integrate at SHL ± 3.5, only one of two such structurally equivalent positions was targeted on W601 nucleosomes, presumably because the second site did not present an optimal sequence for PFV integration (Fig. 3a); a similar asymmetry of integration into W601 nucleosomes was observed in our previous study^27^. Therefore, we sought to average out localized nucleotide effects in the backdrop of a complex mixture of chromatinized DNA targets. By assembling nucleosomes with a chemical mapping motif onto natural yeast DNA, we simultaneously mapped both nucleosome and integration site positions (Supplementary Fig. 4a). Using this approach, we confirmed PFV integration at SHL ± 3.5 positions and revealed a minor second site at SHL ± 5.5 that had not been previously characterized (Fig. 4b). By contrast, lentiviral intasomes largely integrated outside of the nucleosome core particle, within a relatively wide distance range from a nucleosomal dyad, starting from the flanking DNA, which is relatively loosely associated with the histone octamer, and into inter-nucleosomal linker DNA (Figs. 3, 4b). Importantly, irrespective of whether nucleosomes were assembled on highly defined W601 arrays or more diversely distributed on yeast DNA, lentiviral intasomes strongly disfavored the bodies of nucleosomes and instead targeted the linker DNA, while PFV integration was consistently observed within the nucleosome core.

Both qPCR and integration site sequencing supported a role for LEDGF/p75 to act as a tether for HIV-1 PIC for integration into H3K36Me3-modified chromatin (Figs. 1c, 2a, and 3d, e). The disordered region between the PWWP domain and the IBD in LEDGF/p75 comprises 255 amino acid residues, predicted to span an average distance of ~12 nm^95^ or ~90 nm when fully outstretched. Considering the linker DNA length of 10-20 nm^63^, the ~20-nm lentiviral intasome could plausibly engage several nucleosomes via multiple copies of LEDGF/p75. Concordantly, the host factor promoted HIV-1 integration within the central region of a H3K36Me3-modified polynucleosomal array, where the intasome could form most chromatin-reader interactions (Fig. 3f). Our cryo-EM structures revealed that while the PWWP domains presumably act at a distance from the body of the intasome, the IBDs reshape its tDNA binding platform (Fig. 5). We propose that multiple LEDGF/p75 molecules associated with the intasome function as an entropic filter by restricting the set of chromatin conformations and approach trajectories that can lead to productive engagement with the intasome active sites. In this model, access by bulky chromatin segments is generally obstructed. However, H3K36Me3-containing chromatin segments, entrapped through interactions with PWWP domains, can linger long enough to permit productive engagement. This model also explains why more flexible, nucleosome-free linker DNA segments can access the active sites in the presence of LEDGF/p75 (Figs. 3c, 4b). Conceptually, this mechanism resembles nuclear pore entry, where cargoes are licensed through productive interactions with nucleoporin FG repeats^96^. Thus, while PFV integrates into stable nucleosomes, often within deep heterochromatic regions^27,28^, lentiviruses recruit LEDGF/p75 to recognize multiple modified nucleosomes, facilitating integration into linker segments within the dynamic environment of a transcription unit. However, future studies will be required to test the entropic filter hypothesis. Understanding the rules of engagement between the intasome and chromatin, and how this impacts vDNA integration licensing mechanisms, will help to define the factors that mold the formation of the latent HIV-1 reservoir and pave the way to develop safer vectors for gene therapy applications.

## Methods

### Recombinant histones

The constructs for production of human histone variants were based on UniProt entries Q7L7L0 (H2A), Q16778 (H2B), Q71DI3 (H3.2), and P62805 (H4). H2A, H3(C110A), H3(C110A, K36C), H4, and H4(S47C), and the proteins were produced as inclusion bodies in *Escherichia coli*^97^. The mutation C110A, required for production of methyl-lysine analog (MLA) mimic of H3 K36Me3, was included in all H3 constructs for consistency. To facilitate inclusion body formation, H2B was produced with an N-terminal hexahistidine (His_6_) tag, which was cleaved with human rhinovirus 14 3C protease prior to assembly into histone dimers^27^. For the use in nucleosome mapping experiments, purified H4(S47C) was alkylated by incubation with N-(1,10 phenanthrolin-5-yl) iodoacetamide (Biotium, product code 92015) in 10 mM Tris, 1 mM EDTA, pH 8.0. Excess reagent was quenched with 5 mM β-mercaptoethanol, and the modified protein was dialyzed against 5 mM β-mercaptoethanol in the dark. Freeze-dried phenthroline-H4(S47C) conjugate was used for assembly in heterotetramers with H3 variants. Methyl-lysine analog (MLA) of tri-methylated Lys36 H3 was obtained by alkylation of H3.2(C110A, K36C) with 2-bromoethyltrimethylammonium bromide (Sigma Aldrich, product code 117196)^98^. The efficiency of the modifications was verified by electrospray ionization mass spectrometry.

### Semi-synthetic production of human histone H3(C110A) carrying tri-methylated Lys36

Native H3.2(C110A) carrying tri-methylated Lys36 was generated using a semi-synthetic approach based on reports by Ruthenburg and Fierz laboratories^68,99^. The procedure involved joining the synthetic H3(1-46) peptide, tri-methylated on Lys36, and a recombinant H3 construct spanning the remainder of the protein sequence and carrying an N-terminal Cys residue to allow protein ligation, H3.2(C110A, A47C)Δ(1–46). The recombinant construct, initially produced as a fusion with His_6_-SUMO, was isolated by immobilized metal affinity chromatography, and the tag was removed by digestion with Ulp1 protease. Following protein ligation, the full-length modified protein product was subjected to desulphurization to convert the artificially introduced Cys residues to the natural Ala residues. The experimental details are described in the following subsections.

#### The N-terminal portion of H3 spanning residues 1–46

The N-terminal portion was constructed from two synthetic portions: Fragment 1 (amino acid sequence: ARTKQTARKSTGGKAPRKQLATKA-NHNH_2_) and Fragment 2 (CRKSAPATGGV K^*^ KPHRYRPGTV-NHNH_2_; the N-terminal Ala residue on Fragment 2 was replaced with a Cys to allow native chemical ligation; asterisk indicates tri-methylated Lys). Both peptides were synthetized with a C-terminal hydrazide. Cl-Trt-resin (0.2 mmol; Merck) was pre-swelled in 4 mL 50:50 v/v dimethylformamide: dichloromethane for 30 min. The solvents were expelled, and the resin was loaded with 2 × 4 mL (2 × 30 min) 5% (v/v) hydrazine monohydrate in dimethylformamide to produce a hydrazide. The unreacted sites were blocked using 4 mL 5% (v/v) methanol in dimethylformamide for 10 min. The resin was washed in dimethylformamide three times, dried with 1:1 (v/v) dimethylformamide:dichloromethane. The peptides were produced using solid-phase peptide synthesis, on a ResPep automated peptide synthesizer (CEM) using 5 eq N(a)-Fmoc amino acids and 4.9 eq hexafluorophosphate azabenzotriazole tetramethyl uronium as the coupling reagent, and 10 eq 2 M diisopropylethylamine. The Fmoc deprotections were carried out on the synthesizer using 20% piperidine in dimethylformamide containing 1% formic acid. Standard Fmoc protected amino acids were used, along with the following additional amino acids: N(a)-Fmoc-Lys (Me3)-OH (Lys position 36), N(a)-Fmoc-Lys(^t^Boc)-Ser(ψ^Me,Me^pro)–OH, N(a)-Fmoc-Gly-Thr(ψ^Me,Me^pro)–OH, N(a)-Fmoc-Gln-Thr(ψ^Me,Me^pro)–OH, N(a)-Fmoc-Ala-Thr(ψ^Me,Me^pro)–OH and N(a)-Fmoc-Gly-(Dmb)Gly–OH. All amino acids were double-coupled.

Following assembly, both peptides were cleaved from the resin and protecting groups removed by addition of 10 mL cleavage solution comprising 95% trifluoracetic acid (TFA), 2.5% water, 2.5% triisopropylsilane for Fragment 1, and 94% TFA, 2.5% water, 2% triisopropylsilane, and 2% ethanedithiol for Fragment 2. After 4 h, the resin was removed by filtration, and the peptides were precipitated with 40 mL ice-cold diethyl ether. The peptides, isolated by centrifugation, were dissolved in 10 mL water and freeze-dried overnight. Following lyophilization, the peptides were purified in 40 mg batches on a C8 reverse phase HPLC column (Agilent PrepHT Zorbax 300SB-C8, 21.2 × 250 mm, 7 m) using a linear 0–20% B gradient developed over 40 min, at a flow rate of 8 mL/min; the standard binary solvent system was used comprising aqueous 0.08% TFA/1% acetonitrile as solvent A and 0.08% TFA in acetonitrile as solvent B. The peak fractions were analyzed by liquid chromatography coupled with mass spectrometry (LC–MS) on an Agilent 1290 LC/MSD system (Agilent Poroshell 120 EC-C18 2.7 μm, 3.0 × 30 mm column), using a linear gradient of 5–60% B over 12.5 min at a flow rate of 0.425 mL/min, using the standard binary solvent system (described above). The calculated molecular weights (MWs) of the peptides agreed with the observed MWs for the products: for Fragment 1, the expected and measured MWs were 2569.49 and 2568.79, respectively; for Fragment 2, 2422.29 and 2422.27.

The two peptides were subsequently ligated at a 1:1 molar ratio following published procedures^100^. Fragment 1 (32 mg) dissolved in 0.5 mL 6 M guanidine hydrochloride/0.2 M aqueous phosphate buffer, pH 3–3.1 at −15 °C for 15 min was supplemented with 50 µL 0.5 M NaNO_2_ to oxidize the peptide hydrazide to an azide. The reaction was allowed to proceed for 15 min at −15 °C. Next, Fragment 2 (30 mg) and 100 eq of 4-mercaptophenyl acetic acid in 6 M guanidine hydrochloride/0.2 M aqueous phosphate buffer, pH 6.5, was added to the reaction mixture containing Fragment 1. The pH of the solution was adjusted to pH 6.8–7 and left to stir overnight. The reaction was monitored using LC–MS, and after completion was supplemented with 500 µL 0.1 M tris(2-carboxyethyl) phosphine (TCEP) in 6 M guanidine hydrochloride/aqueous 0.2 M phosphate buffer, pH 7. The reaction mixture was directly purified on a C8 reverse phase HPLC column (Agilent PrepHT Zorbax 300SB-C8, 21.2 × 250 mm, 7 m) using a linear solvent gradient of 0–30% B over 40 min at a flow rate of 8 mL/min, using the standard binary solvent system (described above). The peak fraction was analyzed by LC–MS on an Agilent 1290 LC/MSD system as above. The calculated MW of the ligated peptide product (amino acid sequence: ARTKQTARKSTGGKAPRKQLATKACRKSAPATGGVK^*^KP HRYRPGTV-NHNH_2_; the asterisk indicates tri-methylated Lys), 4958.77, agreed closely with the MW measured by LC–MS, 4957.77; the final yield was 24.5 mg.

#### The C-terminal portion of H3 spanning residues 47–135

Recombinant His_6_-SUMO-tagged H3.2(C110A, A47C)Δ(1–46) was digested with Ulp1 protease (20 µg protease per 1 mg SUMO-tagged protein) during dialysis against 1 M urea supplemented with 25 mM L-cysteine and 25 mM L-arginine. Cleaved protein, solubilized in 1% acetic acid, was further purified on a C8 reverse phase HPLC column (Agilent PrepHT Zorbax 300SB-C8, 21.2 × 250 mm, 7 m) using a linear solvent gradient of 0–65% B over 40 min at a flow rate of 8 mL/min, using the standard binary solvent system (described above). The peak fraction was analyzed by LC–MS on the Agilent 1290 LC/MSD system as above. The calculated molecular weight 10,402.67 of the polypeptide (amino acid sequence: CLREIRRYQKSTELLIRKLPFQRLVREIAQDFKTDLRFQSSAVMALQEASEAYLVGLFEDTNLAAIHAKRVTIMPKDIQLARRIRGERA) agreed well with the mass measured, 10,401.87.

#### Assembly and desulfurization of the final product

The two fragments (H3 residues 1–46 and 47–135) were ligated at a 1.2:1 molar ratio (24.5 and 44.4 mg, respectively). The N-terminal fragment dissolved in 0.5 mL 6 M guanidine hydrochloride/0.2 M aqueous phosphate buffer, pH 3–3.1 at −15 °C for 15 min was supplemented with 50 µL 0.5 M NaNO_2_ in water to convert the C-terminal hydrazide to azide. The mixture was stirred at −15 °C for 15 min and combined with the recombinant C-terminal H3 fragment and 100 eq 4-mercaptophenyl acetic acid in 6 M guanidine hydrochloride/0.2 M aqueous phosphate buffer, pH 6.5; pH was adjusted to 6.8–7 and the mixture was left to stir overnight. Reaction progress was monitored by LC–MS, and after completion, the mixture was supplemented with 500 µL 0.1 M TCEP in 6 M guanidine hydrochloride/0.2 M aqueous phosphate buffer, pH 7. The reaction mixture was directly purified on a C8 reverse phase HPLC column (Agilent PrepHT Zorbax 300SB-C8, 21.2 × 250 mm, 7 m) using a linear solvent gradient of 0–60% B over 40 min at a flow rate of 8 mL/min, using the standard binary solvent system (described above). The peak fraction was analyzed by LC–MS on the Agilent 1290 LC/MSD system as above. The calculated MW of the ligated peptide (amino acid sequence: ARTKQTARKSTGGKAPRKQLATKACRKSAPATGGVK^*^KPHRYRPGTVCLREIRRYQKSTELLIRKLPFQRLVREIAQDFKTDLRFQSSAVMALQEASEAYLVGLFEDTNLAAIHAKRVTIMPKDIQLARRI RGERA; Cys residues marking the sites of ligation are underlined; the asterisk indicates tri-methylated Lys36), 15,328.44, agreed closely with the measured MW of the product, 15,328.48.

The ligated polypeptide then underwent desulphurization, to convert the Cys residues to Ala. 1 mL 6 M guanidine hydrochloride/0.2 M aqueous phosphate buffer, pH 7, was supplemented with 0.5 M TCEP and 0.1 M radical initiator VA-044 (2,2′-azobis[2-(2-imidazolin-2-yl) propane] dihydrochloride) and then with 10% (v/v) 2-methyl-2-propanethiol. Following degassing, 0.5 mL of the reagent mixture was added to 15 mg polypeptide and stirred overnight at 37 °C. The reaction was monitored using LC–MS. Upon completion, desulphurized product was purified on a C8 reverse phase HPLC column (Agilent PrepHT Zorbax 300SB-C8, 21.2 × 250 mm, 7 m) using a linear solvent gradient of 0–60% B over 40 min at a flow rate of 8 mL/min, using the standard binary solvent system (described above). The peak fraction was analyzed by LC–MS on an Agilent 1290 LC/MSD system (Agilent Poroshell 120 EC-C18 2.7 µm, 3.0 × 30 mm column), using a linear gradient of 5–100% B over 12.5 min at a flow rate of 0.425 mL/min. The same binary solvent system was used. The calculated MW of the final product (amino acid sequence: ARTKQTARKSTGGKAPRKQLATKAARKSAPATGGVK^*^KPHRYRPGTVALREIRRYQKSTELLIRKLPFQRLVREIAQDFKTDLRFQSSAVMALQEASEAYLVGLFEDTNLAAIHAKRVTIMPKDIQLARRIRGERA; the asterisk indicates tri-methylated Lys36), 15,266.44, agreed with the MW determined, 15,263.63 (Supplementary Fig. 1b).

### Nucleosomal arrays

Freeze-dried histones were resuspended in unfolding buffer (6 M guanidinium hydrochloride, 1 mM EDTA, 10 mM DTT, 20 mM Tris–HCl, pH 7.5). WT and modified H2A/H2B heterodimers and H3/H4 heterotetramers were assembled by dialysis of the corresponding histone mixtures (prepared at a 1:1 molar ratio and a total protein concentration of 1 mg/mL) against refolding buffer (2 M NaCl, 5 mM β-mercaptoethanol, 1 mM EDTA, 20 mM Tris–HCl, pH 7.5). The resulting complexes were purified by size-exclusion chromatography through a Superdex 200 10/300 column equilibrated in refolding buffer^97^. DNA for regular polynucleosomal arrays contained 12 repeats of the Widom 601 sequence^62^ separated by 44-bp linkers (Supplementary Fig. 1a), which was produced by EcoRI cleavage of 1–3 mg pcDNA3-12xW601 plasmid and purified by agarose gel electrophoresis using Maxi gel extraction kit (Macherey-Nagel, product code 740610.20). Yeast genomic DNA, isolated from *Saccharomyces cerevisiae* strain BY4741 (ref. ^101^) using YeaStar Genomic DNA kit (Zymo Research, product code D2002), was sheared by digestion with restriction enzymes XbaI and NheI (New England Biolabs) in the presence of 0.1 mg/ml RNase A (Qiagen) to generate DNA fragments with a median size of ~2 kb. The DNA was deproteinized by organic extraction and ethanol precipitation. Polynucleosomal arrays were assembled by salt-gradient dialysis from 2 M NaCl to 0.1 M NaCl in 10 mM Tris–HCl, pH 7.5, 0.5 mM DTT^97^. The DNA:tetramer:dimer molar ratio was kept at 1:12:24, and nucleosomes were heat repositioned for 30 min at 37 °C. Chromatin assembled on the W601 array or yeast genomic DNA was validated by digestion with EcoRV, which cuts in the middle of the 44-bp linker, or micrococcal nuclease, respectively. The products were separated by native electrophoresis in 0.5x Tris–borate–EDTA (TBE) buffer on 4–20% polyacrylamide gels (ThermoFisher Scientific); DNA was detected by staining in 10 µg/ml ethidium bromide in TBE supplemented with 0.2% sodium dodecylsulfate (SDS).

### Atomic force microscopy analysis

We prepared natural chromatin samples with phenthroline-H4(S47C) conjugate, with (“Me3”) and without (“Me0”) modification, by diluting to a final concentration of 1 ng/μL in low salt buffer comprising 5 mM NaCl and 10 mM Tris–HCl, pH 7.5. This sample was left on ice for 10 min prior to sample deposition on poly-l-lysine-coated mica. Freshly cleaved muscovite mica (SPI Supplies) was treated by drop-casting 20 μl poly-L-lysine (Merck; 0.01% w/v in sterile milliQ water) for 30 s and subsequently rinsing the surface with 20 ml milliQ water before drying with a gentle stream of filtered N_2_ gas^102^. The sample (20 μL) was then deposited by dropcasting on the poly-L-lysine-coated muscovite mica for 15 s and rinsed with 20 ml milliQ water before drying in a gentle stream of filtered N_2_ gas.

A Nanowizard 4 XP AFM (JPK Instruments) was used in tapping mode with silicon tips (FASTSCAN-A; drive frequency, 1400 kHz; Bruker) over fields of view of 6 × 6 μm at 4096 × 4096 pixels and captured at line rates of 3 Hz. To process the raw topographic data, we used SPIP software (v6.1.1, Image Metrology) for plane correction, including plane-fitting with a third-degree polynomial, and line-by-line correction with a fourth-degree polynomial.

### Preparation of HIV-1 PICs

HEK-293T or PSIP1/HDGF2-double knockout HEK-293T (LHKO) cells^12^ were seeded in six-well dishes (10^6^ cells per well) and grown at 37 °C overnight in Dulbecco’s modified Eagle medium (DMEM; Life Technologies) supplemented with 10% (v/v) fetal calf serum (FCS) (Life Technologies). The next day, cells were infected with an HIV-1-derived lentiviral vector encoding GFP reporter^103^ at a multiplicity of infection (MOI) of 30 in 2 mL DMEM containing 10% (v/v) FCS, supplemented with 7.5 µg/mL polybrene (Sigma-Aldrich) by spinoculation at 1500×*g* for 1 h at 16 °C, and returned to the 37 °C CO_2_ incubator for 24 h. To prepare PICs^104^, infected cells, harvested by trypsinization were washed in three changes of buffer K^−/−^ (150 mM KCl, 5 mM MgCl_2_, 20 mM 4-(2-hydroxyethyl)-1-piperazineethanesulfonic acid (HEPES)-NaOH, pH 7.6) and lysed in ice-cold buffer K^+/+^ (buffer K^−/−^ supplemented with 1 mM 1,4-dithiothreitol (DTT), cOmplete EDTA-free protease inhibitor cocktail (Roche) and 0.025% (w/v) digitonin) for 10 min at room temperature with gentle mixing. Nuclei, pelleted by centrifugation at 1500×*g* for 4 min at 4 °C, were resuspended in 1 mL ice-cold extraction buffer (300 mM KCl, 5 mM MgCl_2_, 25% glycerol, 1 mM DTT, 20 mM HEPES-NaOH, pH 7.6, cOmplete EDTA free protease inhibitors (Roche)). To facilitate PIC extraction, the suspension was sonicated on ice using a 3-mm needle tip (Branson 450) at 25 W for a total of 4 s over 40 s. Insoluble material was cleared by centrifugation at 19,000×*g* for 15 min at 4 °C. The supernatant was collected as the crude nuclear PIC extract and used immediately.

### Assembly of retroviral intasomes

Retroviral intasomes were assembled using vDNAs produced by annealing the pairs of oligonucleotides listed in subsequent sections. While the PFV intasomes were assembled by salt dialysis of IN/vDNA mixtures^3^, HIV-1 and MVV intasome assembly using WT INs required the presence of LEDGF/p75^8,9,44^ as described below. The transferred strands of the vDNA constructs were designed with 5’-extensions to allow annealing of the primer and a common TaqMan probe for strand-transfer product quantification by qPCR, as well as ligation of Nanopore sequencing adapters. Modified vDNA oligonucleotides were used in intasome assembly for nucleosome/integration site co-mapping experiments as described below. To prepare PFV intasomes, a mixture containing 120 μM PFV IN and 50 μM vDNA duplex prepared in 500 mM NaCl, 50 mM BisTris propane–HCl, pH 7.45 was dialyzed against excess 200 mM NaCl, 2 mM DTT, 25 μM ZnCl_2_, 20 mM BisTris propane–HCl, pH 7.45 overnight at 18 °C. The PFV intasomes were solubilized by adjusting NaCl concentration to 0.32 M on ice. The MVV and HIV-1 intasomes were assembled by incubating 7 μM IN, 8 μM LEDGF/p75, and 3.75 μM annealed vDNA in 80 mM NaCl, 40 mM potassium acetate, 3 mM CaCl_2_, 10 μM ZnCl_2_, 1 mM DTT, and 25 mM BisTris–HCl, pH 6.0, in a total volume of 200 μl at 37 °C for 10 min. The opalescent mixture was supplemented with 50 mM BisTris–HCl, pH 6.5, and an additional 190 mM NaCl and incubated on ice for 5 min to clear. Where stated, the intasomes were purified by size-exclusion chromatography through Superdex 200 increase 10/300 column (GE Healthcare) in 310 mM NaCl, 25 mM BisTris–HCl pH 6.5 (MVV), or 320 mM NaCl, 25 mM BisTris propane–HCl pH 7.45 (PFV). HIV-1 intasomes were purified by size exclusion chromatography through a TSKgel UltraSW Aggregate HPLC column (TOSOH Biosciences, product code 0022856) in 350 mM NaCl, 25 mM BisTris propane–HCl, pH 6.0. Intasomes containing *Sulfolobus solfataricus* Sso7d - HIV-1 IN chimera^69^ were formed as described above for HIV-1 intasomes, except that LEDGF/p75 was omitted.

### Strand-transfer activity assays

Extracted HIV-1 PICs (10 µL) were incubated with 10 µL poly-nucleosome arrays or naked DNA (1.5 ng/µL) in tDNA dilution buffer (20 mM HEPES–NaOH, pH 7.6, 5 mM MgCl_2_) for 20 min at 37 °C. Reactions were stopped by heating at 95 °C for 5 min, and the products were treated with 20 µg proteinase K at 60 °C for 30 min, and subsequently denatured at 95 °C for 15 min. Intasomes assembled with annealed pairs of oligonucleotides EV333 (5’-ACTGCTAGAGATTTTCCACACTGACTAAAAGGGTCTGAGG)/EV346 (5’-GTGTGTGCCCGTCTGTTGTGTGACTCTGGTAACTAGAGATCCCTCAGACCCTTTTAGTCAGTGTGGAAAATCTCTAGCA; HIV-1), EV244 (5’-GCTGCGAGATCCGCTCCGGTG)/EV361 (5’-GTGTGTGCCCGTCTGTTGTGTGACTCTGGTAACTAGAGATCCCTCAGACCCTTTTAGTCAGCACCGGAGCGGATCTCGCA; MVV) were diluted 10,000-fold in intasome dilution buffer (20 mM HEPES–NaOH, pH 8.0, 300 mM KCl, 1 mM MgCl_2_, 5 µM ZnCl_2_, 1 mM DTT, 20% (v/v) glycerol). Ten µl diluted intasome was incubated with 10 µL poly-nucleosome arrays or naked DNA (1.5 ng/µL) in intasome reaction buffer (20 mM HEPES–NaOH, pH 8.0, 1 mM MgCl_2,_ 1 mM DTT, 20% glycerol, 5 µM ZnCl_2_). Strand transfer reactions proceeded and were processed as described for PIC-based assays. Deproteinized strand-transfer products were quantified by qPCR using primers PC1674 (5’- GTGTGTGCCCGTCTGTTGTG), JH4 (5’-CCGAGATATCAAGCGACAC), and probe PC1675 (FAM-CTGGTAACTAGAGATCCCTCAGACCCTT TTAGTCAG-TAMRA)^105^ and TaqMan Fast Advanced Master Mix (Applied Biosystems, product code 4444557) in a final reaction volume of 13 µl. qPCR reactions were performed using a Viia7 or Quant studio 7 Flex instrument (Applied Biosystems) with the following cycling parameters: 95 °C for 20 s followed by 40 cycles of 95 °C for 1 s and 60 °C for 20 s with ramp rate set to 2.42 °C/s. The cycle threshold (Ct), defined as the cycle at which fluorescence exceeded background, was determined for each well using QuantStudio Real-Time PCR software v1.2 (Applied Biosystems). A 7-point standard curve was generated using 5-fold serial dilutions of a cloned integration junction, starting with 3 million copies per reaction. For each condition, qPCR reactions were performed in technical triplicate.

### Mapping integration sites into the W601 polynucleosomal array

Intasomes were assembled with annealed vDNA oligonucleotides EV333/[phos]-EV346 (5’-PO_4_-GTGTGTGCCCGTCTGTTGTGTGACTCTGGTAACTAGAGATCCCTCAGACCCTTTTAGTCAGTGTGGAAAATCTCTAGCA, 5’-phosphorylated; HIV-1) or EV352 (5’-ATTGTCATGGAATTTCGC GA)/[phos]-EV351 (5’-PO_4_-CGCGAAATTCCATGACA, 5’-phosphorylated; PFV) and purified by size exclusion chromatography as stated in the section on assembly of retroviral intasomes. Purified intasomes (0.5 nM in intasome dilution buffer, 200 µL) and LEDGF/p75 (4× indicated final concentration, 200 µL) in intasome dilution buffer were mixed and incubated for 10 min on ice, then supplemented with array or naked tDNA (1.5 ng/µL in intasome reaction buffer, 400 µL). Strand transfer was allowed to proceed for 20 min at 37 °C. The reactions were stopped by the addition of 0.25% SDS, 12.5 mM EDTA, 25 mM Tris–HCl pH 7.5, 320 µg proteinase K, and incubated at 50 °C for 1 h. In the case of HIV-1, DNA oligonucleotide EV372 was added at a 40-fold molar excess of vDNA and annealed at 72 °C for 10 min. DNA products, deproteinized by organic extraction and ethanol precipitation, were analyzed using Oxford Nanopore Technologies’ long read sequencing with the Native Barcoding kit (Oxford Nanopore Technologies, product code SQK-NBD114.24) and the adapter ligated to the vDNA end. To facilitate sequencing, the length of the DNA fragments was increased by ligation of an unrelated 400-bp DNA fragment to the tDNA ends at the same time as the barcode ligation step. Nanopore reads were preprocessed by aligning reads to each reference sequence (polynucleosomal array, pcDNA3 backbone, phiX174) with minimap2 version 2.28 (ref. ^106^), sequence IDs extracted using SAMtools version 1.20 (ref. ^107^), and filtered using SeqKit version 2.7.0 (ref. ^108^). For local mapping of integration sites to a single nucleosome, the sequence immediately downstream of the vDNA was extracted and mapped to a single repeat of the polynucleosomal array DNA on both the forward and reverse strands. For mapping of integration sites across the entire array, the number of nucleotides between the vDNA integration site and the ligation site of the 400-bp fragment was determined.

### Co-mapping integration sites and nucleosomal dyads on chromatinized yeast genomic DNA

To facilitate specific recovery of stand transfer products by LM-PCR and suppress amplification of vDNA-linker ligation products, HIV-1 and MVV intasomes were assembled using vDNA with 1’,2’-dideoxyribose (abasic dSpacer modification; Sigma Aldrich) appended to the 5’ ends of the non-transferred strands. Thus, vDNAs were produced by annealing oligonucleotides EV390 (5’-dSpacer-ACTGCTAGAGATTTTCCACACTGACTAAAAGGGTCTGAGG)/EV346 (to assemble HIV-1 intasome), EV402 (5’-dSpacer-GCTGCGAGATCCGCTCCGGTGCTGACTAAAAGGG)/EV361 (MVV), or EV373 (5’-TGTGACTCTGGTAACTAGAGATCCCTCCGAAATTCCATGACA)/EV374 (5’-ATTGTCATGGAATT TCGGAG; PFV). The intasomes, assembled and purified by size-exclusion chromatography. Yeast genomic DNA was chromatinized using H4(S47C)-phenanthroline conjugate with or without H3K36Me3 modification, as described above. All incubations with phenanthroline-modified chromatin were conducted in the dark. Strand transfer reactions were initiated by combining 200 µL intasome (0.5 nM in 300 mM NaCl, 1 mM MgCl_2_, 5 µM ZnCl_2_, 20% glycerol, 20 mM HEPES–NaOH pH 8.0, diluted immediately prior to the experiment) with an equal volume of chromatinized yeast genomic DNA (3 ng/µL by DNA content in 1 mM MgCl_2_, 5 µM ZnCl_2_, 20% glycerol, 20 mM HEPES–NaOH pH 8.0). Where stated, LEDGF/p75 was included at the final concentration of 8 nM with the rest of the reaction parameters unchanged. Strand transfer was allowed to proceed for 20 min at 37 °C. The samples were then diluted with an equal volume of 100 mM Tris–HCl, pH 7.5, to reduce NaCl concentration^109^ and sequentially supplemented with 0.1 µM CuCl_2_, 6 mM 3-mercaptopropionic acid, and 6 mM hydrogen peroxide. DNA fragmentation was allowed to proceed for 20 min at room temperature, and the reaction was quenched by the addition of 2.8 mM Cu ion chelating agent neocuproine. DNA products were deproteinized by digestion with proteinase K (~0.35 mg/ml) in the presence of 12.5 mM EDTA and 0.25% SDS for 1 h at 50 °C, followed by organic extraction. Deproteinized DNA, supplemented with 0.3 M sodium acetate, pH 5.2, and 20 µg glycogen (Life Technologies), was precipitated with isopropanol (41% v/v final concentration), re-dissolved in 20 µL water, and processed for LM-PCR.

To suppress direct linker joining to unreacted vDNA ends and NheI/XbaI-digested genomic DNA, deproteinized DNA fragments were blocked by ligation with optimized end blockers. The NheI/XbaI end blocker was obtained by annealing oligonucleotides EV381 (5’-CTCCGCTTAAGGGAC) and EV384 (5’-PO_4_-CTAGGTCCCTTAAGCGGAG-NH_2_, modified with 5’ phosphate and 3’ amino groups). PFV, HIV-1, and MVV vDNA end blockers were made by annealing pairs of oligonucleotides EV381 and EV382 (5’-PO_4_- ATGTCCCTTAAGCGGAG-NH_2_), EV381 and EV386 (5’-PO_4_-GTGTCCCTTAAGCGGAG-NH_2_), EV381 and EV404 (5’-PO_4_-GCGTCCCTTAAGCGGAG-NH_2_), respectively. Ligation reactions were conducted in a total volume of 25 µL using 4 µL deproteinized fragmented strand transfer DNA products, 1.28 µM NheI/XbaI end blocker, and 0.8 µM specific vDNA end blocker using NEBNext quick ligation module (New England Biolabs) for 30 min at room temperature, followed by overnight incubation at 16 °C. Blocked DNA fragments were isolated using 3 volumes of Ampure XP magnetic beads (Beckman).

DNA products were repaired by incubation with phage phi29 DNA polymerase (ThermoFisher Scientific, product code 10233130) and 0.4 mM dNTPs in a final volume of 50 µL. The polymerase was inactivated by 30-min incubation at 65 °C, and DNA fragments were 3’ dA-tailed using the Ultra II kit (New England Biolabs, product code E7546S) in the final volume of 60 µL, followed by enzyme inactivation at 65 °C for 30 min. End-prepped DNA samples (25 µL) were ligated to the linker DNA (obtained by annealing oligonucleotides EV375 and EV376) using Blunt/TA Ligase Master Mix (New England Biolabs, product code M0367S) in a final volume of 52 µL. The DNA products were purified using 3 volumes of AmpureXP beads and PCR amplified using AmpliTaq Gold Fast PCR Master Mix (ThermoFisher Scientific, product code 4390939) and primers EV377 and EV378. The PCR products were sequenced using Oxford Nanopore technology (FullCircle Labs, Imperial College London, UK).

Nanopore reads containing the vDNA end (5’-CTCTAGCA, 5’-ATCTCGCA, or 5’-CCATGACA for HIV-1, MVV, and PFV, respectively; only a precise match was considered) and the linker (5’-TAAGGGACT; allowing one mismatch) sequence tags in converging orientations, with at least 15 bp of intervening sequence, were cropped and brought into sense orientation with respect to vDNA. Cropped sequences were aligned to the yeast SacSer3 assembly (https://genome.ucsc.edu/cgi-bin/hgTracks?db=sacCer3) using BLAT version 3.5 (https://genome-test.gi.ucsc.edu/~kent/src/) with the following options: stepSize 5, repMatch 2253, minScore 20, minIdentity 0. Only matches that started immediately at the vDNA and linker junctions with overall < 12% mismatches (Q gap bases + T gap bases + mismatch)/match < 0.12, were used for further analysis. Those with non-unique combinations of genomic positions of integration (T start) and linker ligation sites (T end) were considered PCR duplicates and counted only once. To avoid reads originating from linker ligation to ends of yeast genomic DNA fragments, all matches within 10 bp of an XbaI or NheI site were removed. The histograms in Fig. 4b show the distribution of genomic distances (<150 bp) between mapped integration and linker ligation sites. Inclusion of distances >150 bp did not reveal additional peaks but instead produced extended tails, consistent with a lack of long-range interactions. BedTools version 2.30.0 (ref. ^110^) fastaFromBed was used to extract sequences of genomic fragments. Comparisons of integration site distributions were performed using *χ*² tests, with the corresponding effect sizes reported as Cramér’s *V* with 95% confidence intervals, calculated in R version 4.6.1 (https://cran.r-project.org/) using the chisq.test() function and the cramers_v() function from the effectsize package.

### Cryo-EM sample preparation, data collection, and image processing

To overcome excessive aggregation, we immobilized MVV intasomes on dibenzocyclooctyne (DBCO)-functionalized cryo-EM grids prior to saturation with LEDGF/p75. To this end, the intasomes were assembled using DNA constructs carrying azido groups: the MVV CSC complex was formed using a double-stranded oligonucleotide mimicking the terminal 29 bp of the processed MVV U5 vDNA end prepared by annealing synthetic oligonucleotides EV272 (5′-CCGTGCAACACCGGAGCGGATCTCGCA) and EV273-N3 (5′- GCTGCGAGATCCGCTCCGGTGTTGCACGG, with 3’-azide NHS ester modification). The MVV STC was assembled using a DNA construct corresponding to the product of full-site integration of 23-bp MVV U5 viral DNA end (vDNA) mimic into a palindromic 52-bp target DNA. The branched DNA was made by annealing oligonucleotides EV391 (5’- CCGTGCAACACCGGAGCGGATCTCGCA**GTCGAC**CACCCTAATCAAGTCACTTAAGCGGATACGGG, where the nucleotides contributing to the stagger are bold print), EV392 (5’-CCCGTATCCGCTTAAGTGACTTGATTAGGGTG, and EP273-N3. The HPLC-purified oligonucleotides were purchased from Integrated DNA Technologies. Cryo-EM grids functionalized with PEG-DBCO were prepared according to Wang et al.^111^. Briefly, Quantifoil Au 300-mesh 1.2/1.3 grids (Agar Scientific) were overlayed with large flake graphene oxide powder (William Blythe, product code AA02-182P) using surface assembly^112^. The coated grids were incubated with 5 mM amino-PEG4-DBCO (Click Chemistry Tools, product code A103P) in dimethyl sulfoxide for 16 h and washed with several changes of dimethyl sulfoxide. Purified MVV CSC or STC intasomes (10 μl in 500 mM NaCl, 20 mM BisTris–HCl, pH 6.5, 3 mM CaCl_2_) were applied onto the functionalized grids for 15 min on ice. Unbound material was washed off with 150 mM NaCl, 3 mM CaCl_2_, 25 mM BisTris–HCl, pH 6.5, and the intasomes, covalently attached to the surface, were soaked in a 10-μl drop of 0.25 mg/mL LEDGF/p75 on ice. After 10-min incubation at room temperature, the grids were blotted and plunged into a liquid propane–ethane mixture using Vitrobot Mark IV (ThermoFisher Scientific).

Initial MVV CSC dataset (4463 micrograph movies) was acquired on a Talos Arctica transmission electron microscope (ThermoFisher Scientific) operating at 200 keV and equipped with a Falcon 3 direct electron detector (ThermoFisher Scientific). Data collection was performed using EPU software (ThermoFisher Scientific) at a magnification of ×96,000, corresponding to a pixel size of 1.59 Å, a total exposure of 71 e/Å^2^ spread over 10 frames, and a defocus range of −1.5 to −3.5 μm. The frame stacks were aligned and combined using dose weighting with MotionCor2 version 1.4.0^113^, and contrast transfer function (CTF) parameters were estimated from frame sums using Gctf version 1.18^114^. Particles, picked using crYOLO version 1.9.9 (ref. ^115^) with the general model, were subjected to 2D classification in Relion-4.0 (Supplementary Fig. 9a, b)^116,117^. Particles belonging to well-defined 2D classes were subjected to 3D classification in Relion-4.0 using the volume from EMD_14860 (ref. ^8^), low-pass filtered to 60 Å resolution, as an initial reference, without imposing symmetry. Particles belonging to the intasome class were used to train Topaz^118^ for picking the entire dataset. Following several rounds of 2D classification and 3D classifications in Relion-4.0, the remaining subset of 51,626 particles was classified by Ab-initio reconstruction in cryoSPARC version 4.1.0 (ref. ^119^) into three classes. The resulting 21,996 particles, contributing to a well-defined intasome 3D class, were used for non-uniform refinement in cryoSPARC, imposing C2 symmetry. The resulting reconstruction, refined to ~7 Å resolution, displayed clear features corresponding to bound IBDs (Supplementary Fig. 9c). This volume was used to generate initial reference maps for heterogenous refinements during image processing of the Krios datasets (Supplementary Fig. 9c).

For high-resolution reconstructions of MVV intasomes, the particles were imaged on a Titan Krios G2 transmission electron microscope with a Falcon 4i detector and a Selectris energy filter (ThermoFischer Scientific). Micrograph frame stacks were recorded at a calibrated magnification corresponding to 0.95 Å per physical pixel and accumulated exposure of 41 e/Å^2^ over 1674 EER frames per micrograph movie. A total of 33,853 (CSC) and 27,316 (STC) micrograph movies were acquired using an energy filter slit width of 7 eV and a defocus range of −1.5 to −3.3 µm. The EER frames were combined into 31 fractions, aligned, and summed with dose weighting, as implemented in Relion-4.0, and CTF parameters were estimated using Gctf version 1.18. The sets of ~7.6 million CSC and ~4.0 million STC particles picked using Topaz, extracted with a pixel size of 3.8 Å were subjected to a single round of reference-free 2D classification in cryoSPARC version 4.4.1 over 80 iterations with 200 classes and a batch size of 800 particles per class. Only particles belonging to 2D classes corresponding to graphene oxide edges, ice, or obvious noise were discarded. The remaining particles were subjected to two (CSC) or three (STC) rounds of heterogenous refinement in cryoSPARC with the initial model generated from the Talos CSC reconstruction and 5 copies of a junk trap, prepared from the same map by phase randomization of all Fourier components exceeding the spatial frequency 1/125 Å (Supplementary Fig. 9d). No symmetry was imposed at this stage, and the input maps were low-pass filtered to 20 Å. At the end of each round of heterogenous refinement, the 3D class corresponding to non-phase randomized input map reached a resolution of 7.6 Å, corresponding to the Nyquist limit at the binning level (Supplementary Fig. 9d). The procedure yielded the subsets of 937,436 and 314,232 CSC and STC particles, respectively. The intasome particles were further purified by iterative rounds of tandem Ab-initio reconstruction and heterogenous refinement using 3 classes each time, resulting in 699,696 and 254,990 of CSC and STC particles, respectively. These were re-extracted with a pixel size of 1.9 Å and subjected to 3D classification in Relion-4.0 into 7 classes (CSC) or 5 classes (STC) without imposing symmetry and using the corresponding Ab-initio models, low-pass filtered to 60 Å as references. 359,894 CSC particles and 60,999 STC particles belonging to the two best-defined 3D classes were re-extracted without binning and used for non-uniform refinement and CTF refinement in cryoSPARC with C2 symmetry imposed, resulting in reconstructions at 2.8 Å resolution. Further, to isolate CSC particles free from spurious vDNA molecules captured in the tDNA binding groove, the signal corresponding to the conserved intasome core (CIC, representing the most structurally stable region of the intasome) was subtracted in Relion-4.0, and the subtracted particles were subjected to 3D classification without re-alignment in Relion-4.0 with a focus mask around the contaminating DNA component, resulting in separation of particles into 2 classes with free and occupied tDNA binding groove; the former class, comprising 167,347 particles (Supplementary Fig. 9e), was used for the final non-uniform refinement in cryoSPARC resulting in the final CSC reconstruction at 2.8 Å resolution (Supplementary Fig. 7, Supplementary Table 2). The reported resolution metrics are according to the gold-standard Fourier shell correlation (FSC) 0.143 criterion^120,121^. Local resolution of the 3D reconstructions was estimated in cryoSPARC (Supplementary Fig. 7). To create illustrations, the final maps were filtered using DeepEMhancer with the tight target model^122^. For real-space refinement of the atomistic models, the reconstructions were sharpened and filtered in cryoSPARC based on local resolution metrics.

The present cryo-EM maps of the MVV CSC and STC intasomes are considerably more complete and detailed than the previous reconstructions^8,12^. Therefore, we reassembled the CSC model from scratch by rigid body fitting of the individual MVV IN (NTD, CCD, CTD) and LEDGF/p75 (IBD) domains from high-quality crystal structures (PDB IDs 3HPH, 5LLJ, 5T3A, 2B4J)^8,87,89^ in UCSF Chimera^123^. The model was improved by iterative manual building and real-space refinement in Coot^124^ and global real-space refinement in Phenix version 1.21.2-5419^125^. The improved CSC model served the basis for building and real-space refinement of the STC. The final CSC and STC models were refined with 12 and 10 IBD chains, respectively. The quality of the models was assessed using MolProbity^126^ (Supplementary Table 2). Structural figures were prepared using UCSF Chimera^123^ and PyMOL (www.pymol.org). Intasome surface calculations were done with get_area command in PyMOL with solvent_radius set to 1.4 and dot_solvent on.

### Reporting summary

Further information on research design is available in the Nature Portfolio Reporting Summary linked to this article.

## Supplementary information

Supplementary Information

Reporting Summary

Transparent Peer Review file

## Source data

Source Data

## Data Availability

The final cryo-EM reconstructions were deposited with the EMDB under accession codes EMD-54482 and EMD-54481 and fitted coordinates with the PDB under accession codes 9S29 and 9S28; raw cryo-EM images are available upon request. The initial MVV CSC volume used for 3D classifications, and the structural models used in model building are available from the EMDB: EMD_14860 and the PDB: 3HPH, 5LLJ, 5T3A, 2B4J. The crystal structure of the core nucleosome particle is available from the PDB: 1KX5. Raw Nanopore sequencing data for intasome integration sites in chromatinized yeast DNA and Widom601 arrays have been deposited in the NCBI Sequence Read Archive under BioProject accessions PRJNA1367643 and PRJNA1505736, respectively. Source data are provided with this paper.
