# Supplementary material for: Core nucleosomes are refractory to lentiviral DNA integration
Source: Nat Commun. 2026 Aug 18;17:9907. doi: 10.1038/s41467-026-76698-8 (PMC13582839; doi:10.1038/s41467-026-76698-8)
Supplement: Supplementary file 1 [file 41467_2026_76698_MOESM1_ESM.pdf]

Supplementary information

Core nucleosomes are refractory to lentiviral DNA integration

Joshua Hope[1], Emma Punch[1], Nicola J. Cook[1], Matthew R. Singer[1], Dhira Joshi[2], Parmit K. Singh[3], Andrea Nans[4], Nathan P. Sweeney[5], Willem Vanderlinden[6], Alan N. Engelman[3], and Peter Cherepanov[1,7]

[1] Chromatin Structure & Mobile DNA Laboratory, The Francis Crick Institute, London, UK.
[2] Chemical Biology Science Technology Platform, The Francis Crick Institute, London, UK.
[3] Department of Cancer Immunology and Virology, Dana-Farber Cancer Institute, Boston, Massachusetts, USA.
[4] Structural Biology Science Technology Platform, The Francis Crick Institute, London, UK;
[5] Cell and Gene Therapy, Medicines Research Centre, GSK, Stevenage, UK.
[6] School of Physics and Astronomy, University of Edinburgh, Edinburgh, UK.
[7] Department of Infectious Disease, South Kensington Campus, Imperial College London, London, UK.

Supplementary Figures: 9
Supplementary Tables: 2

a

GAATTCGATATCTCGGGTGATGCCGGATCCC**CTGGAGAATCCCGGTGCCGAGGCCGCTCAATTGGTCGTAGACAGCTCTAGCACCGCTTA** 90
AACGCACGTACGCGCTGTCCCCCGCGTTTTAACCGCCAAGGGGATTACTCCCTAGTCTCCAGGCACGTGTCACATATATACATCCTGTTC 180
CAGTGCCGGTGTCGCTTGATATCTCGGGTGATGCCGGATCCC**CTGGAGAATCCCGGTGCCGAGGCCGCTCAATTGGTCGTAGACAGCTCT** 270
AGCACCGCTTAAACGCACGTACGCGCTGTCCCCCGCGTTTTAACCGCCAAGGGGATTACTCCCTAGTCTCCAGGCACGTGTCACATATAT 360
ACATCCTGTTCCAGTGCCGGTGTCGCTTGATATCTCGGGTGATGCCGGATCCC**CTGGAGAATCCCGGTGCCGAGGCCGCTCAATTGGTCG** 450
TAGACAGCTCTAGCACCGCTTAAACGCACGTACGCGCTGTCCCCCGCGTTTTAACCGCCAAGGGGATTACTCCCTAGTCTCCAGGCACGT 540
GTCACATATATACATCCTGTTCCAGTGCCGGTGTCGCTTGATATCTCGGGTGATGCCGGATCCC**CTGGAGAATCCCGGTGCCGAGGCCGC** 630
TCAATTGGTCGTAGACAGCTCTAGCACCGCTTAAACGCACGTACGCGCTGTCCCCCGCGTTTTAACCGCCAAGGGGATTACTCCCTAGTC 720
TCCAGGCACGTGTCACATATATACATCCTGTTCCAGTGCCGGTGTCGCTTGATATCTCGGGTGATGCCGGATCCC**CTGGAGAATCCCGGT** 810
GCCGAGGCCGCTCAATTGGTCGTAGACAGCTCTAGCACCGCTTAAACGCACGTACGCGCTGTCCCCCGCGTTTTAACCGCCAAGGGGATT 900
ACTCCCTAGTCTCCAGGCACGTGTCACATATATACATCCTGTTCCAGTGCCGGTGTCGCTTGATATCTCGGGTGATGCCGGATCCC**CTGG** 990
AGAATCCCGGTGCCGAGGCCGCTCAATTGGTCGTAGACAGCTCTAGCACCGCTTAAACGCACGTACGCGCTGTCCCCCGCGTTTTAACCG 1080
CCAAGGGGATTACTCCCTAGTCTCCAGGCACGTGTCACATATATACATCCTGTTCCAGTGCCGGTGTCGCTTGATATCTCGGGTGATGCC 1170
GGATCCC**CTGGAGAATCCCGGTGCCGAGGCCGCTCAATTGGTCGTAGACAGCTCTAGCACCGCTTAAACGCACGTACGCGCTGTCCCCCG** 1260
CGTTTTAACCGCCAAGGGGATTACTCCCTAGTCTCCAGGCACGTGTCACATATATACATCCTGTTCCAGTGCCGGTGTCGCTTGATATCT 1350
CGGGTGATGCCGGATCCC**CTGGAGAATCCCGGTGCCGAGGCCGCTCAATTGGTCGTAGACAGCTCTAGCACCGCTTAAACGCACGTACGC** 1440
GCTGTCCCCCGCGTTTTAACCGCCAAGGGGATTACTCCCTAGTCTCCAGGCACGTGTCACATATATACATCCTGTTCCAGTGCCGGTGTC 1530
GCTTGATATCTCGGGTGATGCCGGATCCC**CTGGAGAATCCCGGTGCCGAGGCCGCTCAATTGGTCGTAGACAGCTCTAGCACCGCTTAAA** 1620
CGCACGTACGCGCTGTCCCCCGCGTTTTAACCGCCAAGGGGATTACTCCCTAGTCTCCAGGCACGTGTCACATATATACATCCTGTTCCA 1710
GTGCCGGTGTCGCTTGATATCTCGGGTGATGCCGGATCCC**CTGGAGAATCCCGGTGCCGAGGCCGCTCAATTGGTCGTAGACAGCTCTAG** 1800
CACCGCTTAAACGCACGTACGCGCTGTCCCCCGCGTTTTAACCGCCAAGGGGATTACTCCCTAGTCTCCAGGCACGTGTCACATATATAC 1890
ATCCTGTTCCAGTGCCGGTGTCGCTTGATATCTCGGGTGATGCCGGATCCC**CTGGAGAATCCCGGTGCCGAGGCCGCTCAATTGGTCGTA** 1980
GACAGCTCTAGCACCGCTTAAACGCACGTACGCGCTGTCCCCCGCGTTTTAACCGCCAAGGGGATTACTCCCTAGTCTCCAGGCACGTGT 2070
CACATATATACATCCTGTTCCAGTGCCGGTGTCGCTTGATATCTCGGGTGATGCCGGATCCC**CTGGAGAATCCCGGTGCCGAGGCCGCTC** 2160
AATTGGTCGTAGACAGCTCTAGCACCGCTTAAACGCACGTACGCGCTGTCCCCCGCGTTTTAACCGCCAAGGGGATTACTCCCTAGTCTC 2250
CAGGCACGTGTCACATATATACATCCTGTTCCAGTGCCGGTGTCGCTTGATATCGAATTC 2310

b

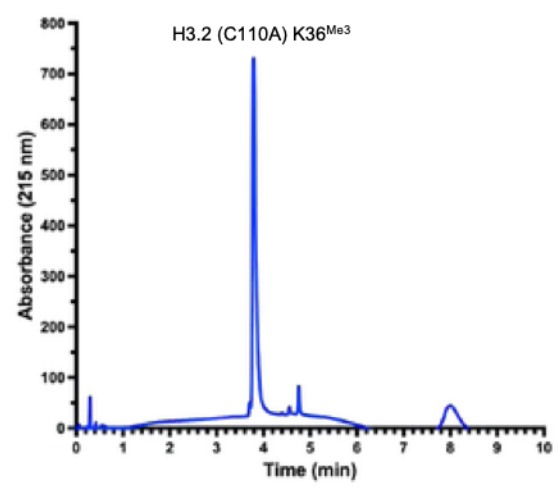

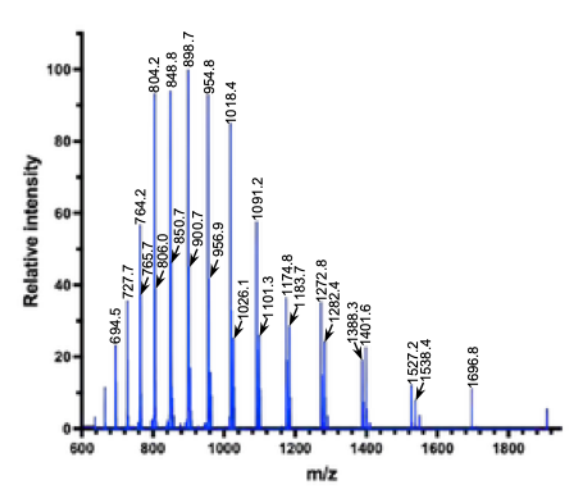

ARTKQTARKSTGGKAPRKQLATKAARKSAPATGGVKKPHRYRPGTVALREIRRYQKSTELLIRKLPFQRLVREIAQDFKTDLRFQSSA-VMALQEASEAYLVGLFEDTNLAAIHAKRVTIMPKDIQLARRIRGERA

Supplementary Figure 1 | Nucleotide sequence of the *Eco*RI fragment containing the array of twelve W601 nucleosome positioning sequences and validation of native semi-synthetic histone H3 trimethylated on Lys36. a, Individual W601 repeats are in bold print. *Eco*RI sites flanking the array are highlighted by grey shading, and *Eco*RV sites present in linker segments are underlined. Numbers to the right indicate base pair numbers of the right-most position in each line of the sequence. **b,** Final desulfurized histone H3 containing trimethylated Lys36 was analyzed by high-performance liquid reverse-phase chromatography coupled to intact protein mass spectrometry. Left, reverse-phase elution profile showing the position of the H3K36me3 protein. Right, full-scan mass spectrum of the main chromatographic peak; the mass/charge ratios (m/z) of the dominant ions are indicated. The calculated molecular weight (15,266.44) agrees well with the expected molecular weight (15,263.63). The sequence of the final protein is shown at the bottom of the panel, with the trimethylated lysine residue underlined.

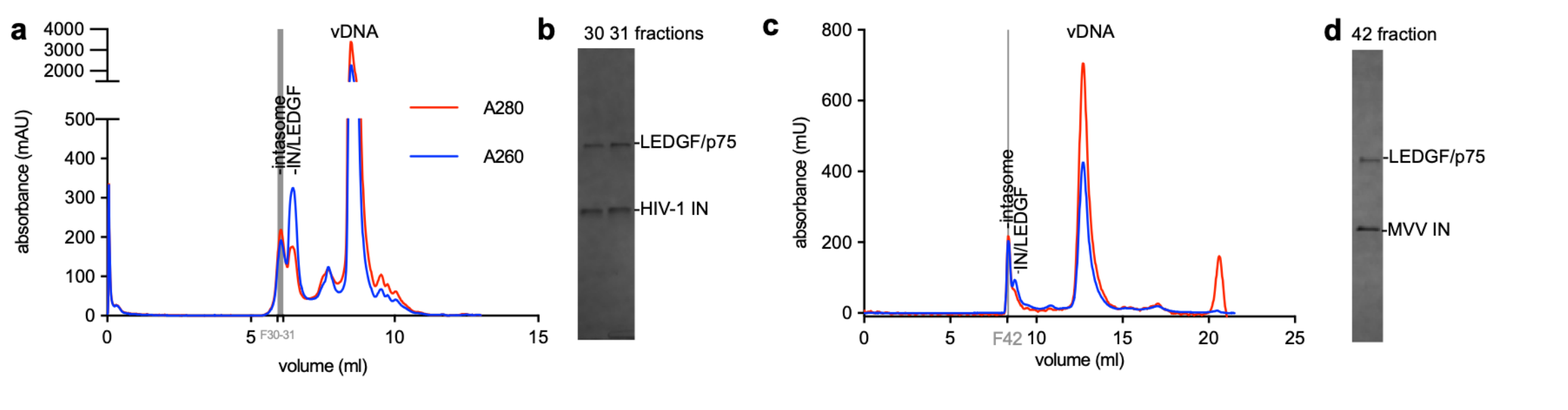

Supplementary Figure 2 | Lentiviral intasome purification. Chromatograms of HIV-1 (**a**) and MVV (**c**) intasomes purified by size exclusion chromatography. Indicated fractions (F30-31 or F42) on chromatograms (gray shading) were analyzed by SDS-PAGE for the HIV-1 (**b**) MVV (**d**) intasomes. The gels were stained with Coomassie Blue; migration positions of IN and co-purifying LEDGF/p75 are indicated. Source data are provided as a Source Data file.

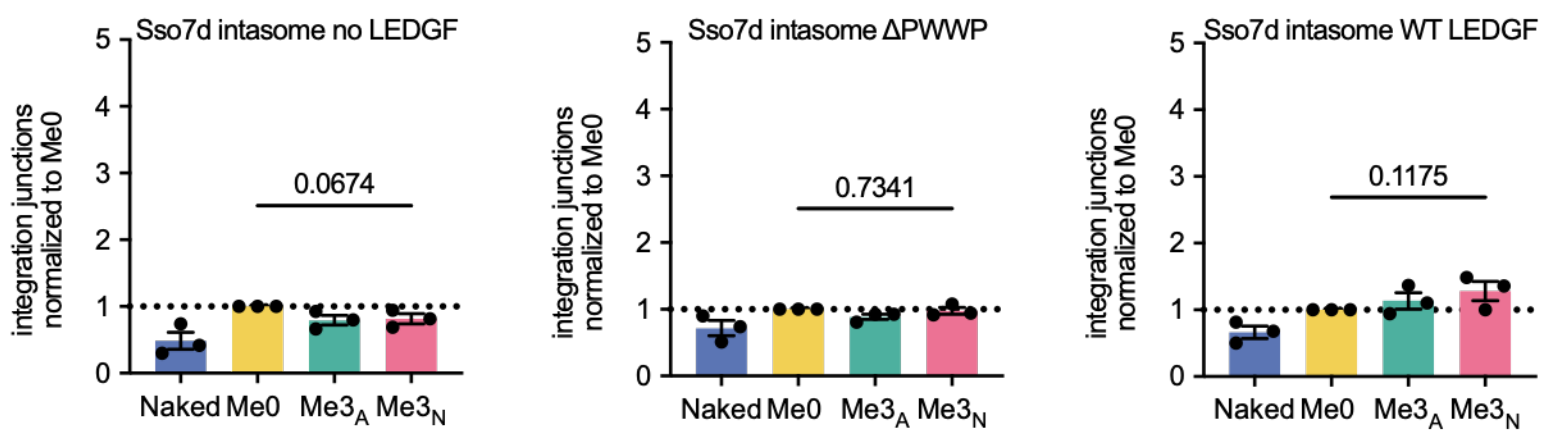

Supplementary Figure 3 | Integration of HIV-1 intasomes assembled using IN-Sso7d fusion into polynucleosomal arrays and H3K36Me3 methyl lysine analogue validation. Strand transfer activity of HIV-1 IN-Sso7d intasomes into naked or polynucleosomal arrays assembled with unmodified (Me0) or H3K36Me3 nucleosomes carrying the methyl-lysine analogue ($Me3_A$) or native chemical ligation ($Me3_N$) modification. Intasomes were assembled with no LEDGF/p75 (left), LEDGF/p75 lacking the PWWP domain (middle) or full-length LEDGF/p75 (right). Error bars are mean ± SEM; n = 3 independent stand transfer experiments. Plotted pairwise comparisons are two-sided Student's t-test with relevant p-values between the corresponding Me0 and $Me3_N$ conditions. Source data are provided as a Source Data file.

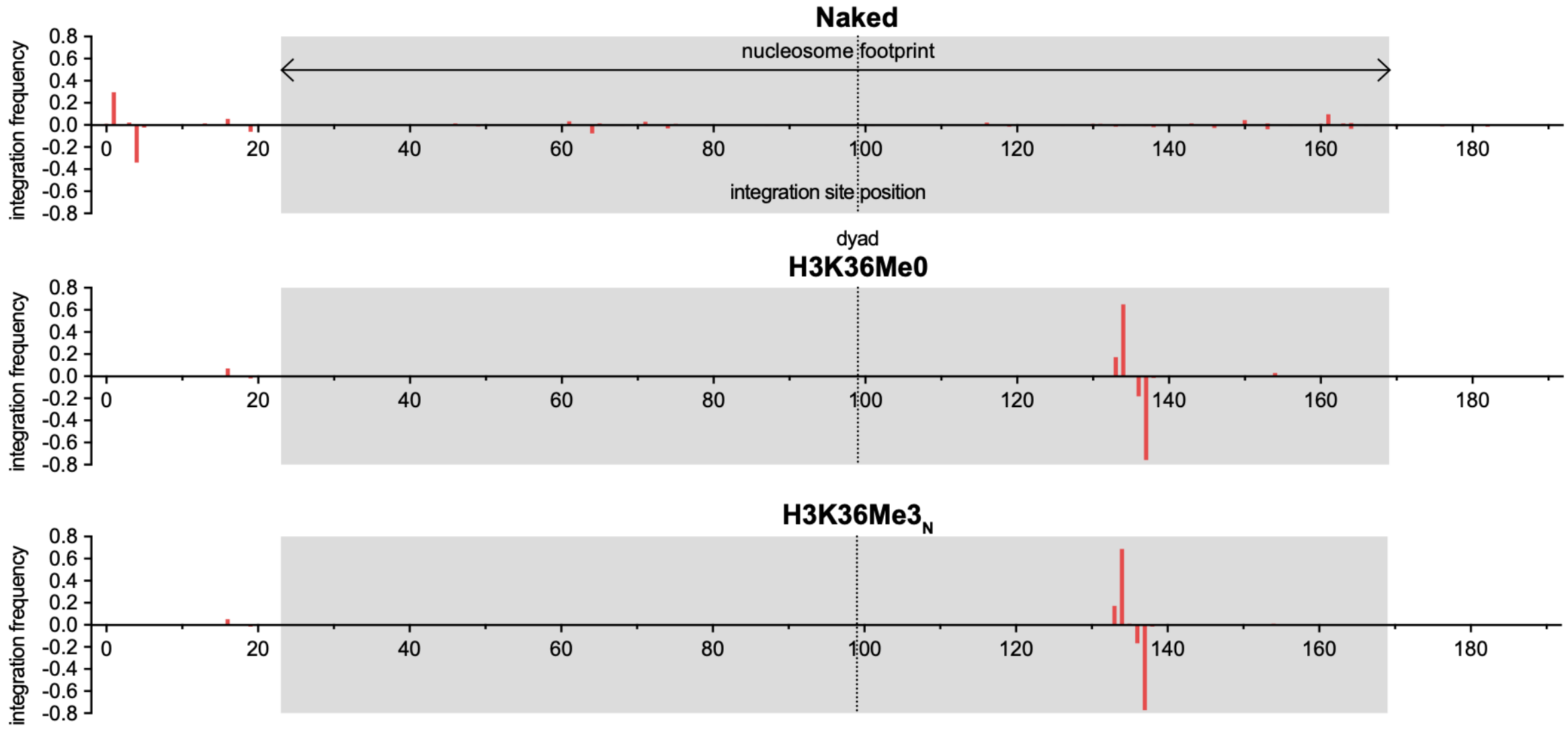

Supplementary Figure 4 | PFV integration sites on defined polynucleosomal arrays. Nanopore sequencing of PFV intasome-mediated integration into naked DNA, unmodified, and H3K36Me3N-modified polynucleosomal arrays. Data are presented as in Fig. 3a-b. Source data are provided as a Source Data file.

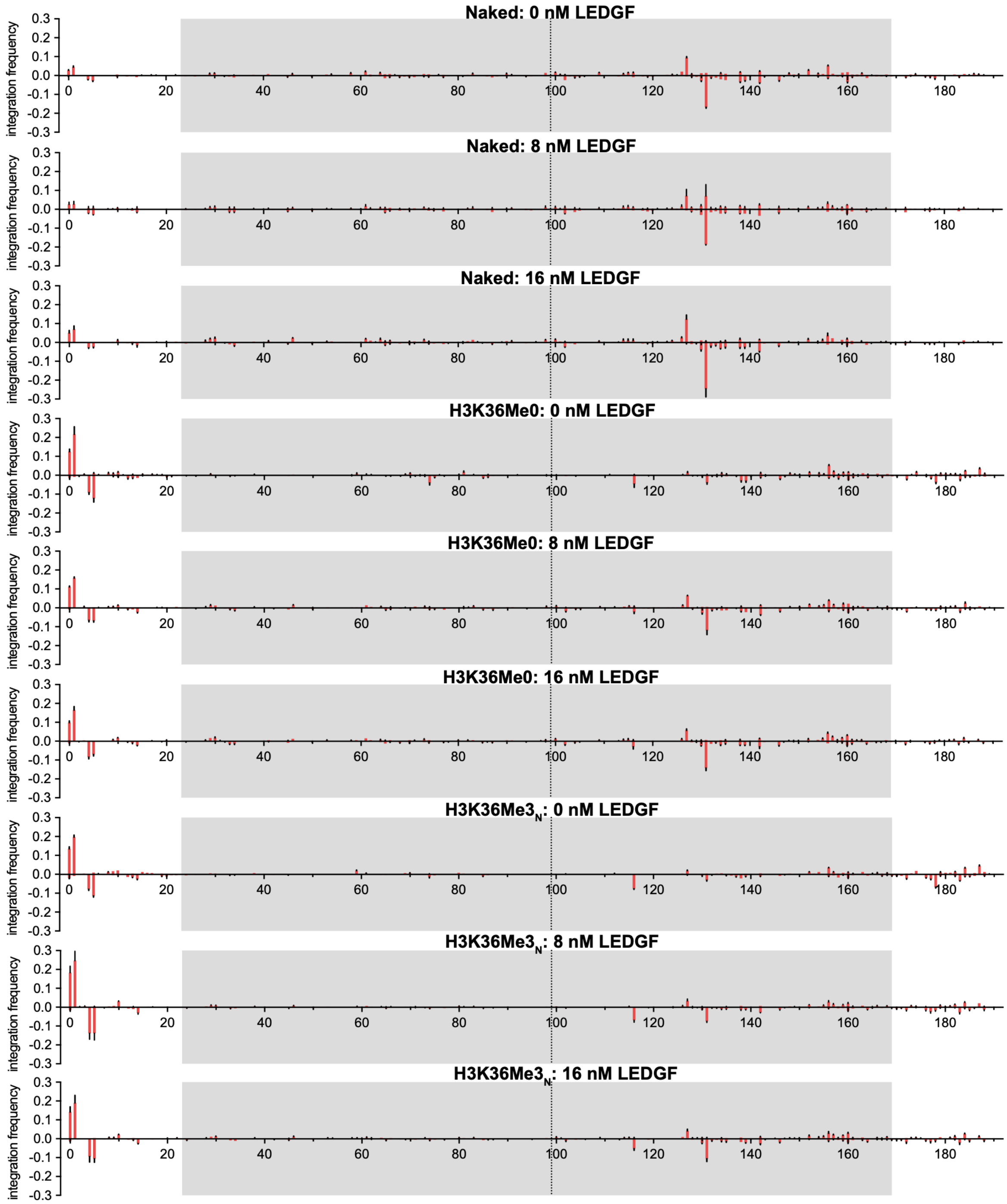

Supplementary Figure 5 | HIV-1 integration sites along defined polynucleosomal arrays. Integration was conducted in the presence of added 0, 8, or 16 nM LEDGF/p75. Data are presented as in Fig. 3a-b. Source data are provided as a Source Data file.

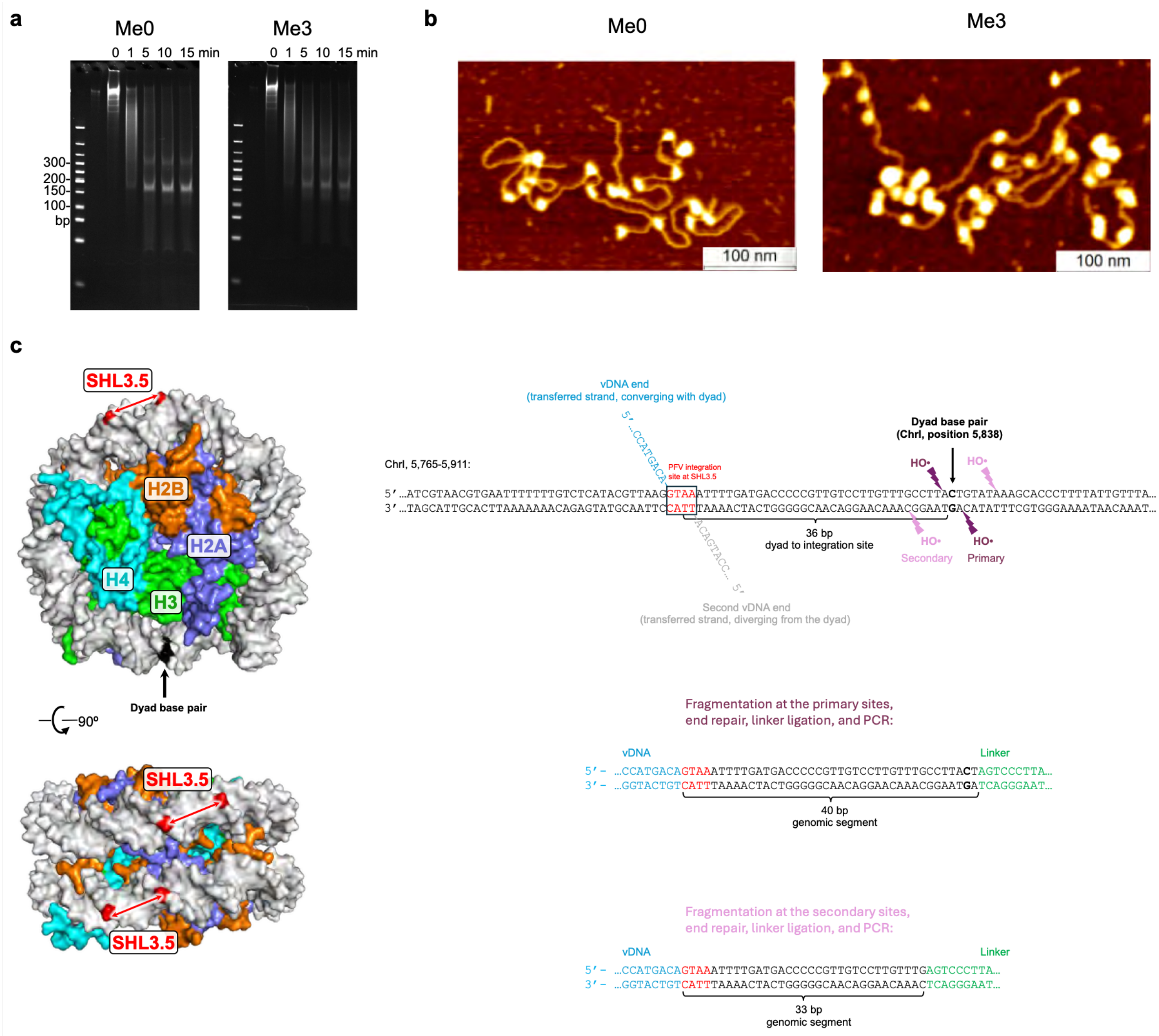

Supplementary Figure 6 | Validation of chromatinized yeast genomic DNA by micrococcal nuclease digestion and atomic force microscopy and chemical mapping of nucleosome dyad positions following strand transfer. a, K36Me0 and K36Me3$_N$ (indicated as Me0 and Me3, respectively) nucleosomes assembled on budding yeast genomic DNA were digested with micrococcal nuclease at 37˚C for indicated times. DNA products were deproteinized, separated by PAGE alongside 100-bp DNA ladder (left lane on each gel) and stained with ethidium bromide. Migration positions of DNA standards (100-300 bp) are indicated to the left. **b,** Nucleosomes assembled on yeast genomic DNA (see Methods) as visualized by atomic force microscopy in air. The low salt conditions of the deposition buffer favor open, "bead-on-a-string" configurations in which nucleosomes and connecting linker DNA can be observed. Scale bars are 100 nm. **c,** Left: Nucleosome structure (PDB ID 1KX5) in surface representation shown in two orthogonal views. Nucleosomal DNA is in light grey, with the scissile phosphodiesters of the primary PFV intasome target sites at super-helical location (SHL) ±3.5 in red and the dyad base pair in black (indicated with arrowhead). Histones H2A, H2B, H3, and H4 are shown in dark blue, orange, green, and sky-blue, respectively. Right: Derivation of LM-PCR product mapped following reaction with the PFV intasome and chemical cleavage of nucleosomal DNA. DNA sequence of yeast chromosome I positions 5,765-5,911, with observed integration site shown in red and boxed, located in the vicinity of a previously characterized yeast nucleosomal dyad at position 5,838 (indicated with black arrowhead)[73]. Insertion of PFV viral (vDNA) 3' end (blue lettering) allows annealing of the

forward PCR primer, specific to vDNA. Fenton reaction-dependent yeast DNA fragmentation at primary and secondary sites (indicated with dark and light purple flash symbols, respectively) following end repair and linker ligation allows linker primer annealing. The structures of the PCR products generated following cleavage at the primary and secondary cleavage sites are shown in the middle and bottom panels, respectively. Source data are provided as a Source Data file.

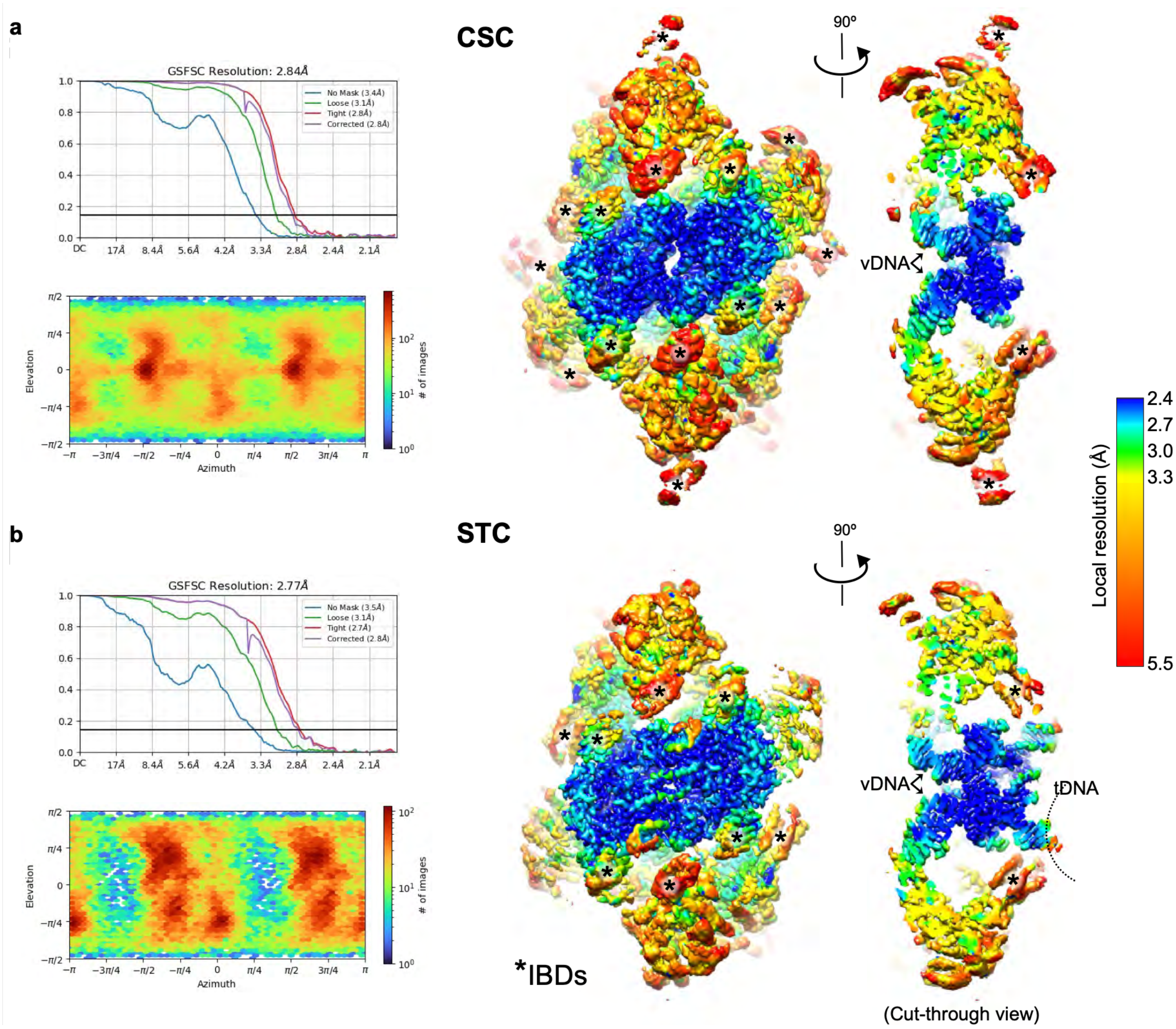

Supplementary Figure 7 | Resolution metrics of the final MVV cleaved synaptic complex (CSC, a) and strand transfer complex (STC, b) reconstructions. a-b, Top left: half-map Fourier shell correlation (FSC) without mask (blue line), with loose mask (green line), with tight mask (red line), and optimized mask (purple line). The FCS threshold of 0.143 (horizontal blue line) corresponds to a global resolution of 2.84 and 2.77 Å for CSC (a) and STC (b), respectively. Bottom left: viewing angle distribution of assigned to individual particle images that contributed to the final 3D reconstruction. Right: cryo-EM maps, colored by local resolution as per color key. Rotated maps shown in cut-through view. Map features corresponding to bound IBDs are indicated with asterisks.

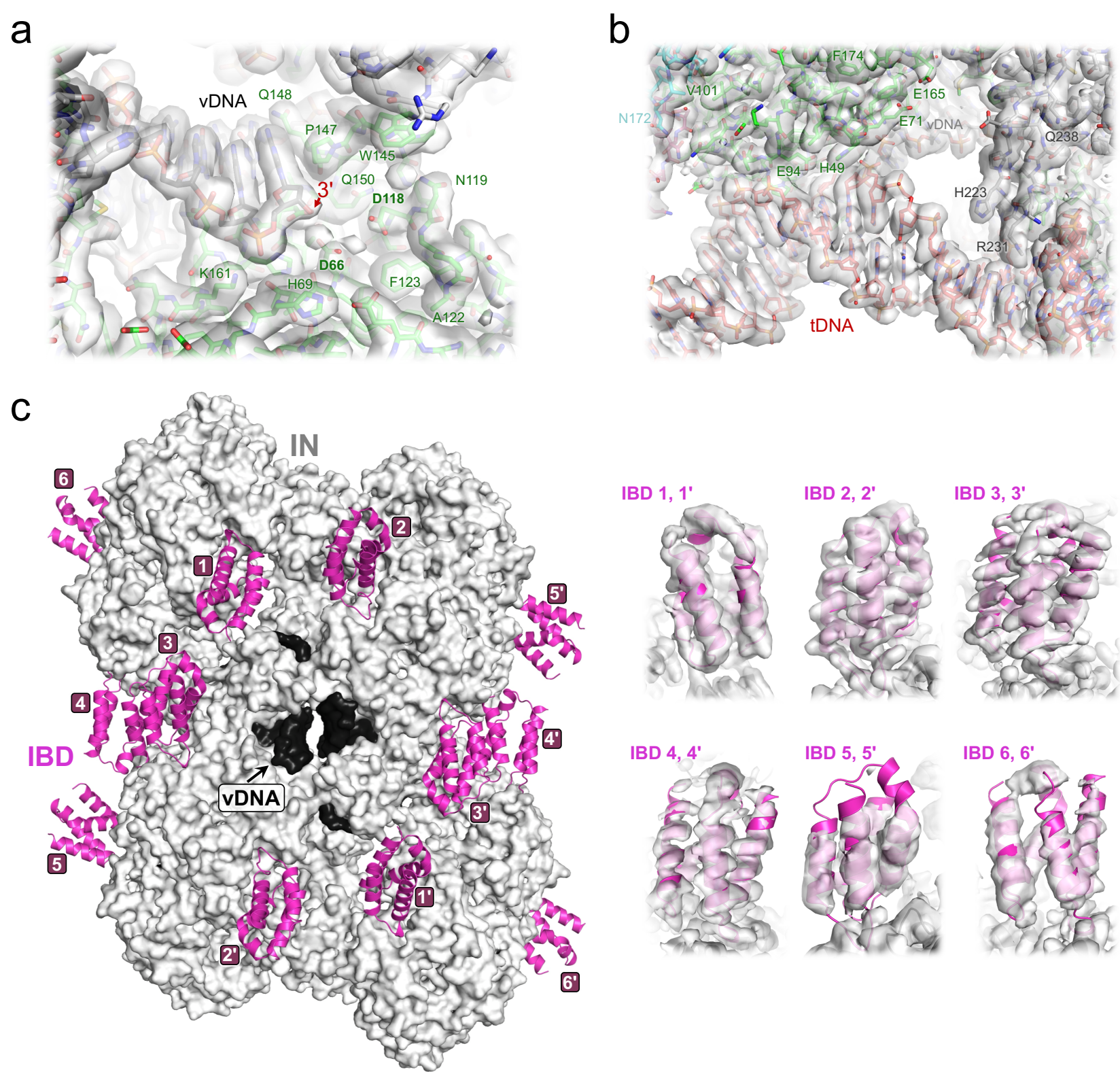

Supplementary Figure 8 | Cryo-EM map regions corresponding to the conserved intasomal core (*a*, *b*) and modeled LEDGF integrase binding domains (IBDs, *c*). *a*, *b*, Cryo-EM map of the active site of the MVV cleaved synaptic complex (CSC) intasome (a) and target DNA (tDNA) within the strand transfer complex (STC) intasome (b). Cryo-EM maps are shown as semi-transparent surfaces. Protein, viral DNA (vDNA), and tDNA are shown in stick representation, with carbon atoms colored green for IN chain A, grey for IN chains N and F, black for vDNA, and orange for tDNA. Other atoms are colored according to the standard scheme: nitrogen, blue; oxygen, red; and phosphorus, bright orange. vDNA, tDNA, and selected amino acid residues are labeled. ***c*,** *Left*, model of the MVV CSC intasome, with IN and vDNA shown as grey and black surfaces, respectively. Modeled LEDGF/p75 IBDs (IBD 1 - IBD 6 and their symmetry-related equivalents, IBD 1′ - IBD 6′) are shown as magenta cartoons. *Right*, snapshots of the cryo-EM map regions corresponding to each modeled IBD.

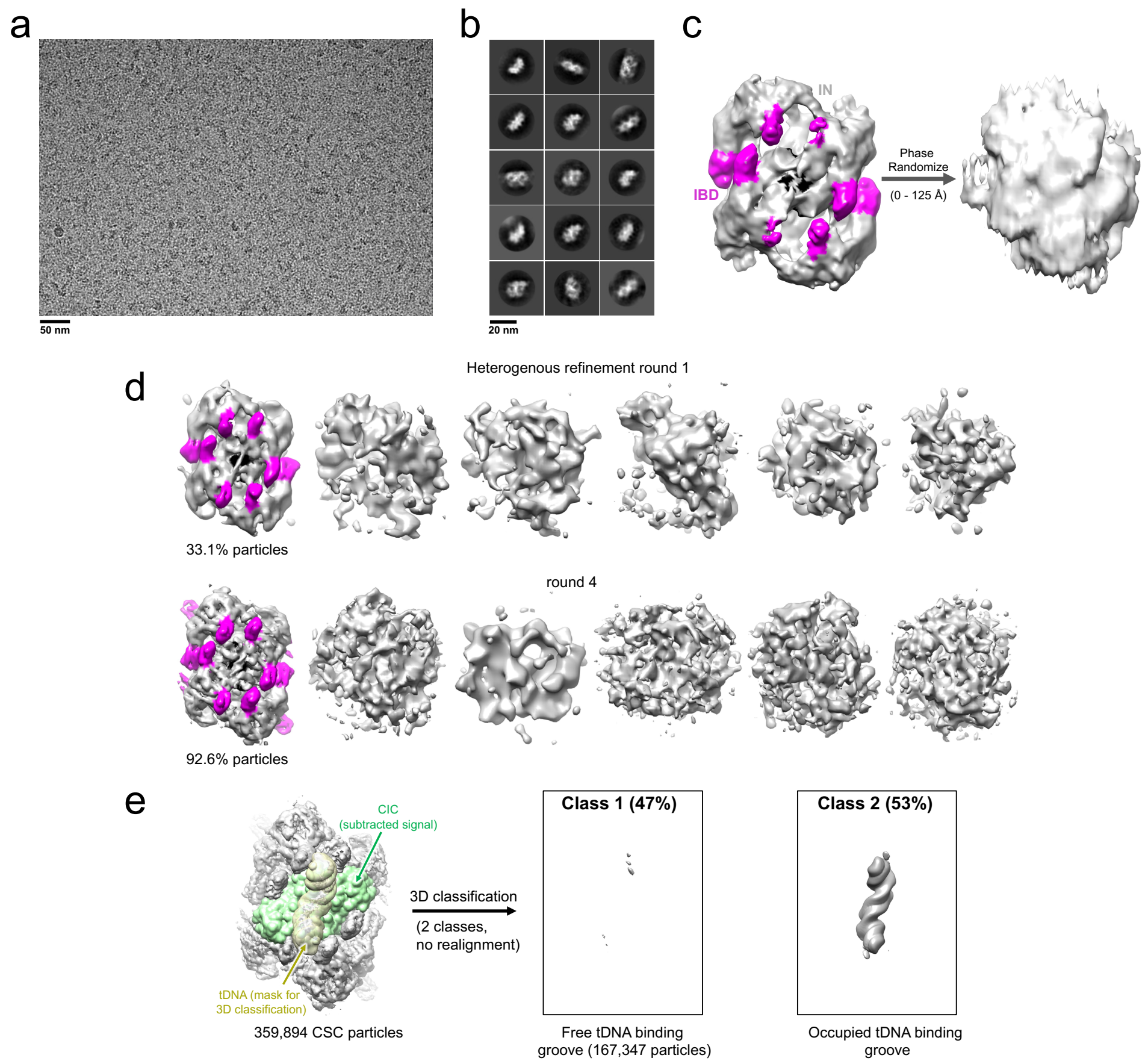

Supplementary Figure 9 | Cryo-EM image processing and 3D reconstruction of the MVV intasome. a, Representative micrograph of MVV intasomes captured on derivatized graphene oxide. Scale bar is 50 nm. **b,** 2D class averages of the cleaved synaptic complex (CSC) imaged on Talos transmission electron microscope, using Falcon III direct electron detector camera in linear mode. Scale bar corresponds to 20 nm. **c,** Particles selected by 2D classification followed by 3D classification were used for non-uniform refinement in cryoSPARC reconstruction (left image see Methods for specific details). The image shows unsharpened cryo-EM map with features corresponding to IN and bound IBDs in grey and purple, respectively. To create a junk trap, the volume was subjected to phase randomization of Fourier components with spatial frequences exceeding 1/125 $Å^{-1}$ (right image). **d,** MVV CSC intasome particle set purification through rounds 1 and 4 of heterogenous refinements using phase-randomized junk trap (prepared as shown in a). Each round started with a single copy of input CSC volume (a, middle image) and five copies of the phase-randomized trap, all low-pass filtered to 20 Å (see Methods for specific details). **e,** Strategy used to isolate CSC particles with an unoccupied target DNA (tDNA)-binding groove. *Left*: Consensus reconstruction of 359,894 CSC particles (grey surface), with soft masks encompassing the conserved intasomal core (CIC; the best-resolved region of the reconstruction) and tDNA shown as semitransparent green and yellow surfaces, respectively. Following subtraction of the CIC signal, particles were subjected to 3D classification into two classes without realignment. *Right*: Masked class averages of the resulting signal-subtracted particles. Class 1, comprising 47% of the input particles and lacking detectable density within the tDNA mask, was selected for the final reconstruction after reverting to the corresponding unsubtracted particles.

Supplementary Table 1. Co-mapping integration sites and nucleosomal dyads on chromatinized yeast genomic DNA.

**Intasome**	**Target**	**GEO accession codes** [1]	**N unique fragments mapped** [2]	**N unique fragments <150 bp** [3]
**PFV**	Naked DNA	GSM9313407 - GSM9313409	482,619	137,933
	H3K36Me0	GSM9313403 - GSM9313404	57,475	41,249
	H3K36Me3$_N$	GSM9313405 - GSM9313406	37,533	26,658
**HIV-1**	Naked DNA	GSM9313418 - GSM9313423	228,911	94,508
	H3K36Me0	GSM9313410 - GSM9313412	168,461	61,544
	H3K36Me3$_N$	GSM9313413 - GSM9313414	604,693	165,505
	H3K36Me3$_N$ + 8 nM LEDGF	GSM9313415 - GSM9313417	66,505	26,294
**MVV**	Naked DNA	GSM9313431 - GSM9313433	248,091	117,017
	H3K36Me0	GSM9313424 - GSM9313426	161,369	64,012
	H3K36Me3$_N$	GSM9313427 - GSM9313428	89,679	55,072
	H3K36Me3$_N$ + 8 nM LEDGF	GSM9313429 - GSM9313430	38,298	20,512

[1] Sample accession codes (https://www.ncbi.nlm.nih.gov/geo/query/acc.cgi?acc=GSE310967).
[2] Number of unique PCR products mapped for each sample.
[3] Number of mapped PCR fragments with lengths less than 150 bp, used to assemble histograms in Fig. 4b.

Supplementary Table 2. Cryo-EM data collection, image processing and model refinement.

**Data collection**	**CSC**	**STC**
Microscope, operating voltage	Titan Krios G2, 300 keV	Titan Krios G2, 300 keV
Detector	Falcon 4i	Falcon 4i
Magnification (nominal)	130,000	130,000
Pixel size (Å)	0.95	0.95
Underfocus range (nominal, µm)	1.5 - 3.3	1.5 - 3.3
Number of EER frames per movie	1,674	1,674
Total electron fluence (e/Å^2)	41.0	41.0
Automation software	EPU	EPU
Number of micrograph movies used	33,853	27,316
**Reconstruction**		
**EMDB ID**	**EMD-54482**	**EMD-54481**
Software for 2D classification	cryoSPARC	cryoSPARC
Software for 3D classification	cryoSPARC, Relion	cryoSPARC, Relion
Software for final reconstruction	cryoSPARC	cryoSPARC
Number of refined particles	167,347	60,999
Symmetry	*C2*	*C2*
Global resolution (FSC 0.143, Å)	2.84	2.77
Map resolution range (Å)	2.4 - 6.0	2.4 - 6.0
Map sharpening B factor	-110.8	-70.8
**Model refinement**		
**PDB ID**	**9S29**	**9S28**
Software for real-space refinement	Phenix	Phenix
Model composition:		
Number of non-hydrogen atoms	43,436	41,690
Number of protein residues	5,162	4,790
Number of nucleotides	80	140
Number of ligands (Zn)	16	14
B factors (Å^2):		
Protein	87.9	108
DNA	61.2	47.7
Ligands	112	151
Real-space correlation coefficients:		
Overall (CC_{mask}, CC_{box}, CC_{peaks}, CC_{volume})	0.70, 0.64, 0.63, 0.68	0.72, 0.68, 0.64, 0.70
Conserved intasomal core (CC_{mask})	0.85	0.84
R.m.s. deviations:		
Bond lengths (Å)	0.002	0.007
Bond angles (°)	0.418	0.431
Model validation:		
MolProbity score	1.31	1.30
Clash score	4.33	4.05
Poor rotamers (%)	1.06	1.14
CaBLAM outliers (%)	1.70	1.74
Ramachandran plot quality (%)		
Favored, Disallowed	97.61, 0	97.68, 0
