# Supplementary material for: Core nucleosomes are refractory to lentiviral DNA integration
Source: Nat Commun. 2026 Aug 18;17:9907. doi: 10.1038/s41467-026-76698-8 (PMC13582839; doi:10.1038/s41467-026-76698-8)
Supplement: Supplementary file 2 [file 41467_2026_76698_MOESM2_ESM.pdf]

nature portfolio

Corresponding author(s): Cherepanov, Hope

Last updated by author(s): Oct 19, 2025

Reporting Summary

Nature Portfolio wishes to improve the reproducibility of the work that we publish. This form provides structure for consistency and transparency in reporting. For further information on Nature Portfolio policies, see our Editorial Policies and the Editorial Policy Checklist.

Statistics

For all statistical analyses, confirm that the following items are present in the figure legend, table legend, main text, or Methods section.

n/a	Confirmed	
☐	☒	The exact sample size (*n*) for each experimental group/condition, given as a discrete number and unit of measurement
☐	☒	A statement on whether measurements were taken from distinct samples or whether the same sample was measured repeatedly
☐	☒	The statistical test(s) used AND whether they are one- or two-sided<br>*Only common tests should be described solely by name; describe more complex techniques in the Methods section.*
☒	☐	A description of all covariates tested
☒	☐	A description of any assumptions or corrections, such as tests of normality and adjustment for multiple comparisons
☒	☐	A full description of the statistical parameters including central tendency (e.g. means) or other basic estimates (e.g. regression coefficient) AND variation (e.g. standard deviation) or associated estimates of uncertainty (e.g. confidence intervals)
☐	☒	For null hypothesis testing, the test statistic (e.g. F, t, r) with confidence intervals, effect sizes, degrees of freedom and P value noted<br>*Give P values as exact values whenever suitable.*
☒	☐	For Bayesian analysis, information on the choice of priors and Markov chain Monte Carlo settings
☒	☐	For hierarchical and complex designs, identification of the appropriate level for tests and full reporting of outcomes
☒	☐	Estimates of effect sizes (e.g. Cohen's *d*, Pearson's *r*), indicating how they were calculated

Our web collection on statistics for biologists contains articles on many of the points above.

Software and code

Policy information about availability of computer code

Data collection	Real-time quantitative PCR data were acquired using either a QuanStudio 7 Flex, or ViiA7 system and software (Applied Biosystems).<br>Electrophoresis gel images were acquired on a Gel Doc EZ Gel Documentation System (Bio-Rad).<br>Oxford Nanopore Technologies Long Read Sequencing of integration sites on W601 poly-nucleosomal arrays was acquired on an r10.4 or r10.4.1. Base-calling was done with either Guppy or Dorado (Oxford Nanopore Technologies) using the super accurate models.<br>Oxford Nanopore Technologies Long Read Sequencing of integration sites into chromatinized natural DNA fragments was was acquired and base-called by Full Circle Lab, Imperial College London, UK.<br>Nanowizard 4 (JPK, Berlin, Germany) was used to record AFM images.<br>Cryo-EM data collection and software is described in the methods section (EPU v3, available from Thermo Fisher).
Data analysis	Data analysis and validation was conducted using commercially or publicly available software, details of which are in the manuscript.<br>real-time quantitative PCR data and Oxford Nanopore sequencing data was analyzed in Prism GraphPad v11 with analysis indicated in the manuscript.<br>Integration sites into W601 poly-nucleosomal arrays were analyzed using minimap2, SAMtools and SeqKit.<br>Integration sites into chromatinized natural DNA fragments were mapped and analyzed using BLAT v3.5 and Bedtools v2.30.0.<br>Student's statistical tests were performed and data were plotted in Prism GraphPad v11.<br>R version 4.6.1 was used to compute Chi2 statistics and Cramér's V with confidence intervals.<br>AFM data were analysed using SPIP software (v6.1.1, Image Metrology).<br>Cryo-EM image processing and 3D volume reconstruction: Relion, v4.0 and cryoSPARC v4.1.0.<br>Cryo-EM map post-processing: DeepEMhancer (https://github.com/rsanchezgarc/deepEMhancer).

Cryo-EM structure real-space refinement: Coot v0.9 and Phenix v1.21.2-5419.
Final model validation: MolProbity (https://molprobity.biochem.duke.edu/).

For manuscripts utilizing custom algorithms or software that are central to the research but not yet described in published literature, software must be made available to editors and reviewers. We strongly encourage code deposition in a community repository (e.g. GitHub). See the Nature Portfolio guidelines for submitting code & software for further information.

Data

Policy information about availability of data

All manuscripts must include a data availability statement. This statement should provide the following information, where applicable:

- Accession codes, unique identifiers, or web links for publicly available datasets
- A description of any restrictions on data availability
- For clinical datasets or third party data, please ensure that the statement adheres to our policy

The final cryo-EM reconstructions were deposited with the EMDB under accession codes EMD-54482 (CSC) and EMD-54481 (STC) and fitted coordinates with the PDB under accession codes 9S29 (CSC) and 9S28 (STC). Nanopore sequencing of PFV, HIV-1, and MVV intasome integration sites into W601 arrays and chromatinized yeast DNA data are available from Zenodo (https://doi.org/10.5281/zenodo.17566487) and NCBI GEO (https://www.ncbi.nlm.nih.gov/geo/query/acc.cgi?acc=GSE310967), respectively.

Research involving human participants, their data, or biological material

Policy information about studies with human participants or human data. See also policy information about sex, gender (identity/presentation), and sexual orientation and race, ethnicity and racism.

Reporting on sex and gender	n/a
Reporting on race, ethnicity, or other socially relevant groupings	n/a
Population characteristics	n/a
Recruitment	n/a
Ethics oversight	n/a

Note that full information on the approval of the study protocol must also be provided in the manuscript.

Field-specific reporting

Please select the one below that is the best fit for your research. If you are not sure, read the appropriate sections before making your selection.

[x] Life sciences [ ] Behavioural & social sciences [ ] Ecological, evolutionary & environmental sciences

For a reference copy of the document with all sections, see nature.com/documents/nr-reporting-summary-flat.pdf

Life sciences study design

All studies must disclose on these points even when the disclosure is negative.

Sample size	No statistical methods were used to predetermine sample sizes.Sample sizes for biochemical assays were based on established practice and experimental reproducibility, with independent experiments generally performed in triplicate. For integration-site sequencing, dataset sizes were determined by sequencing yield, and all integration events satisfying predefined mapping criteria were included. For AFM, sufficient independent particles were analysed to characterize the distribution of nucleosome occupancies and linker lengths. Cryo-EM dataset sizes were not predetermined; data collection and particle numbers were determined empirically by sample quality and the amount of data required to obtain converged reconstructions at the reported resolutions. Exact sample sizes and numbers of independent experiments are provided in the Methods, figure legends and Supplementary Information.
Data exclusions	During cryo-EM image analysis, particle images belonging to 2D or 3D classes representing noise or lacking identifiable features were excluded, as explained in the Methods section. No data were excluded from biochemical experiments.
Replication	Where statistics are presented, experiments were repeated independently at least three times as indicated in the figure legends. For real-time quantitative PCR experiments, each sample was also tested in technical triplicate, the mean of which was calculated and used for downstream analysis. The data described in the manuscript are reproducible.
Randomization	Randomization was employed during cryo-EM 3D refinement in Relion and CryoSPARC, which use independent random subsets of particle images to avoid over-refinement. Given the nature of the biochemical experiments, samples were not randomized.
Blinding	Investigators were not blinded to allocation during experiments and outcome assessment. Blinding is not standard practice in this type of biochemical experiments, and was not done due to practical constraints.

Reporting for specific materials, systems and methods

We require information from authors about some types of materials, experimental systems and methods used in many studies. Here, indicate whether each material, system or method listed is relevant to your study. If you are not sure if a list item applies to your research, read the appropriate section before selecting a response.

Materials & experimental systems

n/a	Involved in the study	
x		Antibodies
	x	Eukaryotic cell lines
x		Palaeontology and archaeology
x		Animals and other organisms
x		Clinical data
x		Dual use research of concern
x		Plants

Methods

n/a	Involved in the study	
x		ChIP-seq
x		Flow cytometry
x		MRI-based neuroimaging

Eukaryotic cell lines

Policy information about cell lines and Sex and Gender in Research

Cell line source(s)	The human cell lines described as LHKO and the parental HEK293T have been reported and validated (see reference 12 in the manuscript).
Authentication	LHKO cells were validated in the original publication by PCR on genomic DNA of the knockout and immunoblotting for LEDGF/p75 and HRP2 protein. Cells were authenticated in our lab by immunoblotting in a previous publication (see reference 12 in the manuscript)
Mycoplasma contamination	The cells were confirmed to be free of mycoplasma contamination by the Francis Crick Institute Tissue Culture Scientific Technology Platform using commercial kits.
Commonly misidentified lines (See ICLAC register)	n/a

Plants

Seed stocks	n/a
Novel plant genotypes	n/a
Authentication	n/a
