# Supplementary material for: Core nucleosomes are refractory to lentiviral DNA integration
Source: Nat Commun. 2026 Aug 18;17:9907. doi: 10.1038/s41467-026-76698-8 (PMC13582839; doi:10.1038/s41467-026-76698-8)
Supplement: Supplementary file 3 [file 41467_2026_76698_MOESM3_ESM.pdf]

nature portfolio

Peer Review File

Core nucleosomes are refractory to lentiviral DNA integration

Corresponding Author: Dr Peter Cherepanov

This file contains all reviewer reports in order by version, followed by all author rebuttals in order by version.

Version 0:

Reviewer comments:

Reviewer #1

(Remarks to the Author)
The manuscript by Joshua Hope et al. presents exciting findings regarding target site selection of lentiviral intasomes in the context of chromatinized target DNA. The major claim of the study is that HIV and MVV integrate their genome into linker DNA rather than nucleosomal (wrapped) DNA. This finding contrasts results from earlier studies using less well-defined integrase-vDNA assemblies, and high resolution structural data of the well-defined prototype foamy virus intasome docked on a nucleosome as target capture complex and strand transfer complex. As the intasome core tetramer is quasi invariant along PFV and other characterized intasomes (MMTV, MVV, HIV), it was expected that nucleosomal DNA would be the preferred target independent of the retrovirus genus. Indeed, target DNA bending as required for aligning the scissile phosphodiester bonds in the intasome active site, could be (largely) offset by curvature induced via nucleosome wrapping. The observation that lentiviral MVV and HIV intasomes prefer linker DNA over nucleosomal DNA is thus surprising and an important advance. The question remains why lentiviral intasomes behave differently from the archetypical PFV intasome. In this manuscript, Hope and colleagues explain the shift away from the nucleosome core and into the linker DNA, by considering the structure (cryoEM) of MVV intasomes incubated with the host cofactor protein LEDGF/p75. In this structure, the interface of the intasome that faces the target DNA is decorated with several copies of the integrase-binding domain of LEDGF/p75 that would sterically clash with the nucleosome body.

The manuscript is well-written, with an extensive methods section and appropriate statistical analysis. I have the following questions and comments.

1. The role of chromatin structure is not clearly addressed.
a. The authors have used 12-mers of the W601 sequence interspersed with 44bp linker DNA, as well as sheared genomic DNA from budding yeast, for chromatin array reconstitution. W601 nucleosome positioning sequences wrap into nucleosomes with binding free energy lower by ~5 kBT with respect to strong natural 5S positioning sequence (Lowary and Widom, JMB 1998). Yeast chromatin is characterized by very short linker lengths (15-20 bp, Horz and Zachau JMB 1980) and has a more open configuration as compared to chromatin in mammals. In both cases, these thermodynamical and structural features are distinct from the chromatin naturally encountered by lentiviral PICs. Can the authors comment on how the current finding can be extrapolated to human chromatin?
b. The chromatin reconstituted on sheared yeast genomic DNA is not assessed for quality. The assay that uses the Fenton reaction combined with integration site sequencing is very elegant and establishes a ruler with respect to the nucleosome dyad. Nevertheless, nucleosome density arguably plays a role with regards to e.g. linker DNA bending. Here, a single molecule (i.e. Nanopore) sequencing or imaging (for example EM) approach would be useful.
c. LEDGF/p75 has been shown to affect the nucleosomal barrier to transcription (LeRoy et al, Sci Adv 2019), suggesting that it can have (an additional) role in remodeling nucleosomes and chromatin making the target either more or less accessible.
2. The cryo-EM data is recorded on samples of Maede Visna Virus intasomes in the presence of 0.25 mg/mL or approximately 4 micromolar of LEDGF/p75 (MW 62 kDa). This is orders of magnitude higher as compared to that compared to integration assays (8-16 nM). What is the logic to saturate LEDGF binding pockets beyond what might be relevant for the biological effect? How does this compare to the concentration of LEDGF/p75 in the cell?
3. The authors claim that the PICs remain bound with LEDGF/p75, and refer to Llano et al., J. Virol. 2004 to substantiate this claim. However, whereas Llano et al. used cytoplasmal PIC fractions, here the authors use crude nuclear PIC extract.
4. On line 366, the authors write: “we propose that multiple LEDGF/p75 molecules associated with the intasome function as an entropic filter”. It is not clear what the authors mean with “entropic filter”. Does the multivalency of PWWP readers associated with the intasome reduce its possible degrees of freedom, or does the multivalency warrant integration in K36Me3 rich genomic areas, “filtering out” epigenetic noise?

5. On line 360, it is stated that “the disordered region between PWWP and IBD is a predicted average of ~80 nm”. Assuming the disordered region comprises 255 amino acids and taking the length per amino acid 0.38 nm, the contour length of the disordered region is ~97 nm. However, the disordered region is clearly not perfectly and fully extended. Predicting the extension of the region as a worm-like chain with bending persistence length 0.4 nm gives and end-end distance of 7.2 nm (see Kjaergaard et al., Methods in Enzymology, 2021). Similarly models that consider self-avoidance (self-avoiding walk) and include sequence features (ALBATROSS; Lotthammer et al., Nature Methods 2024) give mean values for the end-end distance of ~10nm and 12.4 nm. Extensions beyond 30 nm are extremely uncommon.
Together, the above comments indicate that the manuscript would be suitable for publication in Nature Communications with relatively minor changes to the submitted version.

Reviewer #3

(Remarks to the Author)
Hope et al. show that lentiviral integrases have a strong bias to integrate into the linker DNA regions within H3K36Me3-enriched chromatin because, while lentiviral intasomes disfavor core nucleosomes, LEDGF/p75 recruits them to H3K36Me3N-containing polynucleosomal arrays. This is high-quality work that provides novel insights into the mechanism of replication of important viral pathogens. I would suggest clarification on the following aspect, though.

The authors state that

LEDGF/p75 alters the target DNA binding platform of the lentiviral intasome, imposing significant constraints on the path and configuration of target DNA to impede nucleosome engagement.

The cryo-EM structures reveal that bound IBDs remodel the tDNA binding platform of the intasome, acting as physical barriers to facilitate optimal linker DNA engagement for integration, while excluding the approach of more bulky core nucleosome particles.

LEDGF/p75 IBDs reshape the tDNA binding platform of the lentiviral intasomes, while multiple LEDGF/p75 molecules associated with the intasome function as an entropic filter, excluding H3K36Me0 nucleosomes.

These claims appear to be very hand-wavy.

Is the target DNA or the target DNA-binding platform actually shaped differently in the new structure compared to the previous STC structures determined with a less-saturating amount of LEDGF/p75 (besides having more LEDGF/p75 molecules bound)? It is not clear how LEDGF/p75 selectively interferes with the binding of DNA in nucleosomes. To support the entropic filter hypothesis, the authors might want to test if IBD by itself reduces integration into unmodified chromatin, similarly to the full-length LEDGF/p75 (Fig. 1e).

In addition, the authors should estimate the PIC or intasome concentration used in the in vitro assays. In Fig. 1, 2 nM of LEDGF/p75 is saturating, whereas in Fig. 2, 32 nM is still not saturating. Is the discrepancy due to a difference in the intasome concentration?

Reviewer #4

(Remarks to the Author)
In this manuscript, Hope and colleagues investigate how the transcriptional co-activator LEDGF/p75 guides lentiviral integration within chromatin. Using native HIV-preintegration complexes and in vitro assembled lentiviral intasomes with defined chromatinized templates, the authors show that LEDGF/p75 promotes integration in linker DNA regions of H3K36me3-modified chromatin. Cryo-EM structures of maedi-visna virus intasomes saturated with LEDGF/p75 show that LEDGF/p75 remodels the target DNA binding surface of the intasome imposing steric strain that impedes engagement with the core nucleosomal regions. These findings elucidate the structural mechanism by which LEDGF/p75 directs lentiviral integration into linker DNA within H3K36me3-enriched chromatin.
This study provides compelling biochemical and structural evidence for how LEDGF/p75 modulates lentiviral integration site selection. The combination of in vitro reconstitution, long-read integration mapping, and high-resolution cryo-EM is particularly strong. Overall, the manuscript has substantial promise, but would benefit from further development in several key areas:
Major comments
1. Histones and array validation:
a. Though the authors use negative-stain EM and native PAGE (Fig. 1a–b) to validate their chromatin arrays, additional Mg^{2+}-dependent compaction data would help confirm that the Me0, Me3A, and Me3N arrays have comparable folding and accessibility.
b. Analytical data of the semi-synthetic H3K36me3 (native and mimic) should be included in the supplementary in order to be able to evaluate their quality.
c. While we appreciate the effort invested in generating a semi-synthetic histone, it is important to include a comparison with methylation at an alternative site (e.g., K4 or K27) to confirm that the observed differences are specific to K3me rather than a general effect of methylation.
d. What is the nucleosome occupancy of the lentiviral intasomes? It is not even clear what the size of this fragment is nor

how efficient and consistent the assembly was. Either way these results should be validated and compared with in vivo assembled fragments (native or transfected). Although the data show a clear shift toward linker DNA integration, some integration events still occur within nucleosome cores. The authors attribute these to partially unwrapped nucleosomes without direct evidence confirming array uniformity or nucleosome unwrapping. Validation of nucleosome positioning or compaction would help ensure that observe linker DNA preference reflects true biochemical selectivity rather than variation in chromatin assembly.
2. While the authors report normalized integration junctions, the absolute number of integration events remains unclear. The efficiency of integration in vitro versus in vivo should be quantified and discussed to provide proper context for the reported normalization. In addition, the authors should clarify whether there are absolute differences when using 601 versus native sequences as templates, as this distinction is mechanistically important- 601 represents an artificially strong nucleosome-positioning sequence, whereas native sequences are more dynamic.
3. Use of Oxford Nanopore sequencing to map integration junctions is a clear strength of the study, especially when paired with RT-qPCR as an orthogonal validation. It should be reported how sequencing depth and read quality were normalized between naked and chromatinized templates, since variation in Nanopore read recovery could affect the apparent integration patterns. Including a simple plot of read coverage or integration frequency per nucleosome repeat would make the analysis more complete and transparent.
4. Though the cryo-EM structures show near-saturated LEDGF occupancy (10–12 IBDs per intasome), the dose-dependent inhibition of integration into unmodified chromatin (Fig. 2b–c) remains underexplored. Quantifying how different levels of LEDGF occupancy influences integration selectivity through binding titrations or controlled stoichiometric reconstitutions would more directly connect the structural and biochemical data.
5. The cryo-EM structures provide new insight into how LEDGF/p75 decorates the intasome. To link these structural observations more directly to function, it would help to test whether specific IBD residues that form the negatively charged surface (Asp378, Glu379, and Glu414) drive the observed nucleosome-exclusion behavior. Mutations at these sites could be analyzed using the same long-read or RT-qPCR–based integration assays, or through dyad co-mapping on chromatinized yeast templates, to see whether they alter the linker DNA bias. Additionally, cryo-EM studies at lower LEDGF occupancy would be helpful to show how partial binding affects intasome conformation and target DNA accessibility.
6. The biochemical and structural data clearly show how LEDGF/p75 promotes integration into linker DNA within H3K36me3-modified chromatin, but it's not yet clear how these findings extend to a cellular context. To address this, the authors should perform integration-site profiling in a LEDGF/HRP2 knockout background complemented with wild-type LEDGF/p75 or variants lacking chromatin-binding ability (ΔPWWP, linker truncations) or carrying mutations in the IBD acidic patch and compare how these affect integration-site bias. In addition, they should compare these integration to models where the methyltransferase for H3K36me3 (SETD2), to confirm abrogation of integration.
Minor comments
1. More methodological detail would help improve clarity. For example, it would be useful to specify whether LEDGF/p75 was added stoichiometrically or in molar excess in the integration assays, as this information is important for interpreting the dose-dependent effects.
2. Statistical significance should be reported for all experiments. Although standard deviations are shown, P values are not provided and should be included to support the conclusions.
3. Overall, the manuscript would benefit from improved readability and general streamlining. There are also minor typographical errors that should be corrected.

Reviewer #5

(Remarks to the Author)
The authors present biochemical and structural results to interrogate HIV-1 integration into chromatinizined DNA, and the role of the host factor LEDGF. LEDGF appears to be critical for integration into chromatin enriched with H3K36me3, but not H3K36me0, which is expected. What is unexpected is that LEDGF seems to promote integration into linker DNA, and towards the center of a H3K36me3 poly-nucleosome array. This implies that the presence of neighboring nucleosomes, which should appear more frequently in the center of an array, facilitates integration, unexpectedly into linker DNA. They also present a structure of the LEDGF-saturated STC, through which they support the idea that LEDGF excludes bulky nucleosomes to facilitate integration into linker DNA. The manuscript is interesting and novel, and it begins to elucidate a mechanism of HIV-1 integration, which is clearly more complicated than what was initially thought in the field. While I envision that some of the ideas that the authors propose may be revised/adjusted as we learn more about HIV-1 integration (both with and without LEDGF), the current manuscript is clearly an important contribution. I would support publication, and my comments are mostly minor.

There are a few areas where the authors can improve the technical robustness. For example, integration into Me3A arrays is not shown in Figure 1e, but it is shown in the other panels. In Figure 2b, the authors test supplementation of Me3N with either dPWWP or WT LEDGF, but not with Me0 (by contrast, all 4 scenarios are shown in Figure 2c).

Why are there no +5.5 SHL integration events observed in Figure 3a, but some events observed in Figure 4b? Do the authors think this has to do with the increased malleability of more native chromatin. Could it also have to do with the low ratio of intasome:nucleosome in the fiber experiments, which would strongly disfavor selectivity of SHL +5.5?

The WT HIV intasomes that the authors assemble will form stacks. Some of these should be purified out using the TskGel ultra column. However, some stacks will remain, especially in the case of HIV. The authors should comment on whether the integration results (especially the selectivity of linker DNA and exclusion of core nucleosomes) depend on the extent of intasome purification, and whether intasome polydispersity can be a source of error.

It will be helpful to label the NCP / linker positions in Figure 3f, e.g., with shaded highlights.

The chromatinized yeast genomic DNA integration experiments are neat, but the results are a bit complicated. Integration into linker regions for HIV-1 and MVV is clear. However, the specific trends comparing Me0, Me3N, and Me3N+LEDGF are less clear, and these trends are also less consistent with the results of the fiber experiments. For example, integration into linker DNA of Me0 chromatin is seemingly more preferred than integration into linker DNA of Me3N chromatin. Also, addition of LEDGF does not seem to play a strong role in these experiments, unlike in the fiber experiments. The results here may be influenced by the complexity of the experiments, including natural malleability of yeast chromatin as well as intasome polydispersity (see comment above). I am not suggesting redoing these experiments. But I would like the authors to briefly acknowledge and comment on the possible sources of complications and error.

The inconsistent rotations in the presentation of the structural results in Figure 5 are a bit confusing.

LEDGF also has a chaperoning function, which does not depend on the PWWP domain. The authors should comment on the role of the chaperoning function of LEDGF, which is not discussed here.

Minor points:

“At larger even scales” should be “at even larger scales” (page 3). Widom601 should have a space

Is heterochromatin really considered inhospitable, such that integration into it would lead to failed infection (page 13)? One would think this may provide an opportunity for the virus to hide until activated.

Version 1:

Reviewer comments:

Reviewer #3

(Remarks to the Author)
My previous concerns have been addressed in the revised manuscript, and I have no further comments.

Reviewer #4

(Remarks to the Author)
1. Chromatin array comparability and compaction still need direct validation
The concern about whether the chromatin arrays actually fold, compact, and stay accessible in comparable ways wasn't really addressed head-on. The authors point to integration controls as indirect evidence of similar chromatin structure, but that's not the same as measuring Mg^{2+}-dependent compaction or running accessibility assays directly. Functional outcomes are useful, but they can't fully stand in for biophysical measurements, especially when the mechanistic story leans so heavily on differences in chromatin engagement.
2. The H3K36me3 specificity case still has a gap
The ask was to compare H3K36 methylation against another histone site, something like H3K4me3 or H3K27me3, and that experiment wasn't done. The authors' argument that LEDGF/p75 and PWWP domain dependence is enough to establish specificity is reasonable as far as it goes, but it doesn't rule out that other methylation states could produce similar effects on accessibility or integration behavior. Without an orthogonal methylation control, it's hard to be confident the effects are genuinely H3K36me3-specific rather than a more general consequence of histone methylation or shifts in chromatin structure.
3. Chromatin assembly quality is only partially characterized
The MNase digestion and AFM data are a step forward, but questions about nucleosome occupancy uniformity and possible partial unwrapping remain open. There's no quantitative demonstration that occupancy is consistent across templates, and the extent of nucleosome unwrapping isn't directly measured. This matters because the central claim, that LEDGF/p75 steers integration toward linker DNA, depends on being able to trust that linker accessibility isn't being shaped by variability in how the chromatin was assembled in the first place.
4. The "entropic filter" model is still more description than mechanism
The key experiment requested here was to mutate the acidic residues on the IBD surface and test whether they actually drive nucleosome exclusion. That wasn't done; it's been deferred to future work. Without it, the model stays correlative — the structural observations are interesting, but they don't demonstrate causality. Mutational data on those surface residues would be what moves this from a plausible structural story to a demonstrated mechanism.
5. How LEDGF occupancy shapes integration selectivity hasn't been tested
The rationale for using near-saturating LEDGF concentrations makes sense, but the question of what happens at lower, more variable occupancy levels was left unaddressed experimentally. Since the structural interpretation is built on highly occupied complexes, it would be worth knowing whether the linker-DNA preference holds up — or scales predictably — under conditions that better reflect the heterogeneity you'd expect in a cellular context. Titration or partial occupancy experiments would speak to that directly.
6. The cellular evidence

The ask was for experiments using chromatin-binding-deficient or structurally altered LEDGF variants to test integration-site selection in cells. The authors cite existing literature, which establish that the PWWP domain matters broadly, that's not in dispute. However, they weren't designed to test the specific model being proposed here about linker-DNA targeting and nucleosome exclusion. New cellular data would be needed to connect the in vitro mechanistic picture to what actually happens in cells.

Reviewer #6

(Remarks to the Author)
The authors responded to all critical comments of reviewer 1, however, a couple of responses are not addressed in the revision.

Comment 1c. LEDGF/p75 has been shown to affect the nucleosomal barrier to transcription (LeRoy et al, Sci Adv 2019)...
Comment 3. the PICs bound with LEDGF/p75

Open Access This Peer Review File is licensed under a Creative Commons Attribution 4.0 International License, which permits use, sharing, adaptation, distribution and reproduction in any medium or format, as long as you give appropriate credit to the original author(s) and the source, provide a link to the Creative Commons license, and indicate if changes were made.
In cases where reviewers are anonymous, credit should be given to 'Anonymous Referee' and the source.
The images or other third party material in this Peer Review File are included in the article's Creative Commons license, unless indicated otherwise in a credit line to the material. If material is not included in the article's Creative Commons license and your intended use is not permitted by statutory regulation or exceeds the permitted use, you will need to obtain permission directly from the copyright holder.
To view a copy of this license, visit https://creativecommons.org/licenses/by/4.0/

We thank all reviewers for their positive assessment of our work and their constructive criticisms, which helped us to improve our manuscript. The following is our point-by-point discussion of their comments.

Reviewer #1

The manuscript by Joshua Hope et al. presents exciting findings regarding target site selection of lentiviral intasomes in the context of chromatinized target DNA. The major claim of the study is that HIV and MVV integrate their genome into linker DNA rather than nucleosomal (wrapped) DNA. This finding contrasts results from earlier studies using less well-defined integrase-vDNA assemblies, and high resolution structural data of the well-defined prototype foamy virus intasome docked on a nucleosome as target capture complex and strand transfer complex. As the intasome core tetramer is quasi invariant along PFV and other characterized intasomes (MMTV, MVV, HIV), it was expected that nucleosomal DNA would be the preferred target independent of the retrovirus genus. Indeed, target DNA bending as required for aligning the scissile phosphodiester bonds in the intasome active site, could be (largely) offset by curvature induced via nucleosome wrapping. The observation that lentiviral MVV and HIV intasomes prefer linker DNA over nucleosomal DNA is thus surprising and an important advance. The question remains why lentiviral intasomes behave differently from the archetypical PFV intasome. In this manuscript, Hope and colleagues explain the shift away from the nucleosome core and into the linker DNA, by considering the structure (cryoEM) of MVV intasomes incubated with the host cofactor protein LEDGF/p75. In this structure, the interface of the intasome that faces the target DNA is decorated with several copies of the integrase-binding domain of LEDGF/p75 that would sterically clash with the nucleosome body.

The manuscript is well-written, with an extensive methods section and appropriate statistical analysis. I have the following questions and comments.

1. The role of chromatin structure is not clearly addressed.
a. The authors have used 12-mers of the W601 sequence interspersed with 44bp linker DNA, as well as sheared genomic DNA from budding yeast, for chromatin array reconstitution. W601 nucleosome positioning sequences wrap into nucleosomes with binding free energy lower by ~5 kBT with respect to strong natural 5S positioning sequence (Lowary and Widom, JMB 1998). Yeast chromatin is characterized by very short linker lengths (15-20 bp, Horz and Zachau JMB 1980) and has a more open configuration as compared to chromatin in mammals. In both cases, these thermodynamical and structural features are distinct from the chromatin naturally encountered by lentiviral PICs. Can the authors comment on how the current finding can be extrapolated to human chromatin?

Response: The main advantage of the Widom 601 array lies in the precise phasing of nucleosomes at predetermined positions. However, because the array comprises a defined DNA sequence, it is difficult to disentangle the effects of chromatin structure from local sequence preferences on integration. In addition, as the reviewer correctly notes, Widom 601 nucleosomes are considerably more stable than natural nucleosomes, raising the possibility that they may be less prone to transient unwrapping potentially required for integration at core nucleosome positions.

For these reasons, we complemented these experiments by examining integration into chromatin assembled on natural eukaryotic DNA. It is important to note that the chromatin was assembled using *human* histones; we deliberately avoid referring to these substrates as “yeast chromatin,” as our intention was not to recreate *in vivo*-like yeast chromatin. In principle, these

experiments could be done with any DNA. Compared with mammalian DNA, yeast DNA offers practical advantages, including high mappability of integration sites. It also allowed us to validate our dyad mapping approach by correlating the dyads with those observed in an independent study.

The revised manuscript includes AFM analysis of chromatin assembled on yeast DNA. The imaging revealed a broad distribution of linker lengths (Fig. S6b in the revised manuscript), confirming that the nucleosomal arrays are structurally heterogeneous.

Crucially, the results obtained with Widom 601 arrays and chromatinized yeast DNA are highly consistent. In both systems, when linker lengths approximate those typical of human chromatin (44 bp in the Widom 601 arrays) or when nucleosomes are naturally flexible (variable linkers in yeast DNA), lentiviral intasomes preferentially integrate into linker DNA while avoiding nucleosome core regions.

To clarify this point, we added the following statement to the revised manuscript (Discussion, line 380-383): “Importantly, irrespective of whether nucleosomes were assembled on highly defined W601 arrays or more diversely distributed on yeast DNA, lentiviral intasomes strongly disfavoured the bodies of nucleosomes and instead targeted the linker DNA, while PFV integration was consistently observed within the nucleosome core.”

b. The chromatin reconstituted on sheared yeast genomic DNA is not assessed for quality. The assay that uses the Fenton reaction combined with integration site sequencing is very elegant and establishes a ruler with respect to the nucleosome dyad. Nevertheless, nucleosome density arguably plays a role with regards to e.g. linker DNA bending. Here, a single molecule (i.e. Nanopore) sequencing or imaging (for example EM) approach would be useful.

Response: Thank you for these suggestions. Revised manuscript includes validation of nucleosome wrapping with H3K36Me0 and H3K36Me3 modified histones by micrococcal nuclease digestion and by atomic force microscopy (Fig. S6a,b).

c. LEDGF/p75 has been shown to affect the nucleosomal barrier to transcription (LeRoy et al, Sci Adv 2019), suggesting that it can have (an additional) role in remodeling nucleosomes and chromatin making the target either more or less accessible.

Response: We have carefully considered the data presented by LeRoy et al. The authors did not see any difference in the effect of LEDGF/p75 between unmodified and H3K36Me3 chromatin. Furthermore, a 6-9 fold molar excess of LEDGF over nucleosomes was required to have appreciable chaperoning activity. Because our assays were performed at an LEDGF:nucleosome molar ratio of 1.3:1 and 2.6:1, we expect nucleosome destabilisation by LEDGF/p75 to be a minor effect.

2. The cryo-EM data is recorded on samples of Maedi Visna Virus intasomes in the presence of 0.25 mg/mL or approximately 4 micromolar of LEDGF/p75 (MW 62 kDa). This is orders of magnitude higher as compared to that compared to integration assays (8-16 nM). What is the logic to saturate LEDGF binding pockets beyond what might be relevant for the biological effect? How does this compare to the concentration of LEDGF/p75 in the cell?

Response: Thank you for raising this important issue. LEDGF/p75 is one of the most abundant proteins in the cell. For example, quantitative analysis of $CD4^+$ T cells (Howden et al., Nature

Immunol, 2019; PMID: 31591570) found >200,000 copies of LEDGF/p75/PSIP1 per cell, corresponding to ~1.5 µM overall intracellular concentration. Remarkably, this copy number corresponds to ~1% histone H2A level. However, bulk of LEDGF/p75 is found in the nucleus, bound to chromatin, where it potentially forms condensates (Scoca et al, JMBC, 2022; PMID 36314049 and Batisse at al, JBC, 2024; PMID 3876218). Therefore, the local concentration at the sites of PIC uncoating and integration is likely considerably higher than the global estimate of 1.5 µM. Therefore, our input of LEDGF/p75 when soaking immobilized intasomes is justified.

This point is now addressed in the manuscript (Results, lines 290-294): "LEDGF/p75 is a highly abundant protein, present in CD4+ T cells at an average intracellular concentration of 1-2 µM [82]. The bulk of this protein is concentrated in the nucleus, bound to chromatin 83, where it potentially forms condensates [84,85]. Therefore, local concentration of LEDGF/p75 in the vicinity of chromatin - where HIV-1 PICs are released from their capsid cores [86] - is likely substantially higher."

It is also important to note that the strand transfer reactions used diluted chromatin, and proportionally diluted LEDGF/p75. The protein tends to form co-precipitates with DNA and chromatin and causes aggregation of the intasomes, likely due to its propensity to form condensates.

3. The authors claim that the PICs remain bound with LEDGF/p75, and refer to Llano et al., J. Virol. 2004 to substantiate this claim. However, whereas Llano et al. used cytoplasmal PIC fractions, here the authors use crude nuclear PIC extract.

Response: LEDGF/p75 is predominantly found in the nucleus, as shown in several publications, including Fig. 4 of Llano et al., J. Virol, 2004. The fact that LEDGF/p75 was detected bound to HIV-1 PICs extracted from cell cytoplasms, only strengthens the notion that the PICs bind this host factor with high affinity.

Most importantly, Fig1c shows that integration preference of our PICs for H3K36Me3 arrays is recovered by extracts from HEK-293T cells but not LEDGF/p75/HRP2 knockout cells.

4. On line 366, the authors write: "we propose that multiple LEDGF/p75 molecules associated with the intasome function as an entropic filter". It is not clear what the authors mean with "entropic filter". Does the multivalency of PWWP readers associated with the intasome reduce its possible degrees of freedom, or does the multivalency warrant integration in K36Me3 rich genomic areas, "filtering out" epigenetic noise?

Response: Thank you for this comment (see also our answer to Reviewer 3 Q1). In our model, both rigidly bound IBDs and flexibly linked PWWP domains extending from the intasome obstruct the approach to the intasomal active sites. Bulky and conformationally constrained chromatin segments would be generally disfavoured, compared to more flexible naked DNA. However, the interaction of the PWWP domains with H3K36Me3 nucleosomes would trap the modified chromatin segment, allowing it to linger sufficiently long for productive engagement. This is why we refer to the mechanism as entropic filter. The concept is somewhat related to the mechanism of nuclear entry: the interaction of disordered FG repeats with karyopherins allows their entry into and passage through the nuclear pore.

We clarified the hypothesis in the revised manuscript (Discussion, last paragraph, lines 392-400): “We propose that multiple LEDGF/p75 molecules associated with the intasome function as an entropic filter by restricting the set of chromatin conformations and approach trajectories that can lead to productive engagement with the intasome active sites. In this model, access by bulky chromatin segments is generally obstructed. However, H3K36Me3-containing chromatin segments, entrapped through interactions with PWWP domains, can linger long enough to permit productive engagement. This model also explains why more flexible, nucleosome-free linker DNA segments can access the active sites in the presence of LEDGF/p75 (Figs 3c and 4b). Conceptually, this mechanism resembles nuclear pore entry, where cargoes are licensed through productive interactions with nucleoporin FG repeats [96].”

We also stated that the hypothesis will need further experimental validation (same paragraph, line 403): “However, future studies will be required to test the entropic filter hypothesis.”

5. On line 360, it is stated that “the disordered region between PWWP and IBD is a predicted average of ~80 nm”. Assuming the disordered region comprises 255 amino acids and taking the length per amino acid 0.38 nm, the contour length of the disordered region is ~97 nm. However, the disordered region is clearly not perfectly and fully extended. Predicting the extension of the region as a worm-like chain with bending persistence length 0.4 nm gives and end-end distance of 7.2 nm (see Kjaergaard et al., Methods in Enzymology, 2021). Similarly models that consider self-avoidance (self-avoiding walk) and include sequence features (ALBATROSS; Lotthammer et al., Nature Methods 2024) give mean values for the end-end distance of ~10nm and 12.4 nm. Extensions beyond 30 nm are extremely uncommon.

Response: Thank you for this comment. We updated the manuscript with an average end-to-end distance between the domains is predicted to be 12 nm by ALBATROSS.

We amended the manuscript accordingly (Discussion, lines 385-389): “The disordered region between the PWWP domain and the IBD in LEDGF/p75 comprises 255 amino acid residues, predicted to span an average distance of ~12 nm [95] or ~90 nm when fully outstretched. Considering the linker DNA length of 10-20 nm [63], the ~20-nm lentiviral intasome could plausibly engage several nucleosomes via multiple copies of LEDGF/p75.”

Together, the above comments indicate that the manuscript would be suitable for publication in Nature Communications with relatively minor changes to the submitted version.

Reviewer #3

Hope et al. show that lentiviral integrases have a strong bias to integrate into the linker DNA regions within H3K36Me3-enriched chromatin because, while lentiviral intasomes disfavor core nucleosomes, LEDGF/p75 recruits them to H3K36Me3N-containing polynucleosomal arrays. This is high-quality work that provides novel insights into the mechanism of replication of important viral pathogens. I would suggest clarification on the following aspect, though.

The authors state that

LEDGF/p75 alters the target DNA binding platform of the lentiviral intasome, imposing significant constraints on the path and configuration of target DNA to impede nucleosome engagement.

The cryo-EM structures reveal that bound IBDs remodel the tDNA binding platform of the intasome, acting as physical barriers to facilitate optimal linker DNA engagement for integration, while excluding the approach of more bulky core nucleosome particles.

LEDGF/p75 IBDs reshape the tDNA binding platform of the lentiviral intasomes, while multiple LEDGF/p75 molecules associated with the intasome function as an entropic filter, excluding H3K36Me0 nucleosomes.

These claims appear to be very hand-wavy.

Is the target DNA or the target DNA-binding platform actually shaped differently in the new structure compared to the previous STC structures determined with a less-saturating amount of LEDGF/p75 (besides having more LEDGF/p75 molecules bound)?

Response: Thank you for raising this important point and highlighting the ambiguity. When already bound, target DNA is in the same conformation whether the IBDs (along with flexibly linked PWWP domains) are present or not. In our model, it is the approach to the active sites that is obstructed; so the "filtering" would occur before productive engagement, at the level of allowed approach pathways. Therefore, entropic filter is an appropriate term.

We clarified these points in the revised manuscript (Discussion, lines 392-400): "We propose that multiple LEDGF/p75 molecules associated with the intasome function as an entropic filter by restricting the set of chromatin conformations and approach trajectories that can lead to productive engagement with the intasome active sites. In this model, access by bulky chromatin segments is generally obstructed. However, H3K36Me3-containing chromatin segments, entrapped through interactions with PWWP domains, can linger long enough to permit productive engagement. This model also explains why more flexible, nucleosome-free linker DNA segments can access the active sites in the presence of LEDGF/p75 (Figs 3c and 4b). Conceptually, this mechanism resembles nuclear pore entry, where cargoes are licensed through productive interactions with nucleoporin FG repeats [96]."

It is not clear how LEDGF/p75 selectively interferes with the binding of DNA in nucleosomes. To support the entropic filter hypothesis, the authors might want to test if IBD by itself reduces integration into unmodified chromatin, similarly to the full-length LEDGF/p75 (Fig. 1e).

Response: The entropic filter is a speculative idea at this stage. Both the rigidly bound IBDs and the flexibly tethered PWWP domains likely contribute to this mechanism. Follow up work will be required to validate this hypothesis (Discussion, final paragraph): "However, future studies will be required to test the entropic filter hypothesis."

In addition, the authors should estimate the PIC or intasome concentration used in the in vitro assays. In Fig. 1, 2 nM of LEDGF/p75 is saturating, whereas in Fig. 2, 32 nM is still not saturating. Is the discrepancy due to a difference in the intasome concentration?

Response: Taking raw number of integration junctions into naked DNA, intasomes are in approximately 100-1000 fold excess to the PICs used in the assays.

We amended the manuscript accordingly (Results, lines 151-154): "Although we could not directly quantify the efficiency of intasome assembly, the maximal intasome concentration was

0.025 nM, ensuring that tDNA was in large excess. Nonetheless, intasomes generated approximately 100 - 1,000-fold more integration junctions than native HIV-1 PICs, reflecting the scarcity of the latter."

Reviewer #4

In this manuscript, Hope and colleagues investigate how the transcriptional co-activator LEDGF/p75 guides lentiviral integration within chromatin. Using native HIV-preintegration complexes and in vitro assembled lentiviral intasomes with defined chromatinized templates, the authors show that LEDGF/p75 promotes integration in linker DNA regions of H3K36me3-modified chromatin. Cryo-EM structures of maedi-visna virus intasomes saturated with LEDGF/p75 show that LEDGF/p75 remodels the target DNA binding surface of the intasome imposing steric strain that impedes engagement with the core nucleosomal regions. These findings elucidate the structural mechanism by which LEDGF/p75 directs lentiviral integration into linker DNA within H3K36me3-enriched chromatin.
This study provides compelling biochemical and structural evidence for how LEDGF/p75 modulates lentiviral integration site selection. The combination of in vitro reconstitution, long-read integration mapping, and high-resolution cryo-EM is particularly strong. Overall, the manuscript has substantial promise, but would benefit from further development in several key areas:
Major comments

1. Histones and array validation:
a. Though the authors use negative-stain EM and native PAGE (Fig. 1a–b) to validate their chromatin arrays, additional Mg^{2+}-dependent compaction data would help confirm that the Me0, Me3A, and Me3N arrays have comparable folding and accessibility.

Response: We will argue that outcomes of integration control reactions assuage concerns for additional validation of the utilized arrays. The preference for native PICs to target $Me3_N$-containing arrays is lost when the PICs were made from LHKO (LEDGF/HRP2) double knockout cells (Fig. 1c). Similarly, the preferences for HIV-1 and MVV intasomes to integrate into the $Me3_N$-containing chromatin was lost when LEDGF/p75 was substituted for the N-terminal ΔPWWP deletion mutant lacking the H3K36Me3-reader domain. Should there be major discrepancies in chromatin structure between the array methylation status, this would have been apparent in integration assays set up with these critical negative controls.

b. Analytical data of the semi-synthetic H3K36me3 (native and mimic) should be included in the supplementary in order to be able to evaluate their quality.

Response: Thank you for this suggestion. We included validation of our native $H3K36Me3_N$ protein by LC-MS in the revised manuscript (Fig. S1b).

The MLA variant was only used in the early experiments in our study (Figs 1c, and 2a). Cys alkylation by Bromoethyltrimethylammonium is a highly efficient reaction. Accordingly, $H3K36Me3_A$ was confirmed to be fully modified. A paper copy of LC-MS results from 2017 is shown in Appendix 1, at the end of this document. The molecular weight of the major component A corresponds, within the tolerance range (~0.05%), to the expected product. The minor secondary peak B was interpreted as partial decomposition of the trimethylammonium substituent. Importantly, no trace of unmodified protein was detected. While preparing this rebuttal, we identified a sample of H3K36cMe3 histone octamer that was used in Figs 1c and

2a and stored at -70ºC since then (accumulated life of the histone $H3K36Me3_A$ is ~9 years). We dissociated the octamer into the individual subunits and analysed them by LC-MS. Although the results revealed that during the prolonged storage, H2B and $K36Me3_A$ underwent partial oxidation, no traces of non-modified H3 or its oxidized or acetonitrile adducts were detected (Appendix 2).

c. While we appreciate the effort invested in generating a semi-synthetic histone, it is important to include a comparison with methylation at an alternative site (e.g., K4 or K27) to confirm that the observed differences are specific to K3me rather than a general effect of methylation.

Response: We show that effect of H3K36Me3 on HIV-1 integration is dependent on the presence of LEDGF/p75 and the PWWP domain (Fig. 1c, Fig. 2a-c, Fig S3a). The only reasonable interpretation is that the effect is specific.

NB: Unmodified histone is a generally accepted negative control; see for example: Lauberth et al, Cell, 2013 (PMID: 23452851); Chen et al, ChemBioChem, 2014 (PMID: 25155436); Nitsch et al, Mol. Cell, 2026 (PMID: 41421336).

d. What is the nucleosome occupancy of the lentiviral intasomes? It is not even clear what the size of this fragment is nor how efficient and consistent the assembly was. Either way these results should be validated and compared with in vivo assembled fragments (native or transfected). Although the data show a clear shift toward linker DNA integration, some integration events still occur within nucleosome cores. The authors attribute these to partially unwrapped nucleosomes without direct evidence confirming array uniformity or nucleosome unwrapping. Validation of nucleosome positioning or compaction would help ensure that observe linker DNA preference reflects true biochemical selectivity rather than variation in chromatin assembly.

Response: In our manuscript, we discuss chromatinization of target DNA that intasomes and preintegration complexes integrate into. Our work does not address nucleosome infiltration of intasomes/PICs (viral DNA), which is a topic for a separate study.

The PFV intasome control reproduced previously reported preference for SHL ±3.5 positions both with regular Widom 601 array and chromatinized yeast DNA, validating our arrays. Note also the striking differences between naked and chromatinized target DNA conditions, again functionally confirming chromatinization. Spiking of integration reactions into Windom 601 arrays with naked PhiX174 DNA (Fig. 3e) also confirms the propensity of the intasome to select naked DNA structures, over unmodified chromatinized structures.

Thank you for highlighting the ambiguity in our description of chromatinized yeast DNA samples. In Methods, we explained that the DNA was sheared by digestion with *Xba*I and *Nhe*I. Accordingly, the mean and median fragment sizes were 3 and 2 kb, respectively. The revised manuscript states (Methods, lines 544-547): "Yeast genomic DNA, isolated from *Saccharomyces cerevisiae* strain BY4741 (ref. 100) using YeaStar Genomic DNA kit (Zymo Research), was sheared by digestion with restriction enzymes *Xba*I and *Nhe*I (New England Biolabs) in the presence of 0.1 mg/ml RNase A (Qiagen) to generate DNA fragments with a median size of ~2 kb."

In addition, the revised manuscript includes results confirming wrapping of yeast DNA by micrococcal nuclease digestion and atomic force microscopy (Fig. S6a and b).

2. While the authors report normalized integration junctions, the absolute number of integration events remains unclear. The efficiency of integration in vitro versus in vivo should be quantified and discussed to provide proper context for the reported normalization. In addition, the authors should clarify whether there are absolute differences when using 601 versus native sequences as templates, as this distinction is mechanistically important- 601 represents an artificially strong nucleosome-positioning sequence, whereas native sequences are more dynamic.

Response: Absolute quantification of PIC integration *in vivo* is not currently feasible. Only minority of HIV-1 vDNA copies synthetized during infection would assemble into PICs, and only some of these PICs would integrate into host chromatin. The reasons for this apparent inefficiency remain unclear. Furthermore, PICs are extremely scarce, transiently present in a single copy (or several copies at high multiplicity of infection) per infected cell.

In this work, we solely focused on how lentiviral intasomes are affected by a nearby nucleosome. Taken together, our results obtained with both Widom 601 arrays and chromatin assembled on natural DNA converge to the same conclusion: lentiviral intasomes preferentially target linker DNA and avoid nucleosome core regions. Relative preferences between different types of chromatin will be subject for a separate study.

3. Use of Oxford Nanopore sequencing to map integration junctions is a clear strength of the study, especially when paired with RT-qPCR as an orthogonal validation. It should be reported how sequencing depth and read quality were normalized between naked and chromatinized templates, since variation in Nanopore read recovery could affect the apparent integration patterns. Including a simple plot of read coverage or integration frequency per nucleosome repeat would make the analysis more complete and transparent.

Response: Thank you for this comment. We have now deposited the Nanopore data with NCBI GEO (GSE310967; reviewer token: kbcdcusslnkbtsz) and Widom 601 data is deposited in Zenodo (https://doi.org/10.5281/zenodo.17566487). In addition, new Supplementary Table S1 details numbers of mapped unique reads from each sample of all experiments and information on read counts for all Nanopore data are given in the supplied Source Data file.

4. Though the cryo-EM structures show near-saturated LEDGF occupancy (10–12 IBDs per intasome), the dose-dependent inhibition of integration into unmodified chromatin (Fig. 2b–c) remains underexplored. Quantifying how different levels of LEDGF occupancy influences integration selectivity through binding titrations or controlled stoichiometric reconstitutions would more directly connect the structural and biochemical data.

Response: Thank you for raising this important issue (see also our response to Reviewer 1, Q2). LEDGF/p75 is one of the most abundant proteins in the cell; its average intracellular concentration was estimated to be ~1.5 µM, while local concentration in the nucleus and around chromatin is considerably higher. Our results show that low µM LEDGF/p75 is sufficient to achieve near complete saturation of the intasome. Thus, our cryo-EM experiments were done in the biologically relevant regime; the intasomes were spread on an affinity grid and exposed to 4 µM LEDGF/p75. This point is now addressed in the manuscript (Results, lines 290-294): "LEDGF/p75 is a highly abundant protein, present in CD4+ T cells at an average intracellular concentration of 1-2 µM [82]. The bulk of this protein is concentrated in the nucleus, bound to chromatin [83], where it potentially forms condensates [84,85]. Therefore, local concentration of

LEDGF/p75 in the vicinity of chromatin - where HIV-1 PICs are released from their capsid cores [86] - is likely substantially higher."

5. The cryo-EM structures provide new insight into how LEDGF/p75 decorates the intasome. To link these structural observations more directly to function, it would help to test whether specific IBD residues that form the negatively charged surface (Asp378, Glu379, and Glu414) drive the observed nucleosome-exclusion behaviour. Mutations at these sites could be analyzed using the same long-read or RT-qPCR–based integration assays, or through dyad co-mapping on chromatinized yeast templates, to see whether they alter the linker DNA bias. Additionally, cryo-EM studies at lower LEDGF occupancy would be helpful to show how partial binding affects intasome conformation and target DNA accessibility.

Response: Positions of these residues in the structure are intriguing and conserved in the other IBD-containing protein, HRP-2. Along with the bulk of rigidly bound IBDs, and flexibly tethered PWWP domains, they may contribute to the entropic filter. Testing this hypothesis will require a separate dedicated effort. We make a note in revised Discussion (line 403) "However, future studies will be required to test the entropic filter hypothesis."

6. The biochemical and structural data clearly show how LEDGF/p75 promotes integration into linker DNA within H3K36me3-modified chromatin, but it's not yet clear how these findings extend to a cellular context. To address this, the authors should perform integration-site profiling in a LEDGF/HRP2 knockout background complemented with wild-type LEDGF/p75 or variants lacking chromatin-binding ability (ΔPWWP, linker truncations) or carrying mutations in the IBD acidic patch and compare how these affect integration-site bias. In addition, they should compare these integration to models where the methyltransferase for H3K36me3 (SETD2), to confirm abrogation of integration.

Response: The importance of the LEDGF/p75 PWWP domain for HIV-1 integration has been addressed in an early knockout study (Shun et al., Genes Dev., 2007; PMID: 17639082). Furthermore, replacement of the PWWP domain with alternative chromatin-binding modules was shown to redirect HIV-1 integration in cells (Ferris et al., PNAS, 2010; PMID: 20133638), supporting its role in targeting lentiviral integration. We added the following to the revised manuscript (Introduction, lines 84-86): "Moreover, replacement of the LEDGF/p75 PWWP domain with alternative chromatin reader domains is sufficient to redirect HIV-1 integration to genomic regions enriched in the corresponding histone modifications [47]."

Although LEDGF/p75 has a strong effect on HIV-1 integration targeting, it is not strictly required for HIV-1 infectivity (see, for example, Shun et al., Genes Dev., 2007; PMID: 17639082). Therefore, it is not unexpected that the effects of SETD2 ablation are nuanced albeit significant (Pathak et al., mBio, 2026; PMID: 41778782). Revised Introduction includes a reference to Pathak et al. (lines 82-84): "Ablation of SETD2, the methyltransferase responsible for trimethylation of histone H3 Lys36, similarly redistributes HIV-1 integration sites toward upstream gene regions [46]."

Future studies will be required to disentangle the potential roles of LEDGF/p75 as a chromatin tether and as an entropic filter influencing chromatin engagement.

Minor comments

1. More methodological detail would help improve clarity. For example, it would be useful to specify whether LEDGF/p75 was added stoichiometrically or in molar excess in the integration assays, as this information is important for interpreting the dose-dependent effects.

Response: Thank you for highlighting this omission. Nucleosomes were present at large molar excess to the intasome, to avoid multiple integration events into a single chromatin segment and to best mimic the biological system. Concentrations of LEDGF/p75 ranged from sub- to super-stoichiometric amounts relative to H3K36Me3 binding sites. Stoichiometry is clarified in the revised manuscript; the final concentrations are now given for all experiments with poly-nucleosomal arrays.

2. Statistical significance should be reported for all experiments. Although standard deviations are shown, P values are not provided and should be included to support the conclusions.

Response: The revised manuscript contains a table reporting statistical analyses for all experiments given in the Source Data spreadsheet.

3. Overall, the manuscript would benefit from improved readability and general streamlining. There are also minor typographical errors that should be corrected.

Response: We hope the revised version is free of typographical errors.

Reviewer #5:

The authors present biochemical and structural results to interrogate HIV-1 integration into chromatinizined DNA, and the role of the host factor LEDGF. LEDGF appears to be critical for integration into chromatin enriched with H3K36me3, but not H3K36me0, which is expected. What is unexpected is that LEDGF seems to promote integration into linker DNA, and towards the center of a H3K36me3 poly-nucleosome array. This implies that the presence of neighboring nucleosomes, which should appear more frequently in the center of an array, facilitates integration, unexpectedly into linker DNA. They also present a structure of the LEDGF-saturated STC, through which they support the idea that LEDGF excludes bulky nucleosomes to facilitate integration into linker DNA. The manuscript is interesting and novel, and it begins to elucidate a mechanism of HIV-1 integration, which is clearly more complicated than what was initially thought in the field. While I envision that some of the ideas that the authors propose may be revised/adjusted as we learn more about HIV-1 integration (both with and without LEDGF), the current manuscript is clearly an important contribution. I would support publication, and my comments are mostly minor.

There are a few areas where the authors can improve the technical robustness. For example, integration into Me3A arrays is not shown in Figure 1e, but it is shown in the other panels. In Figure 2b, the authors test supplementation of Me3N with either dPWWP or WT LEDGF, but not with Me0 (by contrast, all 4 scenarios are shown in Figure 2c).

Response: Relatively early in the project (year 2018), we realised that Me3A was inferior to Me3N (as shown in Fig. 1c and Fig. 2a). This observation was consistent with excellent studies by Ruthenburg and colleagues (Chen et al., ChemBioChem, 2014, PMID 25155436; Chen et al., Biochemistry, 2018, PMID 29111671). Remarkably, Chen et al. observed that Me3N pulled down LEDGF from human cell extracts far more robustly than Me3A (see Fig. 3A in PMID

25155436). We spent over a year to optimize production of native material and used it since for all experiments. Me3A is present only in Fig. 1c and Fig. 2a, which were foundational for the rest of the study. The results showed that we can reproduce preferred HIV-1 PIC and HIV-1 and MVV intasome integration into Me3 chromatin *in vitro*.

Me0 is absent in Fig. 2b, because the plot shows data normalised to Me0. Me3A was not used in Fig. 1e, because we failed to observe strong effects of the analogue, as shown in Fig. 2a.

Why are there no +5.5 SHL integration events observed in Figure 3a, but some events observed in Figure 4b? Do the authors think this has to do with the increased malleability of more native chromatin. Could it also have to do with the low ratio of intasome:nucleosome in the fiber experiments, which would strongly disfavor selectivity of SHL +5.5?

Response: In the original 2015 study, we did not detect integration at SHL ±5.5 by electrophoresis of strand transfer products (for example, see lane 2 of Fig. 1a in PMID 26061770). Nanopore sequencing is far more sensitive than the agarose gels we used in 2015; for example, the histogram shown in Fig. 4b is an aggregate from over 41,000 integration-fragmentation events (PFV, Me0 condition).

It is important to note, as we explained in the manuscript, the new method has an inherent bias against shorter fragments, which is indicated by the slope of the grey curve (PFV, Naked condition). Therefore, Fig. 4b may exaggerate the frequency of SHL ±5.5 events.

By contrast to a wide variety of nucleosomes used in Fig. 4, data in Fig. 3 are based on counting integration events into a single defined DNA sequence. A more “troubling” observation is the sheer absence of the SHL -3.5 site, while SHL +3.5 events are strong. This striking asymmetry results from asymmetry of the Widom 601 DNA sequence and inherent preferences of intasomes for local sequence. In fact, we took advantage of this property in Maskel et al 2015: our D02 nucleosome bound PFV intasome asymmetrically, which helped us to refine the structure of the TCC and later the STC without averaging the nucleosome component. So, the answer to the question why we do not observe minor SHL ±5.5 events in Fig. 3 is that integration at this minor (ergo less optimal) site requires more optimal local geometry/sequence of the tDNA duplex.

The WT HIV intasomes that the authors assemble will form stacks. Some of these should be purified out using the TskGel ultra column. However, some stacks will remain, especially in the case of HIV. The authors should comment on whether the integration results (especially the selectivity of linker DNA and exclusion of core nucleosomes) depend on the extent of intasome purification, and whether intasome polydispersity can be a source of error.

Response: HIV-1 intasomes are infamously polydisperse. This is precisely why we included the MVV intasome, which forms as relatively monodisperse 16-mer species (see Fig. 1 and Fig. S5 in Cook et al., 2020; PMID 32001525). Crucially, the results are largely consistent between these two lentiviral intasomes, while sharply contrasting the PFV control. We have added the reasoning behind opting to include MVV intasomes to the revised manuscript (Results, lines 147-149): “We included the MVV intasome in key experiments alongside the HIV-1 intasome for comparison, as MVV readily forms discrete, homogeneous complexes *in vitro*, whereas HIV-1 intasomes are comparatively polydisperse [8,9,70].”

It will be helpful to label the NCP / linker positions in Figure 3f, e.g., with shaded highlights.

Response: Thank you for this comment. We revised Fig. 3 as requested, which made it more clear and striking.

The chromatinized yeast genomic DNA integration experiments are neat, but the results are a bit complicated. Integration into linker regions for HIV-1 and MVV is clear. However, the specific trends comparing Me0, Me3N, and Me3N+LEDGF are less clear, and these trends are also less consistent with the results of the fiber experiments. For example, integration into linker DNA of Me0 chromatin is seemingly more preferred than integration into linker DNA of Me3N chromatin. Also, addition of LEDGF does not seem to play a strong role in these experiments, unlike in the fiber experiments. The results here may be influenced by the complexity of the experiments, including natural malleability of yeast chromatin as well as intasome polydispersity (see comment above). I am not suggesting redoing these experiments. But I would like the authors to briefly acknowledge and comment on the possible sources of complications and error.

Response: These are fair points. We discussed these subtle differences in revised manuscript (Results, lines 271-282): "Interestingly, compared to non-modified chromatin, H3K36Me3-containing nucleosomes were slightly more receptive for lentiviral integration, allowing more events within entry/exit regions ($p < 10^{-323}$). This observation is consistent with recent findings that H3K36Me3 promotes nucleosome breathing, facilitating transient unwrapping of the outer DNA segments [80,81]. The effect was likely masked in our experiments using W601 arrays due to local tDNA sequence preferences of the intasomes. Supplementation of HIV-1 intasomes with 8 nM LEDGF/p75 did not result in appreciable shifts in integration site distributions on H3K36Me3 chromatin, whereas MVV integration sites shifted further away from the dyad. One possible explanation is that the greater heterogeneity of HIV-1 intasome preparations relaxes structural constraints, permitting increased integration into nucleosome core regions upon LEDGF/p75 saturation. Nonetheless, these results confirm and extend our observations from the W601 arrays: in contrast to PFV, lentiviral intasomes integrate predominantly outside nucleosome core particles."

The inconsistent rotations in the presentation of the structural results in Figure 5 are a bit confusing.

Response: One of the key roles of Fig. 5 is to illustrate accumulation of LEDGF on one side of the intasome. At the same time, we wanted to avoid duplicating the view of the posterior side, which is identical between the two structures. We would like to keep the original design, which optimises the use of space.

LEDGF also has a chaperoning function, which does not depend on the PWWP domain. The authors should comment on the role of the chaperoning function of LEDGF, which is not discussed here.

Response: Same answer as to Reviewer 1 Q1c: We have carefully considered the data presented by LeRoy et al. The authors did not see any difference in the effect of LEDGF/p75 between unmodified and H3K36Me3 chromatin. Furthermore, a 6-9 fold molar excess of LEDGF over nucleosomes was required to have appreciable chaperoning activity. Because our assays were performed at an LEDGF:nucleosome molar ratio of 1.3:1 and 2.6:1, we expect nucleosome destabilisation by LEDGF/p75 to be a minor effect.

Minor points:

“At larger even scales” should be “at even larger scales” (page 3). Widom601 should have a space

Response: OK, thank you.

Is heterochromatin really considered inhospitable, such that integration into it would lead to failed infection (page 13)? One would think this may provide an opportunity for the virus to hide until activated.

Response: This is a point for interesting discussion. It is true that a population of silent proviruses contributes to persistence, especially in the context of antiretroviral therapy. We would argue that robust proviral expression is required for transmission (to maintain sufficient viral load in the current host and to initiate infection of a new host). After all, viral promoters are among the strongest known. Conversely, heterochromatinization is a defence mechanism against transposable elements, and herpesviruses are silenced by heterochromatinization.

```
Spectrum Deconvolution
Data File C:\HPCHEM\1\DATA\SEP17\26091704.D          Report hH3.1 K36c me3

=====================================================================
Injection Date    : 26/09/2017 14:19:36 PM          Seq. Line :    2
Sample Name       :                                  Location : Vial 4
Acq. Operator     : peptide chemistry                     Inj :    1
                                                   Inj Volume : 3 µl
Acq. Method       : C:\HPCHEM\1\METHODS\APSYMET\AAFPSTD.M
Last changed      : 20/09/2017 15:43:40 PM by peptide chemistry
Analysis Method   : C:\HPCHEM\1\METHODS\APSYMET\AAFPSTD.M
Last changed      : 26/09/2017 14:35:18 PM by peptide chemistry
                    (modified after loading)
hydrophobic peptide fast gradient 5%B- 100%B over 8.5 mins with std wavelengths

=====================================================================
                     Deconvolution Parameters
=====================================================================

Adduct Ion(Positive): +H, 1.0079 Da
Adduct Ion(Negative): -H, -1.0079 Da
Low MW:               1000
High MW:              20000
Maximum Charge:       50
Minimum Peaks in Set: 3
Retain Residual:      No
Ion PWHH:             0.6 Da
MW Agreement:         0.05 %
Noise Cutoff:         1000 counts
Abundance Cutoff:     10 %
MW Assign:            Curve fit
MW Assign Cutoff:     40 %
Envelope Cutoff:      50 %

Deconvolution of Spectrum #    1 @   9.098 - 9.352 min
```

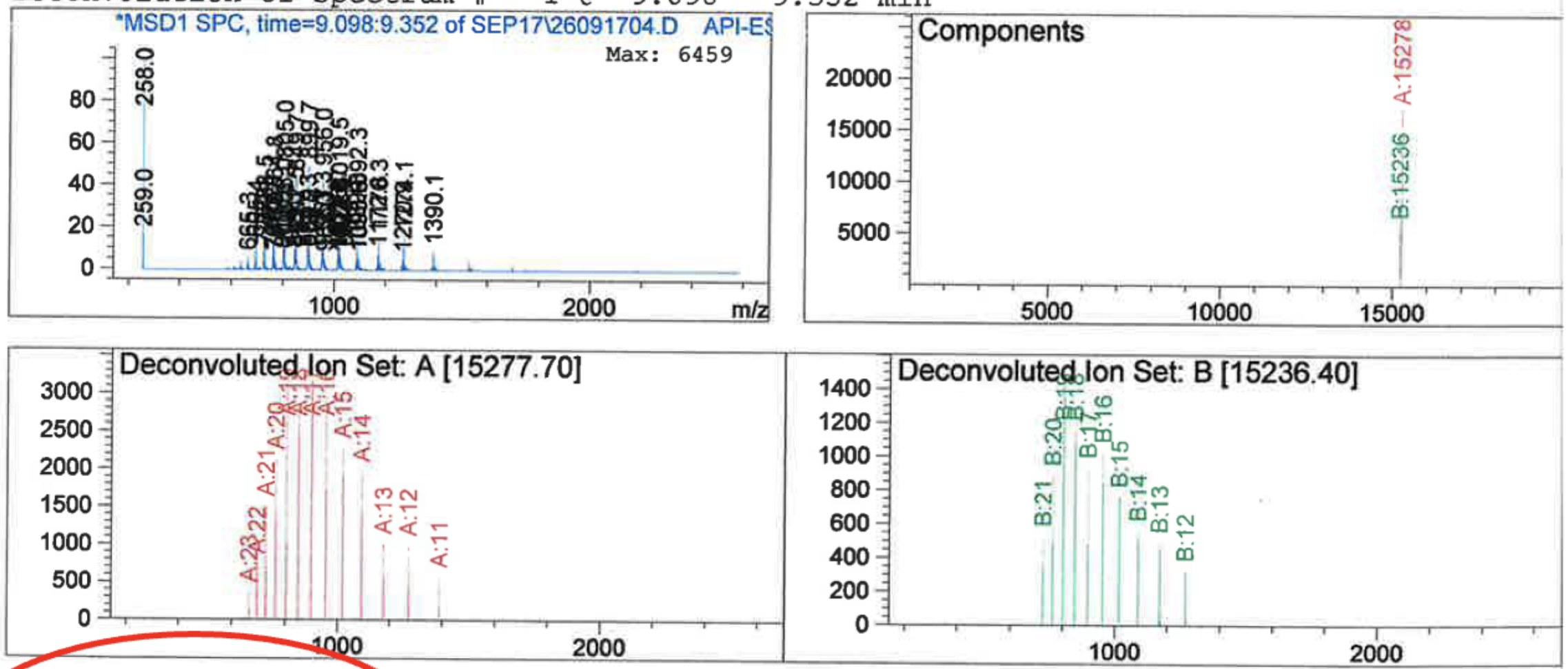


```
Component  Molecular    Absolute     Relative
             Weight     Abundance    Abundance
    A       15277.70      23448       100.00
    B       15236.40       7829        33.39 ?      = trimethylat?
                       *** End of Report ***
```

Appendix 1. Original validation of H3K36cMe3 by LC-MS. The expected molecular mass is 15,285.9 Da (15,199.74 + 87.166 - 1.0079). The observed mass of the major component A was 15,277.7 Da (~0.05% error). Minor component B was consistent with partial decomposition of the trimethylammonium substituent. Importantly, no trace of unmodified protein (15,199.74 Da) was not observed.

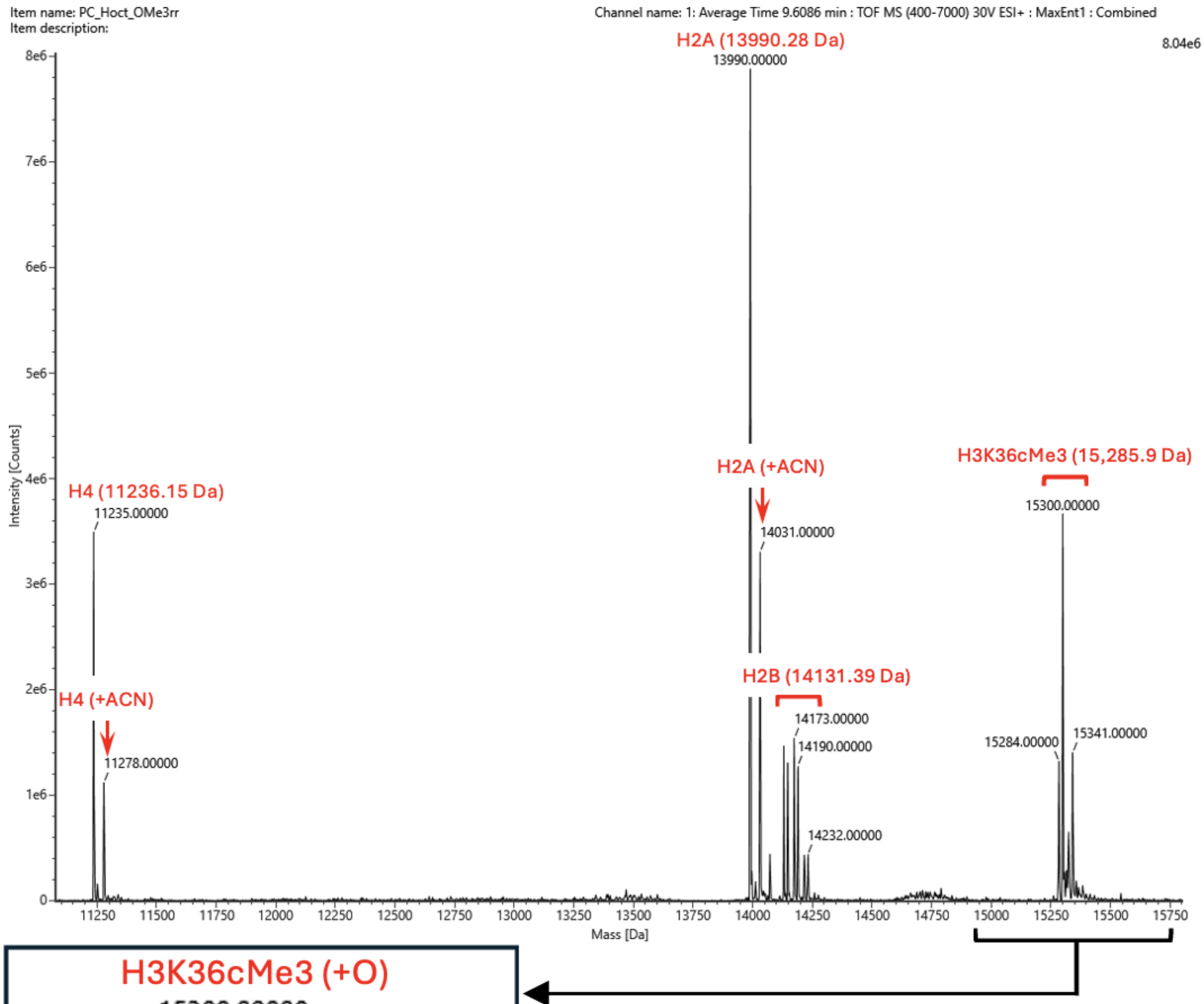

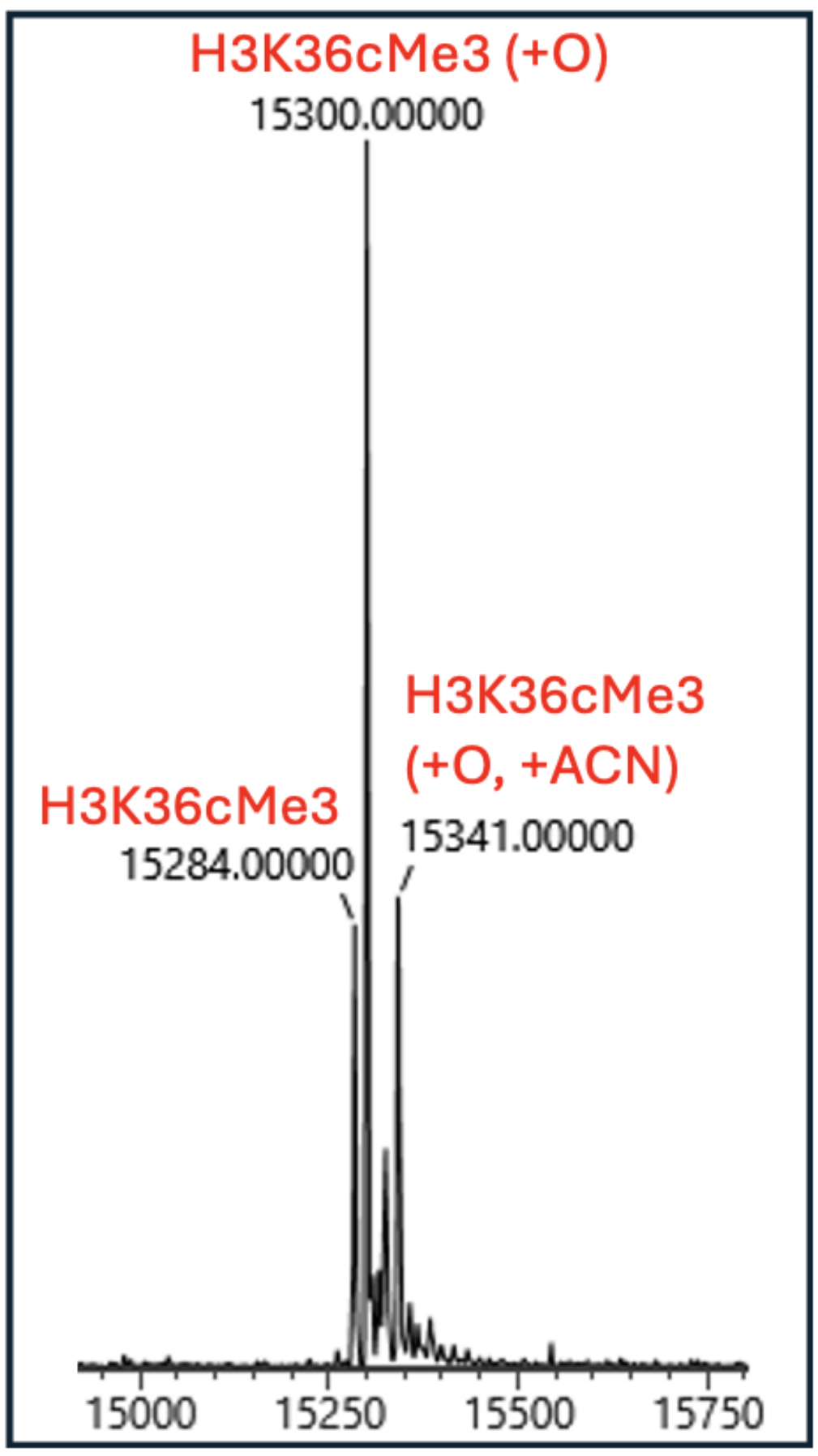

Appendix 2. Components of H3K36Me3A histone octamer analyzed by LC-MS upon prolonged storage. The octamer was dissociated by addition of 2% (v:v) formic acid and 2% (v:v) acetonitrile and analyzed by LC-MS (BioAccord, Waters). Data was acquired over a m/z range of 300 - 8000, in positive ion mode with a cone voltage of 40 v. Scans were summed and deconvoluted using MaxEnt1 (Masslynx, Waters).

Top: Deconvoluted mass spectrum showing all protein components. Peaks corresponding to individual histones are indicated.

Left: Zoomed view of peaks corresponding to H3K36cMe3, its oxidized form (+O), and the acetonitrile adduct (+O, +ACN). Notably, no trace of unmodified histone H3 K36C was detected.

We are happy to address the remaining concerns of Reviewers 4 and 6:

Reviewer 4, Q1: The concern about whether the chromatin arrays actually fold, compact, and stay accessible in comparable ways wasn't really addressed head-on. The authors point to integration controls as indirect evidence of similar chromatin structure, but that's not the same as measuring Mg^{2+}-dependent compaction or running accessibility assays directly. Functional outcomes are useful, but they can't fully stand in for biophysical measurements, especially when the mechanistic story leans so heavily on differences in chromatin engagement.

Affects of chromatin folding are ruled out by the original controls. Both K36Me0 and K36Me3 versions of the nucleosomal array importantly support the same level of integration when LEDGF is absent (for example see Fig 1d: 0 nM LEDGF condition) or when the PWWP domain is deleted (Fig 2a, left panels). By contrast, in the presence of full-length LEDGF (i.e. including the PWWP domain, which is a validated H3K36Me3 reader), there is a clear stimulation (Fig 1d: 2-200 nM LEDGF; Fig 2a, right set of panels). Should there be a difference in folding under (physiological) reaction conditions, there would be a clear difference in integration efficiency in the absence of LEDGF or its PWWP domain.

Reviewer 4, Q2. The ask was to compare H3K36 methylation against another histone site, something like H3K4me3 or H3K27me3, and that experiment wasn't done. The authors' argument that LEDGF/p75 and PWWP domain dependence is enough to establish specificity is reasonable as far as it goes, but it doesn't rule out that other methylation states could produce similar effects on accessibility or integration behaviour. Without an orthogonal methylation control, it's hard to be confident the effects are genuinely H3K36me3-specific rather than a more general consequence of histone methylation or shifts in chromatin structure.

The exact same logic in response to Q1 applies here as well: We have shown that in the presence of the PWWP H3K36Me3 reader (intact LEDGF), the intasome strongly selects H3K36Me3 chromatin, and in its absence, there is no selection: integration into Me3 is approximately as efficient as into Me0 (Figs 1d and 2a). If the hypothesis is that methylation results in non-specific effects, these would be apparent in the absence of the PWWP domain. Moreover, the effects are highly specific to native H3K36Me3, as the methyl-lysine analogue (the difference being a single non-hydrogen atom, C vs S) does not support the same level of stimulation (see Fig 1c: yellow bars; Fig 2a, green bars). Producing native H3K4Me3 or H3K27Me3 is possible but would require several months of new work. The effort is not justified, as we fail to see what alternative hypothesis could be ruled out by introducing an arbitrary modification given the controls already included.

Reviewer 4, Q3. Chromatin assembly quality is only partially characterized
The MNase digestion and AFM data are a step forward, but questions about nucleosome occupancy uniformity and possible partial unwrapping remain open. There's no quantitative demonstration that occupancy is consistent across templates, and the extent of nucleosome unwrapping isn't directly measured. This matters because the central claim, that LEDGF/p75 steers integration toward linker DNA, depends on being able to trust that linker accessibility isn't being shaped by variability in how the chromatin was assembled in the first place.

In the experiments with Widom 601 arrays, we show that integration sites accumulate inside linkers when nucleosomes are present. Ergo, there is a clear effect of nucleosome presence. In the integration site/nucleosome co-mapping experiments (using Fenton fragmentation in Fig

4b), the results are nucleosome-centric: We counted integration sites near a nucleosome. The AFM showed that the linkers of variable lengths are present. It does not matter if we under-saturated the yeast DNA: we were not trying to produce *in vivo*-like yeast chromatin. These experiments could have been done with random nucleosomes assembled on bacterial or phage DNA.

Reviewer 4, Q4. The "entropic filter" model is still more description than mechanism
The key experiment requested here was to mutate the acidic residues on the IBD surface and test whether they actually drive nucleosome exclusion. That wasn't done; it's been deferred to future work. Without it, the model stays correlative — the structural observations are interesting, but they don't demonstrate causality. Mutational data on those surface residues would be what moves this from a plausible structural story to a demonstrated mechanism.

Entropic filter, to our minds, is an elegant and appealing hypothesis we propose in Discussion. We specifically highlight that it will be a subject of follow up studies that will invariably include dedicated computational approaches such as coarse grain molecular dynamics. The mesh comprising sixteen copies of 530-residue LEDGF, including the IBD "bollards" along with the bulk of the unstructured segments and PWWP domains is what makes this filter. The reviewer is overly focused on the acidic residues on the IBD. Their location is intriguing, but we do not think mutating these residues will make a substantial difference as steric hinderance alone is likely the dominant effect. Should we do the experiment, either outcome will not be informative without a dedicated computational study.

Reviewer 4, Q5. How LEDGF occupancy shapes integration selectivity hasn't been tested
The rationale for using near-saturating LEDGF concentrations makes sense, but the question of what happens at lower, more variable occupancy levels was left unaddressed experimentally. Since the structural interpretation is built on highly occupied complexes, it would be worth knowing whether the linker-DNA preference holds up — or scales predictably — under conditions that better reflect the heterogeneity you'd expect in a cellular context. Titration or partial occupancy experiments would speak to that directly.

We have shown that at both the lowest and highest LEDGF input levels tested, which correspond to approximate 64 and 128 molar excess of LEDGF to intasomes, integration was strongly preferred into exit/linker regions of chromatin (Fig. 3c/f and Fig. 4b). LEDGF co-purifying with the intasome corresponds to approximately 3-5 molecules per intasome (Supplementary Fig. S1).

Reviewer 4, Q6. The cellular evidence
The ask was for experiments using chromatin-binding-deficient or structurally altered LEDGF variants to test integration-site selection in cells. The authors cite existing literature, which establish that the PWWP domain matters broadly, that's not in dispute. However, they weren't designed to test the specific model being proposed here about linker-DNA targeting and nucleosome exclusion. New cellular data would be needed to connect the in vitro mechanistic picture to what actually happens in cells.

We note that the original Q6 was phrased differently. Now, the reviewer is specifically asking us to show integration into linker DNA in cells. Given current technology, this is not possible.

Integration events in cells are incredibly rare, on the order of one event per human genome. Accordingly, it has not been possible to catch/visualize integration in action. Cellular

integration is identified by sequencing host-viral junctions after the events have taken place, typically days following infection. For this reason, all mechanistic studies have relied on bottom-up approaches (experiments *in vitro* using chromatinized DNA). This is an ongoing fundamental limitation of our field. Although mapping integration sites in cells is easily done, there is no method to map nucleosome positions as they were at the time of integration. Nucleosomes are highly mobile, in particular within active transcription units, where lentiviruses preferentially integrate.

Our study is the first to link the location of viral DNA integration to nucleosome positioning using two divergent lentiviral intasomes (HIV-1 and MVV) and chemical DNA fragmentation. We have validated our approaches using another well-characterized intasome, from PFV, that is known to integrate into core nucleosomes. We also showed for the first time that LEDGF is sufficient to direct integration into H3K36Me3 chromatin *in vitro*. Both of these results, alongside the cryo-EM structures, comprise a very significant advancement to the field.

Reviewer 6, Comment 1c: LEDGF/p75 has been shown to affect the nucleosomal barrier to transcription (LeRoy et al, Sci Adv 2019)...

Despite expectations, our experiments did not uncover massive infiltration of core nucleosomes by lentiviral integration in the presence of LEDGF. Even with LEDGF present at 2.5-fold excess to nucleosomes, integration still took place in the linker regions, although shifting to the middle of the array (Fig. 3f, red trace). The shift towards the middle of the array is by itself an original and very telling observation, as we discussed in the manuscript.

Even though both transcription and (lentiviral) integration are impeded by nucleosomes, these are completely different processes, and the modes of resolution are unlikely identical. Therefore, our fundings should not be considered at odds with the study by LeRoy *et al*.

Reviewer 6, Comment 3: the PICs bound with LEDGF/p75

The fact that cytoplasmic PICs could be co-immunoprecipitated with LEDGF does not have any bearing on our story, since the chromatin resides in the nucleus, which is where integration takes place.
